# Relationship Between Cardiometabolic Index in Early Pregnancy and Hypertensive Disorder Complicating Pregnancy

**DOI:** 10.7759/cureus.51598

**Published:** 2024-01-03

**Authors:** Zakir Ullah Khan, Shakir Ullah Khan, Musaira Tariq, Waqar Mustafa, Humayun Saleem, Amna Akbar, Sarosh Khan Jadoon, Sabahat Tasneem

**Affiliations:** 1 Cardiology, Russells Hall Hospital, Dudley, GBR; 2 Trauma and Orthopedics, University Hospital Southampton NHS Foundation Trust, Southampton, GBR; 3 Gynecology, Hayatabad Medical Complex Peshawar, Peshawar, PAK; 4 Cardiology, Combined Military Hospital, Muzaffarabad, PAK; 5 Public Health, Health Services Academy, Muzaffarabad, PAK; 6 Emergency and Accident, District Headquarter Jhelum, Muzaffarabad, PAK; 7 General Surgery, Combined Military Hospital, Muzaffarabad, PAK; 8 Public Health, Health Services Academy, Islamabad, PAK

**Keywords:** hdcp, preeclampsia, hypertension, cardiometabolic index, pregnancy

## Abstract

Background

This study aimed to examine the cardiometabolic index during early pregnancy in individuals with hypertension-complicating pregnancy, especially preeclampsia. Additionally, this study sought to determine the relationship between cardiometabolic index and the incidence of varying degrees of preeclampsia.

Methodology

This study included 289 pregnant women diagnosed with preeclampsia who were registered and delivered at our hospital. These women were assigned to the preeclampsia group. Additionally, a group of 289 healthy pregnant women of identical gestational ages within the same time frame was included for comparison. Clinical data on pregnancy, including body mass index (BMI), blood pressure, waistline, triglyceride levels, and cardiometabolic index, were compared between the two groups. An analysis was conducted to examine the association between early pregnancy cardiometabolic index and the occurrence of preeclampsia.

Results

There was a significant association between the quartile of cardiometabolic index and the proportion of preeclampsia patients (p < 0.001). Furthermore, after controlling for age and BMI, the risk of preeclampsia remained significantly elevated and was associated with the cardiometabolic index.

Conclusions

A positive correlation was observed between cardiometabolic index during early pregnancy and the occurrence of preeclampsia.

## Introduction

Hypertensive disorder complicating pregnancy (HDCP) is a medical condition characterized by co-occurrence of pregnancy and increased blood pressure. This condition is recognized as a significant factor contributing to unfavorable outcomes in pregnant women [1]. Currently, there is a lack of clarity regarding the pathophysiology of the disease. Previous studies have indicated a strong correlation between extensive endothelial cell injury and dysfunction in hypertensive disorders of pregnancy. Aberrant lipid metabolism might induce vascular endothelial cell injury, contributing to the development of this pathological condition [2]. Preeclampsia is a significant form of HDCP that is distinguished by the onset of hypertension and proteinuria after the 20th week of gestation. It is a crucial contributor to both maternal and neonatal mortality [3]. Research findings indicate that the prevalence of preeclampsia ranges from 3% to 8% and increases the risk of pregnancy-associated stroke [4]. The prevalence of pregnancy hypertension reported for Pakistan was 9.3% and that for preeclampsia was 2.4% in a multicenter study [5]. A tertiary care study in Sindh, Pakistan, reported a 5.6% incidence of preeclampsia and eclampsia [6].

Hence, it is imperative to identify and manage risk factors associated with preeclampsia. The presence of aberrant blood lipid indices, specifically triglycerides (TG) and total cholesterol (TC), during the early stages of pregnancy, has been shown to have predictive value for the development of preeclampsia [7]. The cardiometabolic index is a novel metric initially introduced by Wakabayashi et al. in 2015 [8]. This index is calculated using the waist-to-height ratio (WHtR), TG, and high-density lipoprotein cholesterol (HDL-C), which are commonly available in clinical settings [9]. Consequently, this composite score integrates the measures of abdominal obesity and lipid-related factors.

Empirical research has demonstrated the ability of the cardiometabolic index to anticipate alterations in left ventricular geometry [10], the occurrence of stroke [11], the incidence of diabetes [12], and various other medical conditions. Furthermore, it can provide an estimation of the impact of obesity on the prevalence of hypertension [13]. Nevertheless, there is scarce literature on the correlation between cardiometabolic indices and hypertensive disease during pregnancy. This study aimed to examine the association between cardiometabolic index in early pregnancy and the occurrence of preeclampsia. The findings of this research can contribute to the development of guidelines for preventing and managing hypertensive diseases in pregnancy as well as inform the formulation of public health policies in this regard.

## Materials and methods

Study population and duration

This study retrospectively included 289 pregnant women diagnosed with preeclampsia who were registered and delivered at our hospital between June 2020 and May 2023 and were categorized into the preeclampsia group. Additionally, a group of 289 healthy pregnant women of identical gestational ages within the same time frame was selected for comparison.

Inclusion and exclusion criteria

For pregnant women to be included in the study, several conditions needed to be met. First, the preeclampsia group had to satisfy the diagnostic criteria for preeclampsia, as outlined in a previous study [14]. Second, all pregnant women included in the study had singleton pregnancies. Lastly, it was required that the women had not taken any medications that could potentially impact their blood lipid profiles such as omega-3 fatty acids during pregnancy. Pregnant women were ineligible for enrollment under the following circumstances: (1) complications related to chronic hypertension disorders, immune system diseases, or endocrine diseases; (2) severe liver and kidney dysfunction; and (3) concurrent malnutrition, diabetes, or heart disease.

Ethical approval

This study underwent a thorough evaluation and was approved by the Medical Ethics Committee affiliated with the Abbas Institute of Medical Sciences (ERB number: 4608). The data were collected from the hospital database; therefore, informed consent was not obtained from patients.

Data collection

The indicator level during the initial trimester corresponds to the period preceding 14 weeks of gestation. Basic patient information was obtained from records, such as height, weight, waistline, and age. A biochemical and immunological analyzer and its assay kit were used to measure the levels of TC, TG, HDL-C, and low-density lipoprotein cholesterol (LDL-C). The cardiometabolic index was calculated using the formula TG/HDL-C × WHtR [15]. The WHtR was calculated by dividing the waistline measurement (in centimeters) by the individual’s height. In contrast, the TG/HDL-C ratio was calculated by dividing the value of TG by HDL-C. Pregnant women were categorized into four groups based on the quartiles of their cardiometabolic indices. Body mass index (BMI) was calculated using the formula BMI = weight (in kilograms)/height (in meters)^2^ [16]. Three measurements of blood pressure were noted, and their average was calculated and noted as the systolic and diastolic blood pressure.

Data analysis

The data were processed using SPSS version 25.0 (IBM Corp., Armonk, NY, USA). The data for continuous variables, which followed a normal distribution, were reported as the mean ± standard deviation (SD). Subsequently, a paired-sample t-test was conducted to compare variables between the two groups. Categorical data were represented as the number of cases given as frequencies and percentages and were analyzed using the chi-square test. Logistic regression analysis was employed to assess the independent association between the cardiometabolic index and preeclampsia while controlling for confounding factors. Odds ratios (ORs) and their accompanying 95% confidence intervals (Cls) were used for this purpose. The chi-square test was used to examine linear trends in the quartiles of the cardiometabolic index. All statistical analyses conducted in this study were performed using two-sided tests, and the significance level was set at p ≤ 0.05.

## Results

The baseline characteristics of the patients including age, height, waistline, weight, BMI, blood groups, and other details regarding pregnancy are presented in Table 1.

**Table 1:** Baseline data. The p-value is considered significant at p < 0.05. BMI: body mass index; SBP: systolic blood pressure; DBP: diastolic blood pressure

Variables		PG (n = 289)	CG (n = 289)	t-value	P-value
Age (years)		32.04 ± 2.92	28.3 ± 3.22	14.41	0.000
Height (cm)		159.10 ± 2.04	158.18 ± 3.3	-4.24	0.000
Weight (kg)		66.73 ± 5.71	61.62 ± 3.65	12.04	0.000
BMI (kg/m^2^)		26.70 ± 2.55	24.35 ± 1.60	12.65	0.000
Waistline (cm)		79.33 ± 5.17	73.94 ± 5.02	12.91	0.000
Gestational age (weeks)		12.88 ± 1.07	12.98 ± 0.77	-1.52	0.129
Parity (times)	0	152 (52.6%)	133 (46.0%)	-1.676	0.095
	1	114 (39.4%)	134 (46.4%)		
	2	23 (8.0%)	22 (7.6%)		
History of cesarean section	Have	38 (13.1%)	5 (1.7%)	5.399	0.000
	Not have	251 (86.9%)	284 (98.3%)		
History of miscarriages	Have	11 (3.8%)	1 (0.3%)	2.924	0.004
	Not have	278 (96.2%)	288 (99.7%)		
SBP (mmHg)		140.21 ± 3.74	131.45 ± 4.76	24.91	0.000
DBP (mmHg)		89.57 ± 6.52	80.43 ± 5.77	17.64	0.000
Blood type	A	113 (39.1%)	93 (32.2%)	1.248	0.213
	B	70 (24.2%)	79 (27.3%)		
	AB	67 (23.2%)	65 (22.5%)		
	O	39 (13.5%)	52 (18.0%)		

Table 1 demonstrates that the preeclampsia group exhibited higher values (p < 0.05) for age, height, weight, BMI, waistline, proportion of cesarean section history and history of miscarriages, systolic blood pressure (SBP), and diastolic blood pressure (DBP) than the control group.

The laboratory parameters including platelet count (PLT), white blood cell count (WBC), aspartate aminotransferase (AST), alanine aminotransferase (ALT), creatinine (Cr), uric acid (UA), fasting blood glucose (FBG), magnesium (Mg), chloride (Cl), thyroid-stimulating hormone (TSH), neutrophil count, lymphocyte count, hemoglobin, hematocrit, albumin, total bilirubin, free thyroxine, serum potassium, and blood calcium are presented in Table 2.

**Table 2:** Laboratory parameters. The p-value is considered significant at p < 0.05. PLT: platelet count; WBC: white blood cell count; AST: aspartate aminotransferase; ALT: alanine aminotransferase; Cr: creatinine; UA: uric acid; FBG: fasting blood glucose; Mg: magnesium; Cl: chloride; TSH: thyroid-stimulating hormone

Index	PG (n = 289)	CG (n = 289)	t-value	P-value
WBC (×10^9^/L)	9.90 ± 1.52	8.70 ± 1.51	9.78	0.000
Neutrophil count (×10^9^/L)	6.33 ± 1.16	6.31 ± 1.46	0.17	0.863
Lymphocyte count (×10^9^/L)	1.99 ± 0.40	2.00 ± 0.57	-0.16	0.867
Haemoglobin (g/L)	124.44 ± 7.23	123.63 ± 13.24	0.94	0.343
Haematocrit (%)	35.07 ± 1.21	34.57 ± 1.21	5.21	0.000
PLT (×10^9^/L)	231.27 ± 33.32	214.18 ± 33.24	6.13	0.000
Albumin (g/L)	41.57 ± 1.16	41.38 ± 1.18	-1.97	0.050
Total bilirubin (mol/L)	10.66 ± 1.97	10.01 ± 1.97	-3.94	0.000
AST (U/L)	17.33 ± 3.08	15.34 ± 3.08	7.42	0.000
ALT (U/L)	16.33 ± 3.95	11.33 ± 3.92	15.56	0.000
Cr (mol/L)	44.07 ± 9.66	45.53 ± 9.66	-1.68	0.093
Urea (mmol/L)	3.02 ± 0.32	2.90 ± 0.32	4.55	0.000
UA (mol/L)	200.09 ± 21.58	181.99 ± 21.59	9.94	0.000
FBG (mmol/L)	4.64 ± 0.64	4.57 ± 0.64	1.33	0.182
Mg (mmol/L)	0.85 ± 0.05	0.84 ± 0.05	2.21	0.028
Cl (mmol/L)	103.51 ± 1.29	102.52 ± 1.29	8.79	0.000
TSH (mIU/L)	1.38 ±0.38	1.29 ± 0.38	2.69	0.008
Free thyroxine (ng/dL)	1.18 ± 0.07	1.18 ± 0.08	-0.15	0.874
Serum potassium (mmol/L)	4.10 ± 0.14	4.10 ± 0.14	0.10	0.913
Blood calcium (mmol/L)	2.27 ± 0.09	2.28 ± 0.09	-0.14	0.884

Table 2 demonstrates that the preeclampsia group exhibited greater WBC, hematocrit, PLT, AST, ALT, albumin, total bilirubin, UA, urea, Mg, CI, and TSH levels (p < 0.05). The preeclampsia group had significantly higher levels of TC, TG, HDL-C, LDL-C, WHtR, and cardiometabolic index than the control group (p < 0.05) (Table 3).

**Table 3:** Blood lipid index and cardiometabolic index. The p-value is considered significant at p < 0.05. TC: total cholesterol; TG: triglycerides; HDL-C: high-density lipoprotein cholesterol; LDL-C: low-density lipoprotein cholesterol; WHtR: waist-to-height ratio

Index	PG (n = 289)	CG (n = 289)	t-value	P-value
TC (mmol/L)	4.62 ± 0.42	4.45 ± 0.42	4.92	0.000
TG (mmol/L)	1.56 ± 0.41	1.29 ± 0.41	7.96	0.000
HDL-C (mmol/L)	1.74 ± 0.16	1.69 ± 0.15	-3.84	0.000
LDL-C (mmol/L)	2.43 ±0.36	2.21 ± 0.36	-7.08	0.000
WHtR	0.50 ± 0.03	0.46 ± 0.03	-15.01	0.000
Cardiometabolic index	0.43 ± 0.12	0.37 ± 0.12	5.43	0.000

Age (in years), higher BMI (kg/m²), higher SBP and DBP, history of cesarean section, history of miscarriages, and higher cardiometabolic index were all risk factors for preeclampsia (Tables 4, 5).

**Table 4:** Test statistics for tests of associations of continuous and categorical variables with preeclampsia variables. The p-value is considered significant at p < 0.05. ANOVA: analysis of variance; BMI: body mass index; SBP: systolic blood pressure; DBP: diastolic blood pressure

Variables	Mean square		Sig.
One-way ANOVA test variables	Between groups	Within groups	
Age (years)	2,044.237	9.455	0.000
BMI (kg/m²)	799.976	4.549	0.000
Gestational age (weeks)	1.557	0.874	0.182
Parity	0.561	0.399	0.236
SBP	11,091.737	18.349	0.000
DBP	12,067.268	37.968	0.000

**Table 5:** Test statistics for tests of associations of categorical variables with preeclampsia variables.

Test variables		No preeclampsia	Preeclampsia	
Blood group	A	79	70	0.224
	B	65	67	
	AB	52	39	
	O	93	113	
History of cesarean section	Absent	288	278	0.006
	Present	1	11	
History of miscarriages	Absent	284	251	0.000
	Present	5	38	
Parity	1st	133	152	0.234
	2nd	134	114	
	3rd	22	23	
Cardiometabolic index quartiles	Q1	93	51	0.000
	Q2	75	75	
	Q3	73	67	
	Q4	48	96	

Higher values of WBC, hematocrit, PLT, AST, ALT, albumin, total bilirubin, UA, urea, Mg, CI, and TSH were all risk factors (Table 6). The variables TC, TG, HDL-C, and LDL-C in lipid index, WHtR, and cardiometabolic index demonstrated a significant association with the risk of preeclampsia (p < 0.05) (Table 7).

**Table 6:** One-way ANOVA for laboratory index The p-value is considered significant at p < 0.05. PLT: platelet count; WBC: white blood cell count; AST: aspartate aminotransferase; ALT: alanine aminotransferase; Cr: creatinine; UA: uric acid; FBG: fasting blood glucose; Mg: magnesium; Cl: chloride; TSH: thyroid-stimulating hormone

Variables	Mean square		Sig.
	Between groups	Within groups	
WBC (×10^9^/L)	209.282	2.324	0.000
Neutrophil count (×10^9^/L)	0.055	1.744	0.859
Lymphocyte count (×10^9^/L)	0.007	0.249	0.865
Hemoglobin (g/L)	94.801	113.908	0.362
Hematocrit (%)	36.125	1.484	0.000
PLT (×10^9^/L)	42,178.04	1,107.891	0.000
Albumin (g/L)	5.371	1.379	0.049
Total bilirubin (μmol/L)	61.051	3.889	0.000
AST (U/L)	576.002	9.508	0.000
ALT (U/L)	3,607.502	15.514	0.000
Cr (μmol/L)	306.252	93.448	0.071
Urea (mmol/L)	2.141	0.107	0.000
UA (μmol/L)	47,363.18	466.254	0.000
FBG (mmol/L)	0.708	0.418	0.194
Mg (mmol/L)	0.014	0.003	0.026
Cl (mmol/L)	141.516	1.685	0.000
TSH (mI.U/L)	1.043	0.149	0.008
Free thyroxine (ng/dL)	0.000	0.006	0.873
Serum potassium (mmol/L)	0.000	0.022	0.911
Blood calcium (mmol/L)	0.000	0.01	0.882

**Table 7:** One-way ANOVA for lipid and cardiometabolic index. The p-value is considered significant at p < 0.05. ANOVA: analysis of variance; TC: total cholesterol; TG: triglycerides; HDL-C: high-density lipoprotein cholesterol; LDL-C: low-density lipoprotein cholesterol; WHtR: waist-to-height ratio

Variables	Mean square		Sig.
	Between groups	Within groups	
TC (mmol/L)	4.152	0.178	0.000
TG (mmol/L)	10.327	0.171	0.000
HDL-C (mmol/L)	0.368	0.026	0.000
LDL-C (mmol/L)	7.009	0.134	0.000
WHtR	0.208	0.001	0.000
Cardiometabolic index	0.441	0.016	0.000

The individuals’ cardiometabolic index levels were categorized into quartiles, with values of 0.281, 0.424, and 0.508 for the first, second, and third quarters, respectively. Individuals with a cardiometabolic index equal to or less than the first quartile were assigned to Group 1 (Q1). Those with a cardiometabolic index greater than Q1 and equal to or less than the second quartile were assigned to Group 2 (Q2). Similarly, individuals with a cardiometabolic index greater than Q2 and equal to or less than the third quartile were assigned to Group 3 (Q3). Finally, individuals with a cardiometabolic index greater than Q3 were assigned to Group 4 (Q4). The prevalence of preeclampsia in the study participants was found to be 35.4% (51 out of 144) in the Q1 group, 50% (75 out of 150) in the Q2 group, 47.8% (67 out of 140) in the Q3 group, and 66.7% (96 out of 144) in the Q4 group.

**Figure 1:**
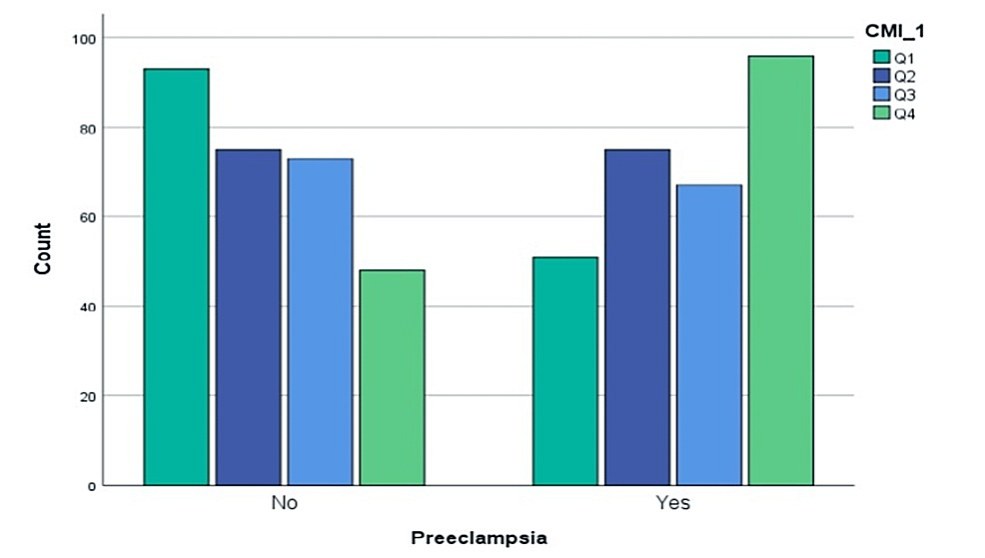
The prevalence of preeclampsia patients in association with quartiles.

There was a significant positive correlation (p < 0.05) between the cardiometabolic index quartile and the number of preeclampsia patients, indicating that as the quartile increased, the proportion of preeclampsia patients also increased. The findings of this study demonstrate a significant correlation between cardiometabolic index and preeclampsia prevalence. As seen in Figure 1, the prevalence of preeclampsia was notably associated with the ascending quartiles of the cardiometabolic index (p < 0.001), demonstrating an upward trajectory from Q1 to Q2. The trend was followed by Q4, although Q3 deviated slightly (Figure 1).

Age, BMI, and cardiometabolic index were significantly correlated with the development of preeclampsia on binary logistic regression analysis. Logistic regression analysis was conducted to further examine the relationship between the cardiometabolic index and preeclampsia, with age and BMI as risk factors for preeclampsia (Table 8).

**Table 8:** Subgroup analysis of cardiometabolic index in preeclampsia The p-value is considered significant at p < 0.05. CMI: cardiometabolic index; BMI: body mass index

Variable	Odds ratio	95% confidence interval (CI)	Sig.
Age	5.262	3.686–7.509	0.000
BMI	6.385	4.445–9.170	0.000
Parity_1	0.744	0.530–1.047	0.090
Parity_2	0.915	0.487–1.716	0.781
CMI Quartiles_1	1.823	1.142–2.912	0.012
CMI Quartiles_2	1.673	1.039–2.693	0.034
CMI Quartiles_3	3.647	2.242–5.932	0.000
Constant	0.548		0.001

The relationship between cardiometabolic index and preeclampsia was positive even after adjusting for confounders (Table 9).

**Table 9:** Logistic regression analysis of cardiometabolic index and preeclampsia. CMI: cardiometabolic index; BMI: body mass index; OR: odds ratio

	Unadjusted OR	OR adjusted with age	OR adjusted with BMI	OR adjusted with age and BMI
CMI Quartiles_1	3.647 (2.242–5.932)	4.781 (2.786–8.206)	3.794 (2.214–6.502)	5.183 (2.835–9.475)
CMI Quartiles_2	2.00 (1.248–3.205)	2.323 (1.385–3.896)	1.904 (1.131–3.205)	2.184 (1.240–3.844)
CMI Quartiles_3	2.179 (1.349–3.520)	2.679 (1.579–4.545)	2.272 (1.335–3.865)	2.759 (1.560–4.878)
CMI Quartiles_4	–		–	

## Discussion

Preeclampsia is a condition of unknown cause that occurs in pregnant women and affects approximately 4% of all pregnancies. It can affect many organ systems in the body [17], which can result in significant negative outcomes in both mothers and infants. The exact cause of this development has not been conclusively established. However, clinical research has demonstrated that vascular endothelial damage plays a crucial role in its occurrence [18]. Preeclampsia is characterized by elevated blood pressure in pregnant women. Research has indicated that characteristics such as age 35 years or older, gestational weight, number of previous pregnancies, pre-pregnancy BMI of 28 kg/m^2^ or higher, serum Cr level, and mean arterial pressure are commonly associated with the development of preeclampsia [19].

The incidence of preeclampsia is influenced by TG and TC levels, whereas the cardiometabolic index is derived from the abdominal obesity index and lipid-related index. Thus, we postulated that there might be a correlation between cardiometabolic index and the occurrence of preeclampsia. The study revealed that women with preeclampsia had higher values than healthy pregnant women in terms of age, weight, BMI, waistline, the proportion of previous cesarean sections, history of miscarriages, SBP, DBP, WBC, hematocrit, PLT, AST, ALT, albumin, total bilirubin, UA, urea, Mg, CI, and TSH. The variable values of TC, TG, HDL-C, LDL-C, WHtR, and cardiometabolic index increased in preeclampsia patients. These findings indicate that cardiometabolic index may predict the risk of onset of preeclampsia at an early gestational age. Further analysis discovered a distinct connection between the cardiometabolic index and the occurrence of preeclampsia, demonstrating a positive correlation between the two. These findings suggest that the cardiometabolic index can serve as a reliable marker for early detection of preeclampsia and enhance the understanding and management of preeclampsia in expecting mothers.

An observational study discovered that the TG level in the blood serum of individuals with hypertension was notably greater than that in individuals without hypertension [20]. Pregnant women with elevated TG levels and reduced HDL-C levels may be at a greater risk of developing dyslipidemia. This condition can negatively affect placental function, causing damage and apoptosis of placental vascular cells, ultimately leading to the development of preeclampsia [21]. Pregnant women typically undergo a sequence of adjustments in lipid metabolism during pregnancy [22]. The primary pathogenic alteration observed in preeclampsia is arterial spasm [23]. There is excessive growth of smooth muscle cells in the blood vessel walls. This induces vasoconstriction, resulting in the build-up of a significant quantity of lipids in the inner lining of blood arteries. This exacerbates the harm to vascular endothelial cells and leads to spasms in the arteries of pregnant women [22]. The lipid indices TC, TG, HDL-C, LDL-C, and WHtR were all significantly different in our study cohorts.

Research has indicated a strong correlation between maternal obesity and preeclampsia [24]. Yang et al. [25] demonstrated that obesity is a significant risk factor for preeclampsia, indicating the influence of lifestyle- and health-related factors. Preeclampsia is an inflammatory disorder that occurs during pregnancy [26]. Pregnant women who are obese are more likely to develop preeclampsia, potentially because placental growth is hindered by changes in metabolic balance [27]. Systemic inflammation, hypertension, and other negative consequences related to preeclampsia were found to be related to inflammatory cytokines originating from the mother’s adipose tissue and circulating cholesterol [28].

Strengths and limitations

This study demonstrated that the cardiometabolic index can be utilized as a predictive tool for assessing the likelihood of developing preeclampsia. This finding establishes a foundation for promoting health and implementing preventive measures to manage and control pre-eclampsia. Furthermore, this study employed hierarchical analysis to mitigate potential research bias by examining several affecting elements. Nevertheless, this study has several limitations. Initially, this established a correlation between the cardiometabolic index and preeclampsia without establishing a causative relationship. Furthermore, this study was conducted at a single center and had a limited population size, thereby limiting the generalizability of the results to pregnant women from different geographical areas. Hence, future research should focus on conducting multicenter studies to improve the dependability of the study’s findings.

## Conclusions

The lipid indices including TC, TG, HDL-C, LDL-C, cardiometabolic index, and WHtR values were higher in the preeclampsia group, and there was a significant difference from the normal cohort that we observed in the comparison of preeclampsia females. There was a direct correlation between cardiometabolic index during early pregnancy and the likelihood of developing preeclampsia. These findings suggest that the study of cardiometabolic index can help to identify risk factors in pregnant females and there is a probability of cardiometabolic index to be used as a diagnostic biomarker.

## Appendices

**Table 10:** Data file. Laboratory index. PLT: platelet count; WBC: white blood cell count; AST: aspartate aminotransferase; ALT: alanine aminotransferase; Cr: creatinine; UA: uric acid; FBG: fasting blood glucose; Mg: magnesium; Cl: chloride; TSH: thyroid-stimulating hormone

WBC (×10^9^/L) PG	WBC (×10^9^/L) CG	Neutrophil count (×10^9^/L) PG	Neutrophil count (×10^9^/L) CG	Lymphocyte count (×10^9^/L) PG	Lymphocyte count (×10^9^/L) CG	Hemoglobin (g/L) PG	Hemoglobin (g/L) CG	Hematocrit (%) PG	Hematocrit (%) CG	PLT (×10^9^/L) PG	PLT (×10^9^/L) CG	Albumin (g/L) PG	Albumin (g/L) CG	Total bilirubin (μmol/L) PG	Total bilirubin (μmol/L) CG	AST(U/L) PG	AST (U/L) CG	ALT (U/L) PG	ALT (U/L) CG	Cr (μmol/L) PG	Cr (μmol/L) CG	Urea (mmol/L) PG	Urea (mmol/L) CG	UA (μmol/L) PG	UA (μmol/L) CG	FBG (mmol/L) PG	FBG (mmol/L) CG	Mg (mmol/L) PG	Mg (mmol/L) CG	Cl (mmol/L) PG	Cl (mmol/L) CG	TSH (mIU/L) PG	TSH (mIU/L) CG	Free thyroxine (ng/dL) PG	Free thyroxine (ng/dL) CG	Serum potassium (mmol/L) PG	Serum potassium (mmol/L) CG	Blood calcium (mmol/L) PG	Blood calcium (mmol/L) CG
12.1	8.3	4.42	6.2	2.63	2.47	125.6	125.4	35.2	33.65	227.53	236.36	40.82	39.53	9.72	10.2	12	21	20	8	42.05	45.25	3.3	3.08	232.0	209.3	5.2	4.59	0.96	0.91	107	104	1.34	1.25	1.36	1.29	4.31	4.35	2.21	2.37
9.5	7.1	4.42	8	1.36	2.69	113.6	116.3	34.75	33.92	281.23	210.53	39.52	40.97	13.25	12.91	12	16	15	11	43.73	46	2.8	2.38	212.3	204.0	4.66	4.89	0.79	0.88	102	104	1.12	2.06	1.18	1.23	4.09	3.91	2.36	2.28
9.8	11.3	6.32	6.2	1.4	2.94	125.4	118.2	35.25	34.7	210.5	260.86	42.38	42.53	11.85	12.5	14	15	19	15	44.8	45.18	2.9	3.48	182.1	195.9	4.8	4.59	0.81	0.91	103	102	1.19	1.03	1.23	1.1	3.91	4.24	2.22	2.22
9.5	11.7	8.12	4.3	2.73	2.94	119.6	125.4	34.42	34.09	227.53	166.22	42.21	42.77	12.64	8.3	18	13	22	15	43.73	46.42	3.2	2.58	168.9	150.9	4.91	4.84	0.96	0.78	103	102	1.34	1.10	1.08	1.16	3.96	3.96	2.23	2.46
12.9	10	6.32	4.97	1.69	2.84	121.6	129.4	37.49	36.99	277.86	198.75	42.58	39.67	12.55	9.07	17	16	12	7	40.3	74.5	3.4	3.08	222.0	194.3	4.66	4.73	0.79	0.8	103	101	1.12	2.05	1.23	1.23	3.91	3.91	2.46	2.18
9.8	7	4.42	4.3	1.75	2.54	113.6	118.2	33.7	33.65	220.19	198.64	42.38	39.53	8.42	10.37	14	20	19	7	38.05	41.75	2.5	3.28	191.8	204.0	5.2	4.86	0.81	0.82	105	105	2.15	1.25	1.1	1.08	4.23	4.12	2.22	2.22
9.5	8.6	5.09	4.97	1.4	2.44	136	119.6	35.2	35.06	210.5	236.36	40.81	42.36	13.25	8.3	18	20	8	9	43.73	42.36	2.8	2.38	168.9	164.1	4.8	4.92	0.92	0.91	103	102	1.12	2.07	1.23	1.1	3.96	3.96	2.18	2.36
11.2	7.7	4.42	4.3	1.69	2.14	142	114.9	34.42	33.2	215.64	264.23	42.21	42.77	9.72	7.7	22	15	20	15	42.05	14.75	3.2	2.78	168.9	194.3	4.93	4.59	0.87	0.78	105	101	1.34	1.11	1.14	1.23	3.96	3.91	2.22	2.34
8.9	7.1	5.09	6.44	1.36	2.44	140	139	33.7	33.92	227.53	260.86	42.38	42.73	9.55	12.51	17	11	22	10	47.1	43.5	2.7	3.18	222.0	206.1	4.8	5.13	0.83	0.93	103	104	1.13	1.03	1.16	1.14	4.24	4.09	2.21	2.22
11.2	9.5	4.42	4.3	1.4	2.34	136	128	34.75	34.7	277.86	213.15	40.82	39.75	7.65	10.37	18	10	9	15	43.73	48.55	2.5	2.58	168.9	195.9	4.66	4.84	0.79	0.86	103	102	2.15	1.25	1.1	1.16	3.96	3.91	2.18	2.21
9.5	7	6.56	6.34	1.4	2.54	155	145	35.25	33.2	210.5	203.19	39.6	40.97	8.76	9.07	18	16	18	7	42.05	45.18	3.2	3.08	212.3	164.1	4.96	4.86	0.92	0.88	103	102	1.19	1.08	1.23	1.23	4.12	4.16	2.36	2.36
9.8	6.5	4.42	6.2	1.42	2.34	136	132	33.7	36.99	235.74	210.53	42.58	42.53	11.86	7.7	18	16	13	7	38.05	46	3.3	2.58	168.9	204.0	3.16	3.09	0.89	0.8	102	105	1.12	2.06	1.1	1.1	3.91	3.91	2.46	2.46
8.2	10.9	6.46	6.34	2.12	2.44	175	175	34.42	36.74	220.19	126.75	39.38	42.36	9.78	9.41	22	17	22	10	43.73	45.25	2.9	2.78	168.9	194.3	4.66	4.59	0.81	0.91	103	102	1.34	1.10	1.23	1.39	4.16	3.96	2.28	2.18
10.7	8.6	6.32	6.2	1.75	3.04	142	112	34.75	33.2	227.53	236.36	42.21	42.53	13.25	10.7	18	12	15	15	43.8	43.25	3.2	3.28	212.3	173.8	5.2	4.59	0.79	0.82	103	101	1.12	1.11	1.14	1.23	3.91	4.24	2.22	2.22
11.2	8.3	6.46	6.97	1.4	2.74	169	119	33.7	35.89	210.5	260.86	40.82	41.73	11.85	7.7	19	16	9	7	46.38	43.62	2.7	3.08	191.8	150.9	5.14	4.82	0.79	0.84	105	104	1.17	1.03	1.29	1.36	3.96	3.96	2.18	2.21
12.1	7.7	6.32	4.97	1.36	1.74	132.6	113.6	37.49	33.65	277.86	218.74	42.62	40.96	9.72	12.5	18	20	12	15	38.05	47.35	3.6	3.48	182.1	164.1	4.8	4.73	0.81	0.78	105	102	1.19	1.08	1.1	1.1	3.91	3.91	2.36	2.33
9.8	10	7.09	5.02	1.55	2.74	125.4	125.4	34.42	33.2	215.64	198.75	40.81	42.53	9.55	10.2	18	15	9	15	43.73	41.75	2.7	3.18	222.0	204.0	4.66	5.13	0.87	0.91	103	104	1.34	1.25	1.23	1.23	4.12	4.31	2.46	2.22
11.2	11.7	5.09	6.82	1.4	2.54	118.2	119.6	33.7	33.92	183.22	166.22	42.38	40.97	13.25	10.37	18	15	20	9	38.05	43.5	3.2	3.28	224.1	150.9	4.93	3.09	0.85	0.91	106	102	2.15	1.05	1.16	1.14	3.91	3.91	2.33	2.18
8.2	5.9	6.32	7.48	1.36	2.44	125.4	121.6	34.75	34.7	210.5	198.64	40.82	42.73	7.65	9.07	12	11	20	7	43.73	45.18	2.8	2.58	212.3	194.3	4.93	4.86	0.81	0.78	103	102	1.12	1.11	1.23	1.08	4.31	4.12	2.18	2.37
11.2	7.1	6.32	4.59	1.69	2.14	116.3	113.6	34.59	33.2	220.19	260.86	41.58	39.75	12.66	12.51	17	21	12	15	44.55	48.55	2.7	2.58	168.9	203.2	4.66	5.07	0.83	0.88	107	104	1.12	2.07	1.14	1.23	3.91	4.19	2.34	2.22
9.8	8.6	6.32	3.40	1.4	2.54	119.6	125.4	33.7	33.65	277.86	236.36	42.38	42.53	8.76	8.3	22	11	20	15	42.05	46	2.5	2.68	232.0	164.1	3.16	4.86	0.81	0.86	103	101	1.12	1.03	1.1	1.16	3.96	4.24	2.46	2.08
9.5	7.7	6.32	9.34	2.56	2.94	114.9	116.3	34.42	34.09	227.53	203.19	39.6	40.97	11.86	10.2	14	16	12	7	43.73	42.36	3.2	3.08	182.1	214.0	5.21	4.59	0.79	0.8	105	101	1.34	1.10	1.39	1.1	4.24	3.96	2.37	2.22
8.9	8.6	8.12	6.16	1.4	2.24	116.3	118.2	34.75	33.2	215.64	210.53	42.21	42.73	12.64	9.41	22	21	13	7	40.3	47.83	3.4	3.28	222.0	204.0	4.8	5.13	0.81	0.78	103	102	1.19	1.25	1.23	1.23	3.91	4.23	2.22	2.28
9.5	7.7	6.32	7.28	1.42	2.54	125.4	125.4	33.7	35.89	235.74	267.53	42.38	42.36	9.72	10.37	18	20	18	10	40.91	45.25	2.9	2.38	182.1	194.3	4.91	5.14	0.92	0.78	103	104	1.19	1.11	1.16	1.39	4.16	3.91	2.18	2.46
7.7	7	7.09	9.40	1.75	2.14	125.6	129.4	35.56	35.06	277.86	264.23	42.58	42.53	12.55	10.43	17	10	20	15	40.3	43.62	2.8	3.48	221.2	164.1	4.66	4.59	0.79	0.91	103	102	1.12	1.08	1.1	1.18	4.12	3.96	2.18	2.22
12.5	8.3	6.32	6.23	1.55	2.54	127.6	118.2	34.42	33.2	220.19	260.86	40.81	42.77	13.25	10.7	12	16	20	7	43.73	47.83	3.2	2.78	212.3	150.9	4.93	3.09	0.87	0.82	102	102	2.14	2.07	1.14	1.23	3.91	4.31	2.22	2.36
9.8	11.3	7.45	3.87	1.75	2.04	125.4	119.6	33.7	33.92	227.53	232.27	42.38	42.36	9.55	10.37	18	15	18	15	73.05	45.18	3.3	3.18	168.9	173.8	5.2	4.86	0.79	0.91	103	102	1.19	1.03	1.23	1.1	3.96	4.12	2.36	2.18
9.5	8.3	6.32	6.2	2.12	2.74	116.3	114.9	34.75	34.7	215.64	198.75	40.82	40.97	8.42	8.3	14	13	13	10	42.05	43.5	2.9	3.28	182.1	204.0	4.66	3.09	0.79	0.8	105	104	1.34	1.11	1.1	1.14	3.91	4.24	2.46	2.22
8.9	10.9	6.32	6.2	2.47	2.44	119.6	127.6	35.2	33.2	277.86	166.22	39.52	42.36	7.65	7.7	22	15	18	15	38.05	46.25	3.2	2.78	182.1	194.3	4.89	4.73	0.81	0.91	103	102	2.15	1.25	1.08	1.23	3.91	3.91	2.33	2.34
9.5	8.6	6.32	8	1.69	2.64	132.6	132.6	34.15	36.74	181.93	203.19	42.38	39.67	13.25	12.5	12	12	15	7	43.73	46	3.4	3.08	168.9	209.3	3.16	4.59	0.92	0.78	106	105	1.19	1.08	1.14	1.1	4.24	4.09	2.22	2.23
11.2	10.4	6.32	6.2	1.4	2.74	125.4	125.4	33.7	33.65	220.19	260.86	39.38	42.73	7.65	8.3	17	16	18	15	46.38	41.75	2.7	3.48	222.0	164.1	5.14	5.13	0.92	0.88	103	102	1.12	1.10	1.23	1.16	4.24	3.91	2.18	2.22
9.8	8.6	6.32	6.97	1.36	2.04	118.2	118.2	34.75	33.2	227.53	210.53	42.21	39.75	7.65	10.37	18	20	13	7	38.05	74.5	2.7	2.78	212.3	150.9	4.66	4.84	0.79	0.95	105	104	2.14	1.03	1.1	1.23	4.26	4.17	2.08	2.18
9.5	8.3	5.09	6.2	1.42	2.64	116.3	125.4	34.15	36.71	277.86	218.74	42.38	40.96	9.55	8.3	18	16	18	15	43.73	47.83	3.2	3.28	224.1	194.3	4.8	4.74	0.81	0.82	103	102	1.19	1.25	1.29	1.08	4.12	4.24	2.46	2.36
8.2	7.7	8.12	7.33	2.57	2.84	137.8	116.3	36.59	35.06	215.64	198.64	40.81	42.53	9.72	9.07	15	20	20	7	43.8	48.55	2.9	2.58	182.1	204.0	4.93	4.86	0.92	0.78	103	101	1.19	1.25	1.16	1.1	3.91	4.12	2.22	2.37
7.1	7	6.32	6.2	2.12	2.44	125.6	119.6	33.7	33.2	183.22	213.15	42.58	41.73	11.86	12.51	17	10	18	3	44.55	43.25	2.5	3.08	232.0	164.1	3.16	3.09	0.87	0.8	103	106	1.12	2.07	1.1	1.23	4.12	3.91	2.18	2.46
8.9	6.5	5.09	6.2	2.39	2.44	119.6	114.9	34.42	33.65	227.53	236.36	42.38	42.53	8.76	9.7	18	15	18	7	13.3	14.75	3.4	2.68	168.9	206.1	4.91	4.59	0.83	0.86	103	102	1.34	1.11	1.23	1.14	3.91	3.96	2.36	2.22
11.2	8.3	6.32	6.2	1.75	2.54	132.6	116.3	34.15	33.92	220.19	260.86	39.6	40.97	8.42	8.3	22	16	13	15	42.05	43.5	3.2	3.18	212.3	194.3	4.8	5.13	0.79	0.78	102	102	1.17	1.05	1.14	1.1	4.24	3.91	2.28	2.08
9.5	8.6	6.32	6.2	2.47	2.34	124.8	125.4	34.75	33.2	143.75	267.53	40.82	42.53	11.85	12.5	18	10	18	15	73.05	46	3.6	2.78	182.1	173.8	4.66	5.07	0.81	0.91	103	104	1.12	1.03	1.1	1.23	3.96	4.16	2.22	2.33
9.8	7.7	7.09	6.2	1.89	2.34	116.3	125.6	33.7	34.25	210.5	264.23	42.38	39.75	13.25	9.41	19	16	14	10	40.3	42.38	3.3	2.38	222.0	164.1	5.2	4.59	0.85	0.84	105	101	1.12	1.10	1.36	1.29	4.16	4.24	2.23	2.22
9.5	8.3	6.32	4.97	1.42	2.04	118.2	127.6	35.2	34.7	215.64	203.19	41.54	42.53	11.98	8.3	18	10	18	7	40.91	45.25	2.7	3.28	168.9	150.9	4.93	4.73	0.81	0.91	105	102	1.19	1.11	1.23	1.19	3.91	4.35	2.46	2.18
9.5	9.4	6.32	8	1.69	2.34	132.6	125.4	34.15	33.2	227.53	126.75	40.81	42.73	9.72	10.7	18	20	13	18	43.73	47.83	2.8	3.08	191.8	218.7	5.21	4.92	0.79	0.78	103	104	1.34	1.25	1.08	1.23	4.12	4.31	2.18	2.23
12.1	8.3	6.32	6.2	2.12	2.44	125.4	116.3	34.75	34.09	183.22	260.86	42.21	42.53	7.65	13.29	17	15	20	15	40.3	42.36	3.2	2.58	182.1	164.1	3.16	3.09	0.92	0.8	103	102	1.19	1.05	1.1	1.1	4.24	4.24	2.22	2.34
9.8	10	5.09	4.97	2.56	2.54	116.3	119.6	33.7	36.09	220.19	232.27	42.62	42.77	13.25	10.37	14	16	18	15	47.1	46.25	2.9	3.28	168.9	204.0	4.66	4.84	0.79	0.88	102	101	1.12	2.06	1.23	1.14	3.91	4.12	2.46	2.22
8.9	8.6	6.32	6.2	1.89	3.04	113.6	132.6	34.59	33.92	210.5	166.22	40.82	42.53	8.42	8.3	12	16	18	7	44.55	48.55	3.4	3.08	212.3	194.3	4.99	5.13	0.87	0.78	106	102	1.17	1.03	1.16	1.23	4.31	3.91	2.22	2.36
7.1	8.3	6.56	6.2	2.12	2.84	125.6	125.4	34.15	35.06	277.86	198.64	42.58	39.75	11.85	7.7	18	17	18	9	42.05	45.18	3.2	2.78	191.8	195.9	4.91	4.59	0.83	0.84	103	102	1.12	1.11	1.1	1.16	4.16	4.19	2.36	2.46
9.5	8.6	5.09	6.97	2.43	2.44	127.6	118.2	34.42	33.65	235.74	236.36	42.21	42.53	7.65	9.07	22	15	6	7	43.8	46	3.6	3.28	182.1	214.0	4.66	4.86	0.81	0.78	102	102	1.34	1.08	1.14	1.1	4.12	4.23	2.22	2.28
9.5	6.5	6.32	6.2	2.47	2.33	132.6	116.3	33.7	36.75	215.64	198.75	39.6	40.96	11.86	12.51	17	11	18	15	40.91	46	2.9	2.78	222.0	173.8	4.93	5.13	0.96	0.91	105	102	1.12	2.07	1.29	1.23	3.91	4.24	2.37	2.22
11.2	8.3	6.32	5.80	1.42	2.84	118.2	137.8	34.75	36.71	181.93	260.86	40.81	42.53	9.55	8.3	22	12	18	7	44.8	48.55	3.2	2.38	212.3	194.3	4.8	4.73	0.87	0.82	103	102	1.19	1.10	1.36	1.08	3.91	4.16	2.33	2.18
9.8	7.7	6.32	5.15	1.69	2.74	121.9	125.6	35.2	35.89	210.5	218.74	41.54	42.53	8.76	10.33	18	20	20	15	43.73	45.18	2.8	2.78	227.3	194.3	5.2	5.14	0.92	0.8	103	102	2.15	1.25	1.23	1.39	3.96	4.24	2.22	2.37
10.7	7	8.12	3.93	2.43	2.34	125.4	119.6	34.75	33.92	227.53	203.19	42.38	41.69	9.72	12.63	18	16	20	7	40.3	45.25	2.5	2.58	213.9	164.1	4.66	4.59	0.79	0.86	106	101	1.19	1.03	1.1	1.23	4.35	3.91	2.18	2.22
7.7	8.6	7.45	5.47	1.97	2.54	124.6	132.6	33.7	33.65	220.19	232.27	39.38	39.75	8.42	10.2	14	13	15	7	73.05	46.42	2.7	3.18	182.1	204.0	5.21	3.09	0.79	0.78	103	104	1.12	1.11	1.29	1.14	3.96	4.12	2.34	2.21
9.8	7	6.32	9.53	2.56	2.24	125.6	124.8	35.56	34.09	281.23	213.15	42.21	40.97	7.65	8.3	17	17	8	15	42.05	46.25	3.2	2.38	168.9	150.9	3.16	4.84	0.85	0.93	102	102	1.19	1.05	1.23	1.1	3.91	4.31	2.22	2.18
9.5	10	5.09	8.15	2.43	2.5038	131.2	116.3	34.42	36.71	215.64	236.36	42.58	42.73	12.55	9.41	13	15	20	3	47.1	43.5	2.9	3.08	212.3	150.9	4.93	4.59	0.92	0.84	105	104	2.15	1.08	1.14	1.23	4.24	4.35	2.23	2.22
8.9	11.7	6.32	6.59	1.42	1.256	137.8	118.2	34.75	36.72	210.5	260.86	40.82	39.2	11.76	10.37	22	16	18	15	44.55	45.18	3.4	3.28	222.0	194.3	4.99	5.13	0.89	0.78	103	102	1.12	2.06	1.1	1.29	3.91	4.16	2.46	2.08
9.5	8.3	6.32	5.80	1.55	3.04	118.2	132.6	33.7	36.71	183.22	213.15	40.81	39.2	7.65	12.5	22	12	18	10	43.73	14.75	3.2	3.08	182.1	164.5	4.8	4.86	0.83	0.91	105	102	1.34	1.05	1.16	1.1	4.16	3.91	2.22	2.37
11.6	8.6	7.09	4.97	2.47	2.74	119.6	125.4	37.24	33.2	215.75	210.53	39.52	40.96	13.25	8.3	18	13	14	7	40.3	41.75	3.3	2.78	224.1	194.3	4.91	3.09	0.81	0.8	103	101	1.12	1.03	1.23	1.23	3.91	4.24	2.21	2.22
8.2	10.9	6.56	6.2	1.55	1.74	121.9	116.3	34.15	36.09	227.53	126.75	41.58	42.36	12.55	10.2	14	10	18	15	40.91	47.83	2.9	3.08	168.9	164.1	4.93	5.19	0.92	0.88	103	105	1.19	1.25	1.08	1.16	3.96	3.91	2.22	2.36
8.9	11.7	6.32	6.44	1.69	2.74	125.4	113.6	35.2	35.06	215.64	198.64	42.21	39.75	8.42	9.07	17	20	20	15	73.05	46	3.2	2.58	212.3	204.0	4.66	5.13	0.87	0.91	105	102	1.13	1.11	1.14	1.14	4.12	4.12	2.46	2.46
12.9	8.6	6.32	4.97	1.89	2.54	124.6	125.6	36.39	34.09	210.5	260.86	42.62	41.73	7.65	12.51	18	16	20	1	44.55	47.35	2.7	2.38	182.5	164.1	5.2	3.09	0.79	0.78	102	102	1.19	2.07	1.23	1.23	3.96	3.91	2.18	2.34
10.6	8.3	6.32	6.2	1.75	2.44	125.6	127.6	34.42	36.71	277.86	236.36	42.58	42.73	9.72	10.33	18	15	18	7	42.05	43.5	3.4	2.58	182.1	164.1	3.16	4.59	0.92	0.78	102	101	2.14	1.08	1.1	1.1	3.91	4.09	2.36	2.18
9.8	10	6.32	6.2	2.43	2.14	132.6	132.6	34.15	33.65	235.74	203.19	40.81	39.67	11.86	8.3	18	16	18	15	43.8	42.36	2.7	3.08	222.0	194.3	4.66	4.82	0.81	0.86	103	102	1.19	1.10	1.39	1.08	4.23	3.91	2.28	2.22
9.5	11.3	6.32	6.2	2.39	2.54	118.2	118.2	34.59	36.99	220.19	166.22	40.82	42.77	7.65	8.18	17	15	23	9	40.3	45.18	3.2	3.28	212.3	209.3	4.81	4.73	0.79	0.82	105	102	1.34	1.03	1.23	1.23	3.96	4.16	2.22	2.33
11.6	5.9	6.32	8	2.12	2.94	119.6	121.9	35.2	34.25	230.15	198.75	41.58	42.36	13.25	13.31	19	16	18	10	40.91	45.25	2.5	3.28	168.9	173.8	4.93	4.59	0.94	0.8	103	104	1.12	1.11	1.14	1.16	4.12	4.24	2.33	2.28
9.5	7.7	6.32	7.33	1.55	2.24	127.6	125.4	37.21	34.7	249.27	264.23	42.38	40.96	11.85	8.3	22	15	20	7	43.73	47.83	3.4	3.48	182.1	150.9	4.8	3.09	0.79	0.84	103	102	1.19	1.25	1.1	1.1	3.96	4.31	2.34	2.21
8.9	9.4	6.56	6.2	2.43	2.54	116.3	124.6	34.15	35.89	277.86	260.86	42.21	40.97	7.65	12.5	12	12	18	15	44.55	74.5	2.5	3.08	191.8	203.2	3.16	5.13	0.87	0.88	103	102	2.15	1.05	1.23	1.23	3.91	4.24	2.22	2.22
11.2	10	8.12	4.97	1.75	2.14	125.4	125.6	35.56	33.65	215.64	210.53	41.58	39.53	7.65	9.41	23	10	20	12	41.8	41.75	3.2	2.78	227.3	164.1	4.66	5.14	0.85	0.84	105	104	1.17	2.06	1.16	1.14	4.16	4.12	2.46	2.21
9.5	8.3	6.32	6.2	1.69	2.54	124.6	131.2	34.42	36.71	183.22	236.36	39.05	39.75	8.76	13.2	15	21	15	15	43.73	43.5	2.5	2.68	182.1	204.0	4.8	4.84	0.79	0.78	103	102	2.15	1.25	1.1	1.36	4.35	3.91	2.46	2.36
12.9	8.6	6.32	6.2	1.42	2.04	121.9	137.8	35.2	33.2	215.75	218.74	42.58	42.73	11.85	8.3	18	20	18	3	44.55	46	2.5	3.18	212.3	150.9	5.14	5.07	0.81	0.91	106	101	2.14	1.03	1.23	1.23	3.91	4.09	2.18	2.22
9.8	11.3	6.32	6.97	2.57	2.74	124.8	118.2	34.15	33.92	143.75	126.75	40.81	42.36	9.72	12.51	18	16	8	12	42.05	46.25	2.5	2.38	191.8	164.1	4.66	5.13	0.92	0.84	103	102	1.12	2.07	1.14	1.18	4.24	4.23	2.22	2.18
8.3	8.3	5.09	6.44	2.12	2.44	118.2	119.6	36.59	36.99	277.86	203.19	42.62	42.53	7.65	8.3	17	16	20	15	42.17	45.18	3.2	3.28	222.0	194.3	4.93	3.09	0.79	0.8	105	102	1.19	1.08	1.36	1.08	3.91	4.31	2.08	2.46
7.7	9.5	6.32	4.42	1.75	2.64	119.6	121.9	33.7	34.09	215.64	260.86	42.21	42.36	11.85	9.07	22	13	18	10	43.73	43.62	2.5	2.58	182.1	206.1	4.91	4.59	0.83	0.82	103	104	1.34	1.11	1.1	1.23	4.31	4.24	2.37	2.37
9.5	6.5	6.32	7.45	2.43	2.74	125.6	125.4	34.59	36.74	235.74	198.75	40.82	41.73	9.55	13.2	17	11	20	15	13.3	47.83	2.9	3.08	168.9	150.9	4.66	4.86	0.79	0.86	102	102	1.19	1.25	1.23	1.16	3.91	4.35	2.36	2.22
9.8	10	6.32	4.42	1.55	2.04	116.3	124.6	34.15	36.75	210.5	198.64	42.21	42.53	11.86	10.2	22	16	18	12	40.3	48.55	2.8	3.48	224.1	150.9	4.93	4.59	0.92	0.95	107	102	1.19	2.07	1.08	1.1	3.96	3.91	2.33	2.34
8.2	8.3	6.32	5.09	1.69	2.64	125.4	125.6	35.2	33.92	281.23	236.36	39.52	42.77	7.65	8.3	13	15	18	12	40.91	14.75	3.2	2.58	212.3	173.8	4.8	5.14	0.81	0.88	103	102	1.13	1.11	1.14	1.23	4.24	4.12	2.22	2.08
11.6	8.3	7.45	4.42	2.54	2.84	124.6	132.6	33.7	36.09	220.19	264.23	42.58	40.96	8.42	10.2	18	16	20	12	40.3	43.5	3.6	2.78	168.9	164.1	3.16	5.13	0.89	0.78	103	104	1.19	2.06	1.23	1.29	4.12	3.91	2.21	2.21
12.5	7	6.32	4.42	2.43	2.44	132.6	118.2	34.42	35.06	277.86	210.53	39.6	42.53	11.98	12.5	18	12	20	15	73.05	45.25	2.9	3.08	232.0	194.3	5.2	4.73	0.85	0.78	105	101	1.12	1.03	1.1	1.08	4.09	4.24	2.22	2.18
9.5	10	6.32	4.85	2.57	2.44	121.9	119.6	37.24	36.74	183.22	260.86	40.81	40.97	9.72	13.29	18	20	20	1	42.05	46	3.3	3.28	182.1	204.0	5.26	4.84	0.83	0.8	102	102	2.14	2.07	1.29	1.23	3.96	4.17	2.28	2.28
9.8	5.9	6.32	6.44	2.39	2.54	118.2	127.6	34.15	34.09	210.5	166.22	42.38	42.36	7.65	8.3	18	15	20	15	44.55	45.18	3.2	2.78	212.3	214.0	4.66	4.92	0.92	0.91	103	102	1.34	1.11	1.1	1.14	3.91	3.91	2.18	2.22
10.7	8.6	6.56	8	1.75	2.34	119.6	116.3	33.7	33.92	227.53	213.15	41.58	42.53	12.55	9.41	12	16	18	12	43.73	41.75	3.4	3.08	222.0	150.9	4.99	4.86	0.79	0.82	105	101	1.17	1.25	1.14	1.39	4.16	4.16	2.46	2.46
8.2	8.3	6.32	6.2	1.55	2.34	116.3	125.4	36.39	33.65	215.75	203.19	42.38	41.69	13.25	10.37	19	10	14	10	43.8	43.25	2.9	2.38	236.7	194.3	4.66	4.59	0.79	0.91	103	106	2.14	1.10	1.16	1.23	3.96	4.24	2.34	2.36
9.5	10.9	6.46	6.2	1.89	2.04	121.6	124.6	35.2	35.89	215.64	198.75	40.82	42.73	8.76	8.3	17	10	18	3	46.38	42.36	3.2	3.18	182.1	164.1	3.16	4.52	0.81	0.84	102	102	1.19	2.07	1.23	1.36	4.17	4.31	2.37	2.33
9.8	7.1	6.32	6.2	1.69	2.34	125.4	121.9	35.56	36.09	277.86	260.86	42.38	39.75	12.64	12.51	22	12	18	15	44.8	46.25	2.7	3.08	168.9	204.0	5.21	3.09	0.92	0.86	103	102	1.12	1.03	1.1	1.1	3.96	3.91	2.36	2.37
9.5	7.7	8.12	4.97	1.55	2.44	124.6	124.8	33.7	33.2	210.5	264.23	39.6	39.67	7.65	9.07	17	15	15	12	40.3	43.5	2.5	2.68	212.3	150.9	4.66	4.84	0.96	0.78	103	102	1.19	1.11	1.23	1.23	4.24	4.12	2.22	2.18
8.9	8.3	7.09	6.2	2.43	2.54	137.8	118.2	34.42	36.74	230.15	218.74	42.38	42.77	13.25	10.37	12	20	20	10	46.38	74.5	2.5	2.78	182.5	164.1	4.8	5.13	0.92	0.8	103	104	2.15	1.05	1.39	1.08	3.91	4.35	2.33	2.22
12.1	6.5	6.46	6.2	2.47	3.04	125.6	119.6	34.59	35.06	284.53	210.53	42.58	42.36	13.25	8.3	13	16	20	15	42.05	47.83	3.2	3.08	168.9	164.5	5.2	5.14	0.79	0.82	103	102	1.12	2.07	1.14	1.16	4.31	4.31	2.18	2.36
12.9	9.5	7.45	6.2	2.43	2.84	118.2	125.6	34.75	34.7	220.19	198.64	42.38	42.53	11.86	12.5	18	16	20	15	40.3	45.18	2.7	3.28	182.1	194.3	4.66	4.84	0.79	0.88	103	105	1.34	2.07	1.1	1.23	3.96	4.23	2.21	2.34
9.8	8.6	6.46	6.2	2.12	2.44	119.6	116.3	33.7	34.25	235.74	260.86	42.62	41.73	7.65	10.37	18	21	18	15	43.73	46	2.9	2.58	222.0	164.1	4.93	4.59	0.83	0.91	103	102	1.13	1.25	1.23	1.1	4.16	4.24	2.23	2.23
12.5	8.3	6.32	7.33	1.42	2.33	116.3	125.4	34.15	33.92	210.5	236.36	42.38	40.96	9.72	12.63	15	13	14	15	44.55	48.55	2.7	2.58	212.3	173.8	4.91	4.86	0.81	0.82	102	101	1.12	1.03	1.08	1.16	4.24	4.31	2.22	2.46
9.5	10.9	5.09	6.2	2.21	2.84	121.9	124.6	35.2	36.99	277.86	126.75	39.6	40.97	7.65	12.41	22	16	18	12	73.05	41.75	3.2	2.38	221.2	150.9	4.93	4.73	0.92	0.78	105	102	1.19	1.11	1.16	1.23	4.12	4.12	2.37	2.23
8.2	8.6	6.32	4.06	1.75	2.74	125.4	118.2	37.49	36.74	215.75	203.19	42.38	42.73	9.55	8.3	18	16	20	9	47.1	45.25	2.8	3.08	224.1	194.3	3.16	3.09	0.85	0.84	103	101	1.19	1.08	1.1	1.14	3.91	3.91	2.28	2.18
12.1	7	6.46	6.54	1.69	2.34	124.6	119.6	33.7	35.06	227.53	166.22	40.81	42.36	12.26	10.2	18	15	20	12	46.38	43.5	3.4	2.78	222.0	164.1	4.66	4.59	0.89	0.78	105	102	1.34	1.10	1.14	1.1	3.96	4.16	2.22	2.22
9.8	8.6	6.46	7.33	2.56	1.163	131.2	137.8	36.59	34.7	215.64	198.75	42.38	42.36	11.85	9.07	22	20	12	15	42.05	47.83	2.9	3.28	182.1	204.0	5.2	4.74	0.92	0.8	103	102	1.12	1.05	1.23	1.23	4.23	4.24	2.46	2.21
9.5	8.3	6.46	6.2	2.43	1.182	132.6	116.3	34.15	33.2	210.5	260.86	42.38	42.53	7.65	12.5	18	15	20	10	40.3	43.25	3.4	2.68	212.3	209.3	4.89	4.92	0.83	0.91	103	101	1.17	1.11	1.23	1.29	4.09	4.12	2.18	2.28
12.9	10.4	6.46	6.2	1.89	1.326	118.2	124.6	35.2	34.25	183.22	236.36	41.54	39.75	8.42	8.3	17	20	20	15	42.17	45.18	3.6	3.08	191.8	194.3	3.16	3.09	0.81	0.84	102	102	1.19	1.03	1.23	1.08	4.24	4.19	2.22	2.18
9.5	7.1	6.46	6.2	1.55	1.254	119.6	121.9	33.7	34.09	277.86	236.36	39.6	39.2	9.72	9.41	18	11	20	12	43.73	14.75	3.2	2.48	232.0	164.1	4.66	5.14	0.79	0.78	106	104	1.12	2.07	1.1	1.23	3.96	4.24	2.36	2.36
8.9	6.5	6.32	6.44	1.78	1.163	116.3	125.4	37.24	33.2	230.15	166.22	40.82	42.36	7.65	10.37	17	16	12	15	43.8	46	3.3	3.28	168.9	218.7	4.8	4.59	0.92	0.91	103	102	1.34	1.25	1.29	1.19	3.91	3.91	2.34	2.37
8.2	8.3	6.46	6.2	2.39	1.136	121.9	124.8	34.59	33.92	281.23	218.74	42.58	42.73	7.05	9.07	18	16	12	3	44.55	48.55	3.4	3.18	168.9	194.3	5.14	4.86	0.87	0.86	103	101	2.14	2.06	1.18	1.1	4.16	4.24	2.22	2.22
8.3	7	8.12	6.34	2.63	1.256	124.6	118.2	34.15	33.65	210.5	198.64	39.05	40.97	11.86	8.3	12	16	12	12	40.3	47.83	3.2	3.08	182.1	150.9	4.93	4.84	0.79	0.91	102	102	1.19	1.11	1.1	1.23	4.12	4.12	2.21	2.46
10.7	8.3	6.46	6.2	1.69	1.276	125.4	132	33.7	34.7	220.19	203.19	39.05	42.36	9.72	12.51	14	16	15	10	42.05	43.5	2.5	2.38	212.3	204.0	5.2	3.09	0.85	0.78	103	104	1.12	1.10	1.23	1.14	4.24	4.26	2.36	2.34
8.9	11.7	6.46	8	2.12	1.326	125.6	129	35.2	33.2	215.75	210.53	40.81	39.75	8.42	12.41	18	10	20	15	73.05	41.75	2.5	2.58	227.3	164.1	4.66	4.73	0.81	0.82	103	102	2.15	1.03	1.14	1.39	3.96	4.12	2.46	2.36
9.5	8.6	6.46	6.97	1.89	1.182	121.6	112.8	37.49	36.74	277.86	260.86	42.21	39.2	7.05	12.5	19	17	20	15	40.91	42.36	2.9	2.68	222.0	173.8	4.96	5.07	0.83	0.8	105	101	1.19	1.08	1.16	1.23	3.96	3.91	2.18	2.33
9.8	7.7	8.12	6.34	2.21	1.219	118.2	117.5	35.56	34.09	215.64	236.36	39.6	39.67	8.76	8.3	22	15	18	15	43.73	45.18	3.2	3.28	213.9	150.9	4.66	4.59	0.92	0.95	103	102	1.17	1.10	1.23	1.36	3.91	4.09	2.22	2.22
11.6	9.5	6.32	7.33	1.55	1.254	119.6	125.4	33.7	33.65	210.5	203.19	41.58	42.53	10.05	9.07	13	20	8	12	46.38	47.83	3.2	3.08	168.9	206.1	4.91	5.14	0.79	0.78	103	102	1.19	1.03	1.23	1.14	4.19	4.24	2.21	2.18
9.5	5.9	5.09	6.34	1.42	1.246	137.8	122.3	35.2	33.2	235.74	193.5	42.58	41.73	7.05	13.31	18	15	16	9	44.8	42.38	2.7	3.18	212.3	164.1	4.8	3.09	0.94	0.8	105	102	1.34	2.07	1.1	1.23	4.31	4.31	2.33	2.21
12.1	11.3	6.46	6.2	2.47	1.256	116.3	121.9	37.49	34.7	249.27	198.64	39.52	42.53	11.85	10.37	18	10	20	11	40.3	46	2.6	3.28	222.0	164.5	4.93	4.86	0.87	0.95	103	102	1.12	1.25	1.08	1.16	3.91	4.12	2.22	2.08
8.9	8.3	6.46	4.97	2.43	1.312	124.6	116.3	36.59	36.99	183.22	210.53	42.62	42.73	9.55	8.18	14	11	20	15	44.55	45.25	2.8	3.08	182.1	194.3	4.99	4.59	0.79	0.78	102	105	1.19	1.03	1.14	1.1	4.24	3.91	2.18	2.46
9.5	10	6.46	6.2	1.69	1.378	121.9	125.6	35.2	35.06	277.86	260.86	42.21	42.36	9.72	10.43	17	16	12	12	42.05	43.5	3.2	2.38	212.3	204.0	4.66	4.73	0.83	0.8	103	102	1.13	1.05	1.1	1.23	3.96	4.19	2.37	2.37
7.7	7	6.46	6.34	2.57	1.182	125.4	137.8	34.15	33.2	210.5	193.5	40.81	40.96	8.42	8.3	17	16	12	9	73.05	47.83	2.9	2.38	224.1	214.0	5.2	4.82	0.81	0.91	103	102	1.12	1.11	1.19	1.08	4.12	4.24	2.22	2.22
10.7	8.3	6.56	6.34	2.39	1.196	124.8	127.6	37.24	34.7	227.53	218.74	40.82	40.97	11.86	9.07	18	13	14	12	46.38	47.35	2.7	2.78	213.9	150.9	4.91	4.73	0.92	0.86	105	104	1.12	2.06	1.23	1.14	3.96	4.16	2.08	2.28
9.8	8.6	5.09	6.34	2.43	1.219	118.2	118.2	34.42	36.99	143.75	203.19	39.38	39.75	7.65	9.41	23	20	20	12	41.8	45.18	3.2	3.08	182.1	194.3	4.93	3.09	0.92	0.82	103	102	2.14	1.05	1.1	1.23	4.35	4.35	2.22	2.18
9.5	10	5.09	6.34	1.89	1.254	119.6	125.4	37.49	36.09	220.19	210.53	39.6	42.77	9.55	12.51	22	16	15	15	43.8	48.55	2.9	3.08	222.0	164.1	3.16	4.84	0.79	0.78	103	104	1.19	1.10	1.16	1.29	3.91	3.91	2.28	2.36
12.1	11.7	6.46	6.34	2.12	1.246	125.6	124.6	35.2	34.7	215.64	193.5	42.58	41.69	8.76	8.3	18	16	20	15	46.38	41.75	3.4	2.58	212.3	173.8	4.66	3.09	0.89	0.91	103	102	1.34	1.03	1.14	1.1	4.24	4.31	2.46	2.34
9.8	8.3	6.46	6.2	1.75	1.256	124.6	124.8	34.15	33.65	210.5	260.86	42.21	42.36	9.72	9.07	18	20	12	10	42.05	74.5	3.2	2.48	191.8	194.3	4.66	5.19	0.83	0.88	105	101	2.15	2.07	1.23	1.23	3.96	4.12	2.22	2.21
8.9	8.6	6.46	6.34	2.43	1.326	116.3	121.9	34.75	36.74	277.86	198.64	42.38	42.73	9.78	10.37	12	16	12	3	13.3	46	3.3	2.68	168.9	150.9	4.89	4.59	0.81	0.8	102	102	1.12	1.25	1.1	1.16	3.91	4.23	2.36	2.22
8.2	5.9	7.45	8	1.76	1.182	118.2	125.6	34.59	33.92	215.75	166.22	42.21	42.36	10.05	13.9	19	15	15	12	46.38	43.5	2.9	3.08	182.1	209.3	4.8	4.86	0.79	0.78	103	102	1.19	2.06	1.18	1.1	4.12	4.31	2.18	2.37
9.5	8.3	6.46	6.34	1.69	1.196	132.6	122.3	34.42	36.99	230.15	193.5	41.58	42.53	9.72	8.3	14	16	20	12	44.55	43.62	3.2	2.78	222.0	194.3	5.2	4.84	0.87	0.78	103	102	1.17	2.05	1.23	1.23	3.96	3.91	2.22	2.46
12.5	9.5	6.46	6.34	1.97	1.276	125.4	118.2	35.56	34.7	235.74	203.19	42.38	39.75	7.65	10.2	15	15	12	10	40.3	48.55	3.6	2.58	168.9	164.1	3.16	4.59	0.79	0.8	102	102	2.14	1.11	1.08	1.29	3.91	4.24	2.34	2.18
9.5	7	6.32	6.34	1.75	1.163	118.2	127.6	34.15	33.65	210.5	260.86	42.62	40.97	7.05	9.07	17	16	20	15	73.05	45.18	2.7	3.08	212.3	204.0	4.93	4.73	0.85	0.86	107	101	1.34	1.03	1.23	1.14	4.09	4.17	2.23	2.33
12.1	8.6	6.46	8	2.43	1.254	121.9	125.4	33.7	34.25	281.23	210.53	40.81	40.96	11.85	12.5	17	10	20	12	42.05	45.25	3.4	2.78	221.2	206.1	5.14	4.74	0.83	0.84	103	102	1.19	1.10	1.1	1.23	3.91	4.26	2.22	2.23
9.8	8.3	5.09	6.2	1.75	1.246	118.2	119.6	35.2	34.09	277.86	198.64	42.38	42.36	7.65	8.3	22	12	14	15	47.1	42.36	3.2	3.28	182.1	194.3	4.93	3.09	0.92	0.8	103	102	1.12	1.08	1.14	1.1	4.16	4.12	2.18	2.22
11.6	11.7	6.46	4.97	2.12	1.219	119.6	132.6	34.59	33.92	227.53	218.74	40.82	39.53	9.72	10.2	18	16	12	15	40.91	47.83	2.8	3.08	232.0	150.9	4.66	4.84	0.81	0.82	103	104	1.19	1.05	1.23	1.16	3.91	3.91	2.36	2.36
9.8	8.3	6.32	6.34	1.42	1.248	121.6	124.6	37.49	35.06	220.19	260.86	42.21	40.97	7.65	13.2	18	17	20	1	73.05	14.75	2.5	3.18	222.0	204.0	5.2	5.07	0.96	0.8	105	105	2.14	1.25	1.1	1.23	3.96	4.24	2.37	2.23
9.5	7.7	6.46	6.34	2.56	1.182	116.3	131.2	34.15	33.65	210.5	203.19	42.38	39.67	8.42	8.3	14	20	20	12	40.3	43.5	3.2	2.78	212.3	164.1	5.21	4.86	0.79	0.78	103	102	1.17	2.07	1.16	1.18	4.24	4.12	2.46	2.28
8.9	7	7.09	6.34	1.76	1.196	124.6	125.6	34.42	33.2	215.64	210.53	41.54	42.53	11.86	10.37	18	11	12	15	43.73	46	2.7	3.08	182.1	194.3	4.66	3.09	0.87	0.8	106	101	1.19	1.03	1.23	1.29	3.96	4.16	2.22	2.46
8.2	7.1	8.12	6.34	1.69	1.256	137.8	137.8	35.2	34.7	183.22	198.64	42.58	42.36	9.05	12.5	18	16	12	3	44.55	41.75	3.2	3.48	168.9	218.7	3.16	4.59	0.87	0.91	103	102	1.19	1.10	1.1	1.23	3.91	4.31	2.08	2.18
7.7	9.5	6.46	6.44	1.75	1.163	125.4	119.6	36.59	34.09	277.86	260.86	39.6	42.73	7.65	12.51	22	16	15	10	43.8	45.18	2.9	2.58	191.8	204.0	4.93	4.73	0.81	0.78	102	102	1.12	1.11	1.29	1.1	4.31	3.91	2.33	2.22
9.5	7.7	7.02	4.97	2.39	1.254	118.2	125.4	37.24	36.99	215.75	164.93	39.52	42.53	11.85	13.9	12	12	20	12	42.05	46.42	3.2	3.28	222.0	164.1	3.16	4.92	0.92	0.86	103	101	1.34	2.06	1.23	1.08	3.91	4.24	2.22	2.34
9.8	8.3	7.02	4.97	1.97	1.246	125.6	122.3	34.15	33.65	284.53	203.19	42.62	40.96	8.76	9.41	13	15	12	15	46.38	47.83	2.6	3.08	212.3	150.9	4.8	4.59	0.83	0.78	102	106	2.15	2.07	1.1	1.23	4.12	4.09	2.18	2.33
8.9	8.6	5.09	6.34	1.78	1.326	121.9	127.6	35.2	33.92	210.5	210.53	42.21	42.36	7.65	12.63	22	15	20	9	44.97	48.55	3.3	2.68	227.3	173.8	4.66	4.89	0.87	0.78	103	102	1.13	1.11	1.19	1.39	4.19	4.12	2.23	2.22
9.5	10.4	7.02	6.34	2.57	1.219	122.3	118.2	34.42	34.7	230.15	260.86	42.38	42.53	10.05	8.3	17	16	15	15	45.9	46.25	2.5	2.38	182.1	194.3	5.2	3.09	0.79	0.8	103	104	1.34	1.08	1.23	1.1	4.24	4.35	2.34	2.28
10.6	8.3	8.12	6.34	2.54	1.182	124.6	131.2	37.49	36.09	215.64	198.64	41.58	40.97	12.64	12.41	18	21	20	12	73.05	43.5	3.4	3.08	168.9	206.1	4.91	5.14	0.92	0.91	102	101	1.17	1.11	1.08	1.23	3.96	3.91	2.22	2.08
9.5	10.9	7.02	7.33	1.89	1.196	116.3	124.6	33.7	36.74	277.86	166.22	40.81	39.75	9.72	10.2	17	20	12	12	43.73	14.75	2.7	2.58	212.3	214.0	4.81	4.73	0.81	0.91	103	102	1.12	1.25	1.1	1.16	4.23	4.23	2.36	2.36
11.2	7	7.02	6.34	1.69	1.163	132.6	121.6	34.15	33.65	220.19	210.53	40.82	42.53	7.65	12.91	12	16	20	15	13.3	47.83	2.7	3.08	222.0	164.1	4.93	4.86	0.89	0.78	105	104	1.13	1.03	1.23	1.14	3.91	4.31	2.46	2.18
9.8	8.3	7.02	6.34	1.76	1.216	121.9	125.6	37.22	34.7	227.53	203.19	42.58	42.73	7.05	12.5	13	16	12	15	44.55	46	2.5	2.78	182.1	194.3	3.16	4.73	0.79	0.8	103	102	1.34	1.10	1.14	1.23	3.96	4.12	2.28	2.46
9.5	5.9	6.32	6.2	2.43	1.254	125.4	125.6	34.59	33.92	235.74	126.75	42.21	42.36	8.42	12.51	22	10	20	12	73.05	41.75	3.2	3.08	224.1	204.0	4.66	4.59	0.92	0.91	102	102	2.14	1.05	1.29	1.1	4.31	4.19	2.22	2.22
9.8	6.5	7.02	6.34	1.42	1.246	118.2	113.6	33.7	36.99	210.5	193.5	42.21	42.53	11.86	8.3	18	17	12	10	40.3	74.5	2.8	2.48	212.3	195.9	5.2	4.92	0.87	0.86	103	102	1.19	1.03	1.23	1.18	4.12	3.91	2.18	2.37
7.7	9.4	7.02	4.97	1.75	1.378	125.6	125.4	37.21	33.2	284.53	198.64	42.38	42.77	7.65	9.41	18	12	8	10	43.8	43.5	2.7	3.18	191.8	150.9	5.14	4.84	0.83	0.82	105	101	1.19	1.03	1.1	1.29	4.24	4.09	2.37	2.33
9.5	10	5.09	6.34	2.47	1.256	131.2	119.6	34.15	33.65	277.86	210.53	39.6	40.96	9.68	13.9	22	13	12	12	40.91	48.55	2.5	2.38	182.1	173.8	4.66	3.09	0.81	0.78	103	104	1.12	2.06	1.16	1.1	3.91	4.12	2.22	2.34
8.9	7	6.32	6.2	2.56	1.182	124.6	121.6	34.42	36.72	183.22	166.22	39.05	42.53	11.98	12.63	18	15	20	12	46.38	42.36	3.2	3.28	168.9	150.9	4.8	5.07	0.79	0.8	102	102	2.15	1.10	1.23	1.23	4.09	4.24	2.21	2.28
8.2	8.3	7.02	6.34	2.39	1.196	121.6	113.6	33.7	34.09	215.75	203.19	42.21	40.97	9.55	13.2	18	15	20	15	43.73	74.5	3.4	2.58	236.7	194.3	4.99	4.92	0.83	0.84	103	102	2.14	1.25	1.08	1.23	3.91	4.31	2.18	2.18
9.8	9.5	7.02	6.97	2.21	1.163	116.3	136	34.75	33.2	215.64	193.5	42.58	41.73	7.65	10.37	15	20	15	3	41.8	41.75	3.2	2.58	182.1	164.1	3.16	3.09	0.94	0.8	103	104	1.34	2.07	1.1	1.14	4.17	3.91	2.22	2.22
8.2	8.6	7.02	8	1.69	1.219	119.6	142	37.24	36.71	220.19	260.86	40.82	42.53	8.76	13.31	12	16	12	15	44.55	45.18	2.9	2.38	222.0	194.3	4.91	4.59	0.87	0.78	103	105	2.14	1.03	1.23	1.16	4.24	4.23	2.08	2.21
11.2	7	8.12	6.34	1.75	1.254	121.9	140	34.15	33.65	210.5	218.74	42.21	39.75	9.72	8.3	17	16	23	15	47.1	46	3.3	3.08	212.3	204.0	5.2	4.86	0.83	0.91	103	102	1.19	1.11	1.14	1.1	4.12	4.12	2.37	2.46
12.9	8.3	7.02	6.9	2.43	1.246	125.4	136	34.75	33.92	277.86	198.64	39.6	42.36	11.85	12.63	17	12	20	12	44.8	45.25	3.4	2.68	213.9	194.3	4.66	4.84	0.92	0.78	106	101	1.12	1.08	1.16	1.23	3.91	4.16	2.22	2.36
9.5	7.7	7.02	6.9	1.89	1.312	118.2	155	35.56	33.2	281.23	164.93	39.05	42.53	7.65	13.29	14	16	20	18	40.3	43.5	3.6	2.58	232.0	164.5	4.93	4.82	0.81	0.86	103	102	1.19	2.05	1.23	1.24	3.96	4.12	2.36	2.23
9.8	11.3	7.02	4.97	2.57	1.326	125.6	136	34.42	34.25	227.53	193.5	39.52	42.73	9.55	12.51	23	16	12	3	40.93	47.83	3.2	2.38	191.8	206.1	5.2	4.73	0.79	0.82	103	102	1.13	1.25	1.1	1.23	3.91	3.91	2.46	2.37
12.1	11.7	6.56	6.9	1.76	1.182	122.3	175	36.59	36.74	143.75	210.53	42.38	40.96	8.42	8.3	22	20	14	12	43.8	46.42	2.7	3.08	212.3	194.3	4.8	4.59	0.85	0.8	105	102	1.19	1.10	1.39	1.39	4.16	4.31	2.34	2.22
8.3	7.1	6.32	8	2.21	1.196	124.6	142	34.15	33.65	230.15	203.19	41.58	42.53	11.86	9.41	19	10	12	12	43.73	47.35	3.4	3.28	212.3	209.3	5.21	3.09	0.96	0.95	103	104	1.34	1.03	1.23	1.1	4.24	4.35	2.18	2.18
9.8	7.7	5.09	6.9	1.75	1.163	119.6	169	37.22	34.25	277.86	264.23	42.38	40.97	9.68	9.07	18	11	20	15	13.3	74.5	2.9	3.08	182.1	150.9	4.66	4.59	0.87	0.86	105	102	2.14	1.11	1.36	1.23	4.35	3.91	2.22	2.08
12.9	8.3	7.02	6.9	2.39	1.219	116.3	132.6	34.59	35.06	210.5	198.64	42.58	39.67	7.65	10.37	22	20	12	10	44.55	45.18	3.2	2.78	222.0	164.1	3.16	5.07	0.79	0.91	103	102	1.12	2.06	1.1	1.16	4.31	4.31	2.33	2.34
11.6	10	7.02	6.9	2.43	1.246	132.6	125.4	36.39	33.92	235.74	193.5	42.21	42.53	7.53	10.2	18	15	20	15	73.05	14.75	2.9	3.18	168.9	173.8	4.91	5.19	0.89	0.78	102	101	1.17	1.08	1.23	1.19	4.24	4.12	2.28	2.46
8.2	8.3	6.32	6.2	1.69	1.254	137.8	118.2	34.42	36.09	215.64	166.22	40.81	39.53	12.66	12.5	14	16	12	4	46.38	46	2.8	3.28	168.9	194.3	4.66	3.09	0.81	0.78	103	104	1.19	1.11	1.14	1.23	4.12	4.24	2.21	2.21
9.5	11.7	7.02	6.9	1.42	1.256	125.4	125.4	33.7	33.65	220.19	198.75	40.82	42.36	7.65	12.5	18	15	12	9	40.91	74.5	3.3	3.48	212.3	194.3	5.2	4.86	0.92	0.84	103	102	2.15	1.10	1.08	1.1	3.91	4.17	2.22	2.22
10.7	6.5	5.09	6.9	2.47	1.216	125.6	116.3	37.21	36.72	183.22	210.53	39.6	42.53	11.85	8.3	17	10	20	12	40.3	41.75	2.7	3.08	182.5	204.0	4.93	4.73	0.79	0.91	103	102	1.19	1.03	1.23	1.23	4.19	3.91	2.21	2.36
8.2	10.4	7.02	4.97	1.75	1.182	121.9	119.6	35.2	34.09	215.75	198.64	42.62	40.96	8.76	9.07	13	11	8	15	42.17	45.25	3.2	2.58	212.3	194.3	3.16	4.84	0.85	0.88	103	101	1.34	1.25	1.16	1.34	4.23	4.12	2.36	2.18
7.7	11.7	7.02	6.2	1.89	1.196	124.6	114.9	35.56	35.89	227.53	193.5	41.54	42.73	12.55	8.3	22	20	20	12	43.73	42.36	2.7	3.28	182.1	150.9	5.26	3.09	0.83	0.82	102	102	1.12	2.07	1.1	1.23	4.24	4.24	2.22	2.28
11.2	8.6	7.02	6.9	2.12	1.378	131.2	116.3	36.59	33.92	210.5	260.86	42.21	42.53	7.65	13.9	17	16	15	12	47.1	47.83	2.5	2.78	222.0	194.3	5.2	4.59	0.92	0.8	103	104	1.34	1.05	1.23	1.23	4.16	4.16	2.18	2.33
7.1	7	6.46	6.9	1.75	1.163	122.3	125.4	34.15	33.2	249.27	218.74	42.58	39.75	11.86	12.41	12	16	12	10	42.05	45.18	3.4	3.08	182.1	164.1	3.16	5.14	0.94	0.91	103	106	1.19	1.25	1.39	1.16	4.24	4.31	2.46	2.28
12.5	11.7	4.42	6.9	2.43	1.246	116.3	125.6	34.42	36.71	281.23	203.19	42.21	40.97	7.65	10.37	18	20	20	15	44.55	43.25	3.2	2.78	182.1	194.3	4.66	4.74	0.81	0.86	105	102	2.14	1.08	1.18	1.1	3.91	3.91	2.37	2.22
9.8	7.1	6.32	8	1.58	1.219	125.6	127.6	35.2	34.7	215.64	213.15	42.38	42.53	8.42	13.2	18	16	20	4	43.8	46	3.6	2.68	212.3	194.3	4.89	5.13	0.89	0.78	106	101	1.19	1.03	1.23	1.14	4.12	4.12	2.22	2.46
8.9	10.9	4.42	6.9	1.76	1.254	125.4	125.4	37.22	35.06	230.15	232.27	39.6	41.69	12.55	9.07	18	16	6	12	44.97	48.55	2.9	3.18	227.3	150.9	4.8	4.59	0.79	0.91	103	105	1.19	1.05	1.1	1.23	4.31	4.09	2.34	2.37
12.9	9.5	4.42	6.9	1.69	2.64	118.2	116.3	34.59	36.09	183.22	260.86	40.82	40.96	9.55	8.3	14	13	12	10	44.8	46.25	3.2	2.58	191.8	203.2	4.66	5.07	0.92	0.8	102	102	1.12	1.25	1.14	1.1	4.35	4.19	2.08	2.18
11.2	11.7	4.42	6.9	2.57	2.64	121.9	119.6	34.15	33.65	235.74	198.64	40.81	42.36	7.65	12.51	15	10	20	8	42.05	41.75	3.6	3.08	168.9	194.3	3.16	4.92	0.87	0.78	103	102	1.13	2.06	1.23	1.08	4.16	4.12	2.21	2.34
9.5	6.5	8.12	6.44	2.56	2.84	124.6	132.6	35.2	33.92	253.36	166.22	42.21	42.77	9.55	9.41	22	15	14	4	43.73	42.38	3.4	2.58	221.2	164.1	5.2	4.86	0.85	0.93	103	102	1.34	1.10	1.1	1.14	3.91	3.91	2.18	2.36
9.8	10.4	4.42	6.2	1.97	2.84	132.6	125.4	34.42	34.7	227.53	198.75	39.38	40.97	11.85	10.2	17	15	15	12	13.3	45.25	2.5	2.38	182.1	209.3	5.21	4.59	0.92	0.78	102	102	2.15	1.11	1.16	1.23	4.24	4.16	2.28	2.22
8.2	8.3	4.42	4.97	2.43	2.44	121.6	118.2	33.7	36.72	215.75	126.75	42.58	42.73	12.64	8.3	23	12	12	12	40.3	45.18	3.3	3.28	222.0	204.0	4.91	3.09	0.81	0.86	107	104	1.19	1.03	1.23	1.1	3.91	4.12	2.22	2.18
8.3	7.7	5.09	8.3	1.89	2.54	118.2	116.3	34.75	34.09	277.86	260.86	39.6	42.36	7.65	12.5	18	21	20	8	46.38	14.75	2.7	3.08	168.9	214.0	5.14	4.82	0.79	0.84	103	101	2.14	2.07	1.08	1.29	4.12	4.24	2.46	2.23
11.2	10	4.42	9.5	2.12	2.54	116.3	137.8	34.15	33.65	220.19	198.64	41.54	39.75	8.76	10.37	14	20	17	3	44.55	46	3.2	3.48	182.1	194.3	5.2	4.74	0.79	0.78	105	102	1.12	2.05	1.1	1.16	3.91	4.23	2.36	2.22
12.9	7	4.42	8.6	1.42	2.34	125.4	125.6	37.22	34.7	284.53	218.74	42.62	40.96	9.72	13.9	17	17	20	4	45.9	74.5	2.7	2.78	212.3	164.1	3.16	4.73	0.83	0.8	102	104	2.15	1.25	1.23	1.1	4.09	3.91	2.33	2.08
8.2	7.7	4.42	7	1.75	2.54	125.6	119.6	33.7	33.92	215.64	193.5	42.21	41.69	7.65	12.41	18	16	8	11	42.05	47.83	2.5	3.08	224.1	194.3	4.66	4.59	0.96	0.91	103	101	1.19	2.06	1.14	1.23	3.91	4.17	2.37	2.28
9.5	8.6	4.42	8.3	1.69	2.84	127.6	132.6	37.21	33.2	253.36	264.23	41.58	42.53	11.86	10.2	18	20	17	1	40.91	42.36	3.4	3.48	168.9	173.8	4.93	4.84	0.83	0.78	105	102	1.34	1.11	1.1	1.14	4.16	4.12	2.18	2.46
7.1	9.4	4.42	6.4	1.76	2.94	121.9	124.8	34.75	34.25	143.75	203.19	42.58	39.53	8.42	8.3	12	16	20	12	43.73	41.75	2.8	3.28	168.9	150.9	4.66	3.09	0.94	0.82	103	105	1.19	1.03	1.23	1.1	4.24	4.35	2.22	2.37
7.7	11.7	7.45	5.09	2.35	2.34	132.6	116.3	34.15	33.65	277.86	260.86	40.81	42.36	9.72	13.2	22	12	15	10	43.8	43.62	2.9	2.38	191.8	194.3	5.21	5.07	0.81	0.78	103	102	2.15	1.10	1.29	1.36	4.31	4.23	2.36	2.18
10.6	11.7	4.42	4.42	2.43	2.54	118.2	118.2	34.42	36.72	281.23	166.22	42.38	42.73	7.65	8.3	19	16	20	9	46.38	45.18	3.6	3.18	182.1	194.3	5.2	4.59	0.87	0.91	103	102	1.12	1.05	1.19	1.23	4.24	3.91	2.34	2.22
11.2	7.7	5.09	6.56	2.12	2.44	124.6	132.6	35.2	33.2	183.22	193.5	39.52	40.97	11.85	12.51	17	15	17	8	73.05	48.55	3.2	2.58	212.3	164.1	4.8	4.73	0.79	0.8	102	102	1.19	1.11	1.23	1.08	4.12	4.16	2.23	2.21
8.2	8.3	4.42	4.42	1.89	2.24	125.6	125.4	35.56	36.71	227.53	210.53	42.21	40.96	9.72	10.37	14	11	17	12	40.3	43.5	3.3	3.08	222.0	209.3	4.91	4.86	0.92	0.88	105	104	1.13	1.25	1.1	1.1	3.91	4.35	2.46	2.34
9.5	11.7	4.42	6.46	2.47	2.24	116.3	116.3	36.39	34.25	253.36	198.75	42.62	39.67	11.98	9.41	18	20	17	12	42.05	46	2.5	2.58	232.0	194.3	4.99	5.14	0.85	0.84	103	102	2.15	2.06	1.14	1.23	4.09	4.24	2.23	2.36
10.7	8.6	4.42	6.32	2.57	2.34	125.4	113.6	34.15	33.65	220.19	198.64	39.6	41.73	11.76	12.5	13	15	20	14	44.55	46	2.9	2.38	168.9	164.1	4.93	5.13	0.83	0.82	103	102	2.15	1.03	1.23	1.16	4.23	3.91	2.18	2.28
9.8	11.3	4.42	6.46	1.75	2.94	137.8	125.6	35.2	33.92	277.86	260.86	39.05	42.36	7.65	13.9	17	10	6	4	44.8	43.5	2.9	3.28	212.3	204.0	4.66	3.09	0.79	0.91	105	101	1.34	1.08	1.16	1.1	4.31	4.12	2.22	2.46
8.2	8.3	4.42	6.32	1.76	2.04	119.6	127.6	37.25	34.7	215.75	193.5	42.58	42.77	9.55	9.07	18	16	20	15	43.73	45.18	3.2	2.68	182.1	150.9	4.59	4.92	0.81	0.78	106	102	1.12	1.11	1.1	1.14	4.24	4.31	2.21	2.33
9.5	7	6.32	7.09	2.43	2.44	122.3	132.6	34.59	35.06	230.15	213.15	39.38	42.73	8.42	8.3	15	16	17	10	42.17	41.75	3.4	2.78	222.0	204.0	3.16	4.52	0.92	0.78	103	105	1.19	2.07	1.23	1.29	4.35	3.91	2.28	2.22
8.9	10.9	7.09	5.09	1.97	3.14	121.9	118.2	35.2	35.89	215.64	267.53	42.21	40.96	11.85	13.31	18	16	15	14	46.38	42.36	2.7	3.48	168.9	150.9	4.91	4.73	0.79	0.8	102	101	1.17	1.10	1.08	1.36	3.91	4.09	2.18	2.18
12.5	8.6	5.09	11.3	1.42	2.34	127.6	121.9	33.7	33.65	253.36	203.19	40.82	40.97	7.65	13.29	12	12	8	17	47.1	41.75	3.2	3.08	182.1	194.3	5.2	4.84	0.89	0.91	103	102	1.19	1.25	1.39	1.23	4.12	3.91	2.36	2.08
12.9	10.9	4.42	11.7	1.89	2.2	125.6	125.4	37.21	34.7	235.74	218.74	42.62	41.73	8.76	10.37	22	13	20	7	13.3	45.18	2.9	3.18	182.5	164.5	5.21	4.59	0.83	0.95	103	105	1.13	1.03	1.23	1.1	3.91	3.96	2.37	2.28
8.3	7.1	4.42	7.1	2.39	2.1	118.2	124.6	35.56	36.75	277.86	193.5	41.54	42.53	9.72	12.5	17	20	17	14	42.05	74.5	2.8	2.38	212.3	164.1	4.91	3.09	0.87	0.91	103	102	1.19	1.25	1.14	1.29	4.24	3.91	2.22	2.37
8.9	8.6	8.12	7.7	2.56	1.9	116.3	125.6	35.2	34.09	249.27	260.86	41.58	42.36	8.42	13.2	23	15	15	3	43.8	43.5	3.6	2.78	182.1	150.9	4.66	5.13	0.92	0.78	105	104	1.12	1.11	1.1	1.23	4.17	4.23	2.46	2.36
9.5	11.7	8.12	8.3	2.21	1.8	125.4	131.2	33.7	34.7	230.15	198.75	40.81	41.73	7.65	12.51	22	21	20	15	44.55	39.5	3.2	2.78	191.8	194.3	4.93	4.59	0.81	0.78	103	102	2.15	2.06	1.23	1.14	3.91	3.96	2.34	2.22
11.2	10.4	4.42	10	2.47	2.4	119.6	137.8	34.15	33.2	220.19	210.53	42.21	39.2	11.86	8.3	14	16	20	17	43.73	45.18	2.8	3.08	168.9	194.3	4.8	5.14	0.96	0.82	103	101	1.34	1.10	1.29	1.1	4.16	3.96	2.36	2.46
9.5	7	4.42	8.3	2.43	2	124.6	118.2	34.75	36.71	253.36	198.64	42.58	42.73	11.76	9.41	18	12	20	4	40.3	47.83	2.5	3.28	212.3	194.3	3.16	4.82	0.79	0.8	102	102	2.14	1.11	1.1	1.16	4.24	4.24	2.33	2.18
12.9	8.3	4.42	5.7	1.76	2	124.8	125.6	37.22	35.06	227.53	193.5	39.6	40.96	11.85	13.9	13	15	20	13	41.8	39.5	2.9	2.58	182.1	194.3	4.66	3.09	0.92	0.91	105	104	1.19	1.03	1.23	1.23	4.31	3.96	2.22	2.21
7.7	9.5	5.09	6.9	1.78	2	121.9	113.6	37.24	34.7	277.86	166.22	40.82	42.36	7.65	8.3	18	16	17	8	40.91	45.18	2.7	3.08	222.0	164.1	4.81	5.13	0.89	0.84	103	102	1.19	1.05	1.16	1.08	3.91	4.12	2.18	2.34
11.6	7	4.42	6.2	1.75	2.2	118.2	125.4	36.59	33.2	183.22	260.86	39.05	41.73	8.42	13.2	17	16	14	17	44.8	45.25	3.3	2.78	227.3	214.0	4.99	5.13	0.92	0.88	103	104	1.12	1.25	1.14	1.14	4.12	3.91	2.21	2.37
12.9	6.5	4.42	6.9	1.89	2	121.6	119.6	34.15	33.65	215.75	213.15	42.21	42.53	12.66	10.37	22	10	17	10	42.05	46	2.5	2.68	212.3	194.3	3.16	4.59	0.79	0.91	102	105	1.17	1.03	1.23	1.23	4.35	4.16	2.08	2.22
9.8	10	4.42	4.97	1.69	1.7	119.6	121.6	34.42	34.25	215.64	264.23	39.6	42.77	9.72	10.2	12	20	20	4	73.05	14.75	3.2	3.48	182.1	164.1	5.21	4.73	0.81	0.82	103	102	1.19	1.11	1.1	1.1	4.31	3.91	2.46	2.28
8.2	5.9	4.42	6.9	2.21	2	125.4	113.6	35.2	36.72	253.36	193.5	39.52	40.96	7.53	12.63	17	17	15	7	46.38	43.5	3.4	3.08	236.7	150.9	4.66	4.84	0.83	0.8	105	101	1.12	1.25	1.08	1.39	4.23	3.96	2.37	2.33
12.9	11.3	4.42	6.9	2.35	2.1	116.3	136	34.59	36.74	281.23	203.19	41.54	42.53	9.78	12.5	13	15	20	4	43.73	74.5	3.2	2.68	212.3	204.0	4.93	4.59	0.92	0.78	107	102	2.15	1.03	1.23	1.23	4.24	3.91	2.22	2.34
8.3	8.6	4.42	6.9	2.43	2.1	127.6	142	37.49	36.09	277.86	198.75	42.58	40.97	7.65	8.3	18	12	17	15	44.55	41.75	2.9	3.48	168.9	173.8	4.91	5.13	0.79	0.91	103	102	1.34	2.07	1.16	1.14	4.31	4.12	2.28	2.18
12.1	7.7	5.09	6.34	1.42	2.4	125.6	140	34.15	34.7	230.15	260.86	42.21	42.36	8.42	13.9	18	16	20	15	47.1	42.36	3.2	3.18	222.0	150.9	3.16	4.73	0.94	0.86	102	102	1.12	1.03	1.1	1.1	4.12	3.91	2.18	2.46
10.7	11.7	6.56	4.3	2.57	2.5	118.2	136	35.56	34.25	220.19	198.64	40.81	42.53	8.76	9.41	12	11	8	7	40.3	45.18	2.7	2.68	182.1	150.9	4.8	4.86	0.87	0.78	106	104	1.13	1.25	1.23	1.23	3.91	4.31	2.36	2.22
12.9	10	4.42	6.2	1.97	2.1	121.9	155	33.7	34.75	227.53	193.5	40.82	41.69	11.86	12.51	19	15	17	15	43.8	41.75	2.5	2.78	191.8	204.0	5.14	4.73	0.89	0.84	103	105	1.19	1.05	1.14	1.16	4.16	3.91	2.34	2.23
7.7	8.3	6.46	4.3	1.76	1.9	124.6	136	34.42	33.92	143.75	218.74	42.62	42.73	7.65	8.3	22	16	15	7	42.05	48.55	2.7	3.08	168.9	150.9	4.66	4.59	0.81	0.8	103	102	2.14	2.07	1.36	1.1	4.24	3.96	2.21	2.22
11.6	8.6	6.32	4.3	1.75	2.2	132.6	175	35.2	36.99	253.36	232.27	39.6	39.75	8.42	10.37	18	13	20	8	46.38	46	3.2	3.28	224.1	194.3	5.21	4.89	0.92	0.82	103	101	1.13	1.11	1.23	1.23	4.12	4.24	2.22	2.46
9.5	7	6.46	4.3	1.69	2.1	125.4	142	33.7	33.2	277.86	166.22	42.21	39.67	9.72	13.9	18	16	20	13	41.8	43.5	3.4	2.38	182.1	150.9	3.16	3.09	0.83	0.91	103	101	1.19	1.03	1.18	1.14	4.19	3.91	2.37	2.22
8.9	7.1	8.12	8	2.47	2.4	122.3	169	37.49	34.7	235.74	260.86	41.54	42.77	13.25	12.5	17	10	20	15	43.73	45.25	3.4	2.68	182.5	150.9	4.93	4.59	0.85	0.78	105	102	1.12	1.25	1.08	1.36	4.24	4.16	2.46	2.18
11.2	10	8.12	4.3	2.43	1.8	121.9	132.6	37.24	33.92	215.75	193.5	42.38	42.36	7.65	13.29	17	20	17	15	13.3	42.36	3.6	3.08	212.3	194.3	4.66	5.13	0.79	0.93	102	102	2.15	1.03	1.23	1.1	3.91	4.12	2.18	2.36
8.2	9.5	8.12	4.3	1.78	2.2	116.3	125.4	33.7	33.2	183.22	210.53	42.62	42.53	9.55	13.2	14	15	14	13	44.55	46.25	3.2	2.58	222.0	173.8	4.8	5.07	0.92	0.86	103	102	1.34	1.08	1.16	1.23	4.24	3.91	2.33	2.28
8.9	7	6.46	4.97	2.39	3.05	125.6	118.2	36.39	34.25	215.64	126.75	42.58	41.73	8.42	9.07	12	21	16	8	47.1	45.18	2.9	2.38	232.0	164.1	4.89	4.73	0.92	0.78	105	102	2.14	1.11	1.1	1.08	4.12	3.96	2.23	2.22
9.8	8.3	6.46	4.3	2.12	1.9	137.8	125.4	34.15	34.75	277.86	203.19	41.58	40.96	11.85	13.9	23	20	20	13	46.38	41.75	2.8	3.08	168.9	204.0	4.8	4.59	0.79	0.82	102	104	2.14	1.25	1.23	1.14	4.26	3.91	2.22	2.23
10.6	8.6	6.46	4.3	1.42	1.9	127.6	116.3	33.7	33.2	253.36	198.64	42.21	40.97	7.65	10.37	22	12	17	10	42.05	74.5	3.3	3.18	212.3	206.1	3.16	4.86	0.89	0.8	103	104	1.19	2.07	1.29	1.23	4.12	3.91	2.36	2.46
12.9	8.3	6.46	4.3	1.76	2	118.2	119.6	34.42	33.92	220.19	193.5	40.82	42.73	9.55	10.2	18	16	14	13	40.3	43.5	2.5	2.78	182.1	194.3	4.91	4.86	0.87	0.91	106	102	1.12	1.03	1.08	1.1	3.91	4.24	2.23	2.18
12.9	7.7	6.46	4.3	2.54	2.1	125.4	114.9	35.2	34.25	227.53	260.86	39.05	42.36	12.55	8.3	18	11	17	8	40.91	48.55	3.4	3.08	191.8	150.9	3.16	4.59	0.81	0.91	103	102	1.19	1.03	1.23	1.29	4.09	4.24	2.28	2.22
8.9	10.9	6.46	4.3	1.69	2	124.6	116.3	33.7	33.2	284.53	198.75	40.81	42.36	7.65	9.41	15	16	17	13	43.73	46	2.7	2.58	212.3	214.0	5.26	3.09	0.79	0.78	103	104	1.17	1.03	1.14	1.1	4.24	4.26	2.46	2.46
9.5	10.9	6.46	7.33	2.43	2.3	124.8	125.4	34.15	36.99	281.23	213.15	39.38	42.53	9.72	12.51	13	15	20	15	46.38	45.18	3.2	3.48	168.9	164.1	4.66	5.14	0.79	0.88	103	102	1.13	1.25	1.39	1.14	4.31	4.12	2.18	2.22
8.2	8.3	7.09	4.3	2.56	2.3	121.9	125.6	34.59	33.92	277.86	218.74	42.21	39.75	11.85	10.43	18	20	20	13	40.93	41.75	3.6	2.58	227.3	204.0	4.93	4.73	0.92	0.82	105	101	1.34	1.10	1.23	1.16	4.12	3.91	2.22	2.36
9.8	8.6	6.46	4.97	1.65	1.7	125.6	127.6	33.7	33.2	249.27	193.5	42.62	39.2	11.86	13.9	17	10	15	13	44.55	42.36	2.7	3.08	212.3	164.1	4.91	4.84	0.83	0.8	103	102	2.15	1.11	1.36	1.23	3.91	4.12	2.34	2.22
9.5	8.3	5.09	4.3	1.89	2.1	122.3	125.4	36.39	34.25	215.75	264.23	39.6	42.36	13.25	12.5	18	15	8	8	43.8	74.5	2.9	2.68	182.1	203.2	4.66	4.59	0.92	0.78	103	102	1.12	1.03	1.1	1.1	4.19	3.91	2.33	2.37
11.6	11.7	6.32	4.3	2.21	2	118.2	116.3	35.56	34.09	183.22	260.86	39.52	42.73	8.76	10.37	14	11	17	13	42.05	46	3.2	2.58	222.0	194.3	4.8	4.86	0.81	0.86	102	105	1.19	2.05	1.23	1.23	4.24	4.24	2.22	2.33
8.3	8.6	6.46	4.3	2.47	2.1	127.6	119.6	33.7	33.2	220.19	210.53	40.82	40.97	11.98	10.2	22	16	17	9	46.38	43.5	3.4	2.38	224.1	150.9	4.81	5.13	0.92	0.78	103	102	1.19	1.11	1.08	1.39	4.16	3.96	2.28	2.22
7.7	8.3	7.45	4.3	2.57	1.9	125.4	132.6	34.42	33.92	277.86	203.19	42.21	42.36	7.65	13.9	17	16	15	13	45.9	45.25	2.9	3.08	212.3	164.1	3.16	4.59	0.79	0.84	106	102	2.14	1.25	1.16	1.14	4.35	4.16	2.08	2.18
9.5	10	8.12	4.3	2.35	2.5	119.6	125.4	35.2	34.25	227.53	193.5	41.54	39.75	11.76	8.3	18	10	20	8	43.73	41.75	3.2	3.28	168.9	164.1	4.91	4.82	0.89	0.82	102	101	1.17	2.07	1.23	1.1	3.91	3.91	2.36	2.34
8.2	7.7	6.46	6.2	2.43	2	132.6	118.2	33.7	33.2	235.74	198.64	39.05	39.2	9.55	13.31	12	17	17	15	47.1	42.36	2.5	2.78	222.0	150.9	5.14	3.09	0.96	0.91	103	104	1.19	1.11	1.1	1.23	4.31	4.12	2.18	2.22
9.5	10	6.46	6.97	1.42	2.1	124.6	116.3	37.24	35.06	215.64	166.22	40.81	39.67	12.26	9.41	12	20	20	13	40.3	45.18	3.3	2.68	182.1	204.0	4.93	5.07	0.83	0.8	106	102	1.12	1.03	1.16	1.08	4.12	4.24	2.46	2.23
12.9	8.3	6.46	4.97	1.75	2.3	131.2	137.8	34.15	33.92	230.15	260.86	41.58	42.53	11.85	12.51	14	16	20	13	73.05	46	3.2	3.08	212.3	194.3	3.16	4.59	0.79	0.95	103	104	1.34	2.06	1.23	1.16	4.23	3.91	2.22	2.46
9.8	8.6	6.32	4.3	2.12	1.88	125.6	125.6	33.7	33.2	253.36	198.75	42.21	41.73	11.86	13.29	17	16	6	13	44.55	43.25	2.8	3.18	236.7	206.1	4.66	4.73	0.81	0.78	105	103	1.34	1.11	1.14	1.1	4.31	4.31	2.37	2.22
8.9	7	6.56	4.3	1.78	2	137.8	119.6	37.21	34.25	277.86	267.53	42.58	42.53	7.65	10.37	22	15	17	1	42.05	45.18	2.9	2.78	222.0	164.1	4.8	4.86	0.87	0.86	103	106	2.15	1.10	1.1	1.14	3.91	4.16	2.33	2.21
10.7	9.5	6.46	8	2.39	1.8	119.6	132.6	35.56	34.7	284.53	193.5	39.38	42.73	8.76	13.2	18	17	20	13	42.17	46	3.2	3.08	182.1	214.0	4.99	3.09	0.79	0.86	102	101	1.19	1.03	1.23	1.23	4.24	4.12	2.34	2.22
7.1	10	6.46	8	2.73	1.8	125.4	124.8	33.7	33.65	281.23	213.15	40.82	42.36	13.25	13.9	18	20	8	13	47.1	43.5	3.4	3.28	168.9	150.9	4.66	4.84	0.92	0.8	103	102	1.13	1.25	1.29	1.23	4.17	3.91	2.28	2.46
12.5	10.9	6.46	4.3	2.56	2	122.3	116.3	34.15	33.2	220.19	198.64	42.62	40.96	11.98	10.2	23	10	15	15	43.73	43.62	2.7	2.58	191.8	194.3	4.96	4.73	0.85	0.91	105	102	1.12	1.08	1.08	1.23	4.26	3.91	2.18	2.18
9.5	8.6	6.46	4.3	1.4	2.2	127.6	118.2	34.42	34.25	143.75	260.86	42.21	40.97	12.55	9.07	18	10	17	15	43.8	45.18	2.7	2.58	212.3	164.1	3.16	4.59	0.92	0.82	103	102	1.19	1.03	1.23	1.1	4.12	3.96	2.22	2.36
11.2	10	6.46	4.3	1.75	2.1	118.2	132.6	33.7	33.65	277.86	203.19	39.6	39.75	9.72	8.3	17	10	20	10	40.91	14.75	2.5	3.08	224.1	204.0	5.21	5.13	0.79	0.86	105	104	1.19	1.03	1.19	1.29	3.91	4.35	2.21	2.28
8.2	7	6.46	4.97	2.47	1.9	131.2	125.4	34.75	36.09	249.27	210.53	42.21	42.77	12.66	13.9	15	12	14	3	46.38	41.75	3.2	2.78	232.0	150.9	4.8	4.86	0.81	0.78	106	102	1.34	1.11	1.1	1.18	4.24	3.96	2.46	2.22
9.5	8.3	6.46	3.9	1.55	1.8	124.6	116.3	35.2	33.2	183.22	218.74	39.52	41.69	7.65	8.3	18	16	20	15	13.3	42.36	2.9	2.38	182.1	173.8	4.93	5.14	0.89	0.91	103	104	1.13	1.25	1.23	1.1	4.12	3.91	2.36	2.33
9.8	8.6	6.56	7.1	1.97	1.196	121.6	113.6	33.7	33.92	215.64	193.5	42.38	42.36	11.98	8.3	22	15	17	13	42.05	41.75	3.4	3.28	212.3	164.1	4.8	3.09	0.79	0.8	102	102	2.14	1.11	1.14	1.23	4.16	4.24	2.23	2.34
11.2	7	6.46	8.6	1.42	1.223	125.6	125.6	34.59	33.65	253.36	267.53	42.58	42.73	12.64	10.2	22	12	17	13	44.55	74.5	2.8	3.08	222.0	150.9	4.66	4.59	0.85	0.88	103	102	1.12	1.03	1.39	1.14	4.31	3.91	2.37	2.22
12.9	10.4	5.09	7.7	2.12	1.219	125.4	127.6	36.59	34.25	215.75	260.86	40.81	42.36	11.86	10.37	17	16	20	9	40.3	43.5	3.2	3.48	213.9	194.3	4.99	4.92	0.79	0.78	103	102	1.19	1.08	1.23	1.16	3.91	4.16	2.22	2.46
9.5	11.3	6.46	8.6	1.55	2.3	118.2	132.6	34.42	33.2	277.86	166.22	40.82	42.53	7.65	12.51	13	20	20	13	43.73	46	2.6	3.18	168.9	173.8	4.91	4.84	0.92	0.91	103	101	1.17	1.03	1.36	1.23	4.24	3.91	2.18	2.46
9.8	8.3	6.46	7.7	1.75	2.2	132.6	118.2	35.56	34.7	235.74	198.75	42.38	39.75	8.76	9.41	12	15	17	15	44.97	45.18	3.4	2.58	191.8	164.1	3.16	4.59	0.83	0.86	105	102	2.15	1.25	1.14	1.23	4.09	3.96	2.08	2.18
7.1	8.6	6.46	7	2.57	2.1	137.8	121.9	34.15	33.65	220.19	198.64	41.54	40.97	8.42	9.07	18	16	15	15	46.38	45.25	3.3	2.68	168.9	204.0	5.14	4.86	0.81	0.82	106	102	1.19	1.03	1.23	1.1	4.12	4.12	2.34	2.22
9.5	10	4.42	8.3	1.69	1.8	127.6	125.4	37.25	34.25	249.27	203.19	39.38	40.96	9.72	12.5	18	16	15	13	47.1	47.83	3.2	3.08	212.3	194.3	4.99	4.73	0.87	0.8	103	104	1.34	1.11	1.16	1.08	4.35	3.96	2.46	2.08
10.7	8.6	4.42	4.1	1.78	1.8	131.2	124.6	37.21	33.2	230.15	193.5	41.58	42.36	9.55	13.9	19	16	17	13	44.8	46.25	2.5	2.78	182.1	209.3	3.16	5.13	0.79	0.78	102	104	1.12	2.07	1.1	1.14	3.91	3.91	2.21	2.37
8.2	8.3	4.42	5.3	1.89	1.7	124.6	125.6	36.39	34.09	253.36	260.86	42.38	39.53	11.85	12.63	14	20	17	18	42.05	41.75	2.7	3.28	212.3	195.9	4.66	4.59	0.94	0.82	102	102	1.19	1.11	1.23	1.1	4.23	4.23	2.22	2.36
9.8	7.7	5.09	5.09	1.55	2.4	116.3	131.2	34.42	33.65	277.86	264.23	39.6	39.75	11.85	10.37	18	16	20	13	43.8	47.83	2.8	3.08	222.0	164.1	4.93	5.14	0.85	0.93	103	105	1.13	1.03	1.08	1.19	4.31	3.96	2.36	2.33
7.7	8.3	4.42	5.12	1.75	2.1	121.6	137.8	34.15	33.92	230.15	210.53	42.58	40.97	7.65	8.3	22	17	8	15	44.55	43.5	3.4	3.48	212.3	150.9	4.91	3.09	0.79	0.86	103	102	1.17	1.10	1.14	1.23	4.12	4.12	2.18	2.22
12.9	6.5	4.42	3.94	1.58	1.9	125.6	118.2	34.59	33.2	227.53	126.75	42.62	42.73	8.42	13.9	17	16	20	13	43.73	41.75	3.2	2.78	182.5	194.3	4.89	4.86	0.92	0.82	103	106	2.14	2.07	1.23	1.1	4.19	3.96	2.28	2.21
9.8	11.3	4.42	4.97	1.75	2.1	122.3	119.6	37.21	34.25	143.75	213.15	42.38	39.2	7.65	9.07	17	16	20	15	44.97	45.18	2.5	3.08	224.1	204.0	4.8	4.92	0.81	0.91	103	102	1.13	1.03	1.29	1.16	3.91	3.91	2.33	2.22
9.5	8.6	8.09	6.34	2.54	2	119.6	121.9	37.22	34.7	215.64	260.86	40.82	39.2	13.25	12.5	13	16	17	10	73.05	46	3.6	2.68	212.3	164.1	4.66	4.73	0.89	0.8	105	104	1.12	1.25	1.1	1.14	4.09	4.16	2.28	2.28
11.2	8.3	8.09	6.34	2.12	2	121.9	125.4	37.21	34.25	277.86	193.5	39.38	40.96	11.76	8.3	23	10	23	13	40.3	74.5	2.7	2.38	227.3	206.1	3.16	4.84	0.92	0.78	105	102	1.34	1.03	1.23	1.23	4.12	4.35	2.22	2.18
12.5	7.7	5.09	6.34	1.42	2.2	127.6	123.788	33.7	33.2	253.36	218.74	40.81	42.36	9.72	12.51	13	15	8	3	40.91	48.55	3.2	2.58	168.9	150.9	4.66	4.86	0.79	0.84	103	102	1.19	1.11	1.16	1.1	4.24	3.91	2.46	2.46
7.1	8.3	8.09	4.3	1.89	2.3	119.6	123.788	36.59	35.06	220.19	198.64	42.38	39.75	12.55	10.2	18	20	17	15	13.3	47.83	3.4	3.08	182.1	194.3	5.14	4.59	0.79	0.95	103	102	2.15	1.05	1.1	1.18	4.31	4.24	2.37	2.34
8.9	10	8.09	4.3	2.39	1.7	131.2	141.472	35.56	33.92	183.22	203.19	39.05	41.73	8.42	9.41	23	12	17	13	42.05	43.5	2.5	2.78	191.8	164.5	5.26	5.13	0.87	0.86	105	101	1.17	1.11	1.23	1.23	3.91	3.91	2.18	2.37
10.6	8.6	6.32	4.3	1.55	1.8	118.2	141.472	34.59	34.25	215.75	166.22	42.62	42.73	7.65	10.37	22	20	20	15	47.1	41.75	2.9	3.28	212.3	164.1	3.16	3.09	0.83	0.78	103	102	1.19	2.06	1.29	1.08	4.23	4.31	2.34	2.36
11.2	8.3	8.09	4.97	2.47	2	124.6	106.104	37.21	33.2	281.23	198.75	42.58	39.67	11.86	9.07	12	16	15	13	43.73	43.62	3.3	3.08	212.3	204.0	4.93	4.59	0.81	0.88	102	104	1.12	1.10	1.14	1.23	4.12	3.91	2.36	2.22
9.5	7	8.09	4.3	2.56	2.2	125.6	114.946	34.15	36.74	277.86	210.53	42.38	42.77	8.76	8.3	18	15	20	13	44.55	45.18	2.7	3.18	222.0	194.3	4.8	4.74	0.85	0.8	103	102	1.19	1.25	1.23	1.1	4.16	3.96	2.22	2.33
9.8	5.9	8.09	4.3	2.35	1.8	132.6	114.946	37.49	33.65	227.53	193.5	39.52	42.36	9.55	13.2	17	10	9	15	43.8	45.25	3.2	2.78	212.3	150.9	4.91	4.86	0.89	0.91	103	105	1.34	1.03	1.1	1.14	4.12	4.24	2.18	2.18
12.5	7.7	8.09	4.3	1.75	1.9	121.9	97.262	34.75	34.7	253.36	232.27	40.82	40.96	7.65	12.41	15	16	14	15	41.8	46	2.7	3.08	168.9	164.1	3.16	4.73	0.85	0.78	106	102	1.13	1.11	1.16	1.23	3.91	4.12	2.23	2.21
9.5	10	8.12	7.97	2.57	2	118.2	114.946	35.2	35.89	235.74	264.23	42.21	40.97	11.85	8.3	17	12	17	15	42.17	41.75	2.9	2.58	212.3	173.8	4.66	3.09	0.79	0.84	103	104	2.14	2.07	1.23	1.1	4.31	4.09	2.22	2.23
10.7	8.3	8.09	7.97	1.69	2.2	137.8	141.472	36.39	33.92	143.75	198.64	42.38	39.53	9.72	13.9	14	20	20	15	45.9	43.5	3.4	3.28	182.1	209.3	5.21	4.59	0.92	0.82	103	102	1.34	1.08	1.18	1.16	4.35	3.96	2.08	2.22
7.7	8.6	8.09	4.97	1.89	2.3	137.8	150.314	34.15	33.65	220.19	213.15	41.58	39.75	13.25	13.2	18	10	17	13	40.3	43.5	3.2	2.58	212.3	164.1	4.81	4.73	0.85	0.91	102	102	1.12	2.07	1.29	1.23	3.91	3.91	2.28	2.37
11.2	8.3	8.09	7.97	2.12	2.3	125.6	97.262	37.21	34.09	277.86	166.22	39.6	42.73	11.76	9.07	22	15	17	9	42.05	45.18	3.6	3.08	212.3	194.3	5.2	5.07	0.81	0.93	105	102	2.15	2.06	1.23	1.1	4.31	4.16	2.46	2.28
9.5	11.7	8.09	6.5	1.78	2.9	124.6	114.946	33.7	34.7	215.75	218.74	41.54	42.36	9.55	8.3	18	16	15	13	43.73	46.25	3.3	2.38	168.9	173.8	4.66	4.59	0.83	0.8	103	102	1.17	1.03	1.1	1.29	4.12	3.96	2.37	2.18
9.5	8.6	8.09	8.3	1.55	1.378	127.6	106.104	34.42	36.71	215.64	236.36	42.38	42.53	7.65	10.37	22	16	20	13	47.1	45.18	3.4	3.28	221.2	204.0	5.14	4.86	0.87	0.88	105	101	1.19	1.11	1.08	1.23	4.24	4.17	2.18	2.21
8.2	7.1	8.09	8.3	1.42	1.276	121.9	88.42	37.49	33.65	253.36	210.53	40.81	42.36	12.55	12.51	12	13	9	10	44.55	41.75	2.7	2.38	212.3	164.1	4.99	4.84	0.96	0.78	102	102	1.34	1.25	1.23	1.1	4.17	3.96	2.22	2.36
11.2	6.5	6.32	10.9	2.63	1.182	122.3	88.42	34.59	35.06	281.23	198.75	42.38	41.73	7.65	8.3	17	15	17	15	40.91	39.5	2.7	3.08	182.1	150.9	4.93	4.59	0.89	0.91	102	104	2.14	1.10	1.39	1.19	3.91	4.24	2.21	2.22
7.1	8.3	8.09	8.6	2.21	1.254	124.6	97.262	37.24	33.92	227.53	260.86	42.58	42.53	11.86	13.9	18	16	15	15	46.38	45.18	2.8	2.38	227.3	206.1	4.66	4.86	0.79	0.86	103	101	1.12	1.11	1.1	1.23	4.12	3.91	2.34	2.23
9.8	8.3	7.09	7.7	1.89	1.246	116.3	150.314	37.25	34.7	277.86	203.19	42.21	42.77	9.72	12.5	12	20	13	15	43.8	43.5	3.2	2.38	222.0	194.3	3.16	4.73	0.79	0.84	106	101	1.19	1.05	1.23	1.08	4.24	4.31	2.36	2.46
9.5	10.9	8.09	5.9	1.97	1.248	121.6	70.736	34.42	33.65	183.22	267.53	39.38	40.96	8.76	8.3	18	16	9	13	42.17	48.55	3.4	2.38	232.0	150.9	4.89	3.09	0.81	0.91	103	102	2.14	1.11	1.16	1.1	4.16	3.96	2.28	2.22
12.1	8.6	5.09	8.3	2.47	1.219	118.2	106.104	36.59	36.09	230.15	198.64	40.82	41.69	11.85	8.3	12	17	17	9	46.38	45.18	2.5	3.08	212.3	214.0	4.81	5.13	0.92	0.8	103	105	1.34	1.03	1.14	1.23	4.31	4.16	2.46	2.18
8.3	7.7	8.09	8.3	2.54	1.256	119.6	167.998	35.56	33.2	220.19	236.36	42.38	42.36	13.25	9.41	22	16	17	13	43.73	43.5	3.6	2.38	182.1	164.1	4.8	5.19	0.83	0.78	105	102	2.15	2.05	1.23	1.14	3.91	4.24	2.33	2.22
9.5	5.9	8.09	10	1.55	2.47	125.6	97.262	37.24	34.09	215.75	126.75	42.62	42.73	8.42	12.5	17	16	13	15	42.05	39.5	2.9	2.78	212.3	194.3	4.66	4.59	0.92	0.78	102	102	1.19	1.25	1.1	1.29	4.12	4.12	2.22	2.21
8.3	8.3	8.09	8.6	2.12	2.56	124.6	123.788	34.59	33.65	277.86	260.86	39.52	42.36	7.65	10.37	18	15	8	15	44.8	45.18	3.3	2.68	191.8	204.0	4.91	4.92	0.85	0.82	102	104	1.12	1.08	1.18	1.23	4.09	3.91	2.18	2.18
12.5	8.3	8.09	9.5	2.56	2.35	118.2	106.104	34.42	34.7	281.23	264.23	39.38	42.53	12.66	8.3	18	12	9	7	44.55	45.25	3.4	3.08	168.9	218.7	3.16	4.59	0.87	0.95	103	101	1.34	2.06	1.29	1.1	4.19	3.96	2.08	2.36
12.9	10	8.09	6.5	1.42	1.254	132.6	123.788	34.15	33.2	235.74	166.22	42.21	39.75	12.64	12.5	19	10	16	15	40.3	47.83	2.9	3.48	212.3	164.1	5.14	3.09	0.79	0.82	107	101	1.17	1.11	1.1	1.16	4.12	4.23	2.28	2.46
11.2	8.6	8.09	7.33	2.35	2.57	121.9	106.104	36.39	33.92	227.53	210.53	42.62	40.97	9.72	10.2	17	16	6	18	73.05	39.5	3.2	2.78	212.3	150.9	4.66	5.14	0.81	0.93	102	102	2.14	1.03	1.23	1.23	3.91	4.09	2.37	2.28
8.2	9.5	8.09	6.2	2.57	2.56	127.6	88.42	36.59	36.74	215.64	236.36	42.58	40.96	11.85	12.51	13	20	17	3	46.38	45.18	3.6	3.18	182.1	194.3	4.8	4.59	0.83	0.8	103	106	1.19	1.11	1.23	1.08	4.16	4.24	2.36	2.22
9.8	6.5	5.09	4.3	2.21	1.97	122.3	132.63	33.7	33.65	277.86	203.19	39.6	42.36	12.55	8.3	14	15	15	13	47.1	39.5	2.9	3.08	227.3	164.5	4.93	4.73	0.89	0.86	103	101	1.19	2.07	1.14	1.1	4.12	3.96	2.22	2.18
8.9	8.6	8.09	4.3	1.4	2.43	125.6	97.262	37.24	33.2	253.36	260.86	40.82	39.53	11.86	9.07	22	20	14	10	41.8	45.18	3.4	3.28	212.3	150.9	5.21	5.13	0.92	0.78	105	102	1.12	1.03	1.16	1.23	4.24	3.91	2.46	2.36
8.3	8.3	8.09	4.97	1.55	1.89	127.6	70.736	35.56	35.89	143.75	198.75	42.38	42.73	7.65	12.63	18	16	13	15	13.3	74.5	2.7	2.78	182.1	164.1	5.2	4.59	0.83	0.91	106	102	1.17	1.25	1.1	1.14	4.23	4.16	2.18	2.46
10.7	7.7	8.09	4.3	1.78	2.12	121.9	97.262	35.2	34.7	220.19	213.15	42.21	39.75	8.76	10.37	15	16	17	7	42.05	42.36	3.2	3.08	222.0	204.0	3.16	4.86	0.79	0.84	103	104	1.34	1.05	1.23	1.16	3.91	4.12	2.21	2.33
8.2	8.3	8.09	8.3	2.39	1.42	122.3	123.788	34.75	35.06	183.22	198.64	42.38	41.69	13.25	8.3	19	12	17	7	44.55	45.18	2.8	2.58	168.9	194.3	4.99	4.84	0.85	0.82	102	105	1.13	1.03	1.24	1.23	4.17	4.24	2.34	2.18
7.7	10.4	8.12	8.6	2.47	1.75	132.6	114.946	34.42	33.2	215.75	236.36	41.58	42.77	7.65	13.2	17	15	19	14	40.93	47.83	3.3	2.38	222.0	203.2	4.59	4.86	0.79	0.78	103	102	1.19	1.10	1.23	1.1	4.12	3.96	2.37	2.34
12.1	7	8.09	10	2.73	1.69	125.6	79.578	37.49	33.92	277.86	218.74	40.81	42.36	12.55	13.9	18	11	9	9	43.8	46.25	2.9	2.38	168.9	206.1	4.8	3.09	0.81	0.8	106	101	2.15	1.11	1.39	1.39	4.35	3.96	2.22	2.46
9.8	7.7	5.09	8.6	1.69	1.76	121.9	150.314	37.24	34.09	253.36	260.86	42.38	41.73	9.72	9.41	14	20	23	15	46.38	41.75	2.5	3.08	212.3	204.0	4.91	4.59	0.92	0.91	105	102	1.12	1.25	1.1	1.23	4.23	3.91	2.28	2.37
9.5	11.7	8.09	8.3	1.75	2.35	119.6	132.63	35.56	34.25	253.36	232.27	40.82	42.73	9.55	13.29	15	20	8	15	40.91	46	3.4	2.58	182.5	164.1	4.66	5.13	0.85	0.78	105	105	1.19	1.03	1.23	1.36	3.91	4.19	2.33	2.22
8.9	9.4	4.42	7.7	2.54	2.43	118.2	88.42	35.2	33.2	183.22	213.15	42.58	40.96	11.98	8.3	12	16	18	7	44.8	43.5	3.2	2.78	182.1	194.3	3.16	4.82	0.79	0.88	103	104	1.34	1.08	1.16	1.1	4.16	4.31	2.34	2.18
11.2	8.6	7.45	8.3	2.56	2.12	125.6	97.262	33.7	33.65	235.74	203.19	39.6	42.53	11.85	13.9	22	12	15	7	47.1	74.5	2.7	2.58	168.9	173.8	5.2	3.09	0.92	0.82	103	104	1.19	1.11	1.19	1.23	4.35	3.91	2.18	2.18
12.9	8.3	6.32	6.5	2.21	1.216	124.6	114.946	34.75	34.7	215.64	210.53	42.38	39.67	7.65	13.9	18	15	20	7	43.73	47.83	3.4	3.08	212.3	214.0	4.66	4.59	0.87	0.86	102	102	1.17	1.03	1.23	1.14	4.24	4.24	2.46	2.22
7.1	10.4	4.42	5.3	2.35	1.256	116.3	110.525	34.59	36.99	220.19	264.23	40.82	42.36	13.25	12.51	17	16	12	10	44.55	43.25	3.2	2.68	212.3	150.9	5.21	4.73	0.92	0.91	105	102	2.15	1.25	1.1	1.08	3.91	3.96	2.22	2.36
8.3	8.3	4.42	8.6	2.39	2.12	119.6	119.367	33.7	33.2	227.53	193.5	42.58	42.77	8.76	8.3	18	16	12	15	44.55	45.25	2.9	3.28	212.3	150.9	4.89	5.07	0.79	0.8	103	101	1.12	2.06	1.23	1.23	4.12	4.12	2.23	2.46
9.8	7.7	5.09	8.3	1.58	2.56	127.6	111.4092	34.42	36.09	277.86	210.53	42.21	39.75	11.86	10.37	17	16	19	15	47.1	47.83	3.4	2.78	212.3	164.1	3.16	4.86	0.83	0.95	103	104	1.19	1.10	1.34	1.42	4.31	3.96	2.22	2.33
8.9	10	4.42	7.5	1.97	2.57	125.6	106.104	34.15	33.65	253.36	260.86	42.38	39.2	7.65	8.3	18	15	14	13	43.73	43.5	2.9	3.28	182.1	194.3	5.2	5.13	0.81	0.78	103	102	1.34	1.03	1.23	1.17	3.91	4.34	2.18	2.22

**Table 11:** Data file. Serum electrolytes. PLT: platelet count; WBC: white blood cell count; AST: aspartate aminotransferase; ALT: alanine aminotransferase; Cr: creatinine; UA: uric acid; FBG: fasting blood glucose; Mg: magnesium; Cl: chloride; TSH: thyroid-stimulating hormone

WBC (×10^9^/L) PG	WBC (×10^9^/L) CG	Neutrophil count (×10^9^/L) PG	Neutrophil count (×10^9^/L) CG		Lymphocyte count (×10^9^/L) PG	Lymphocyte count (×10^9^/L) CG		Hemoglobin (g/L) PG	Hemoglobin (g/L) CG	Hematocrit (%) PG	Hematocrit (%) CG	PLT (×10^9^/L) PG	PLT (×10^9^/L) CG		Albumin (g/L) PG		Albumin (g/L) CG	Total bilirubin (μmol/L) PG		Total bilirubin (μmol/L) CG	AST (U/L) PG	AST (U/L) CG	ALT (U/L) PG	ALT (U/L) CG	Cr (μmol/L) PG	Cr (μmol/L) CG	Urea (mmol/L) PG	Urea (mmol/L) CG	UA (μmol/L) PG	UA (μmol/L) CG	FBG (mmol/L) PG	FBG (mmol/L) CG	Mg (mmol/L) PG	Mg (mmol/L) CG	Cl (mmol/L) PG	Cl (mmol/L) CG	TSH (mIU/L) PG	TSH (mIU/L) CG	Free thyroxine (ng/dL) PG	Free thyroxine (ng/dL) CG	Serum potassium (mmol/L) PG	Serum potassium (mmol/L) CG	Blood calcium (mmol/L) PG	Blood calcium (mmol/L) CG
12.1	10.9	4.42	4.07	4.3	2.63		2.47	125.6		35.2	34.7	227.53	253.36	236.36	40.97	40.82	39.53	9.25	10.2	9.55	11.6		21		43.5		3.3		235.5		4.92		0.96		107		1.34		1.36		4.31		2.21	2.37
9.5	8.3	4.42	4.07	4.3	1.36		2.69	113.6		34.75	34.25	281.23	227.53	210.53	39.67	39.52	40.97	11.96	12.91	12.26	12		16		45.18		2.8		215.75		4.38		0.79		102		1.116		1.18		4.09		2.36	2.28
9.8	8.6	6.32	5.97	6.2	1.40		2.94	125.4		35.25	34.75	210.5	277.86	260.86	42.53	42.38	42.53	11.55	12.5	11.85	13.95		20		46.25		2.9		185.55		4.52		0.81		103		1.194		1.23		3.91		2.22	2.22
9.5	8.3	8.12	7.77	8	2.73		2.94	119.6		34.42	33.92	227.53	183.22	166.22	42.36	42.21	42.77	7.35	8.3	7.65	18		23		45.18		3.2		172.35		4.63		0.96		103		1.34		1.08		3.96		2.23	2.46
12.9	11.7	6.32	5.97	6.2	1.69		2.84	121.6		37.49	36.99	277.86	215.75	198.75	42.73	42.58	39.67	8.12	9.07	8.42	17		13		41.75		3.4		225.45		4.38		0.79		103		1.116		1.23		3.91		2.46	2.18
9.8	8.6	4.42	4.07	4.3	1.75		2.54	113.6		33.7	33.2	220.19	215.64	198.64	42.53	42.38	39.53	9.42	10.37	9.72	13.7		20		39.5		2.5		195.25		4.92		0.81		105		2.152		1.1		4.23		2.22	2.22
9.5	8.3	5.09	4.74	4.97	1.40		2.44	125.4		35.2	34.7	210.5	253.36	236.36	40.96	40.81	42.36	7.35	8.3	7.65	18		9		45.18		2.8		172.35		4.52		0.92		103		1.116		1.23		3.96		2.18	2.36
11.2	10	4.42	4.07	4.3	1.69		2.14	116.3		34.42	33.92	215.64	281.23	264.23	42.36	42.21	42.77	6.75	7.7	7.05	22		21		43.5		3.2		172.35		4.65		0.87		105		1.34		1.14		3.96		2.22	2.34
8.9	7.7	5.09	4.74	4.97	1.36		2.44	118.2		33.7	33.2	227.53	277.86	260.86	42.53	42.38	42.73	11.56	12.51	11.86	16.5		23		48.55		2.7		225.45		4.52		0.83		103		1.132		1.16		4.24		2.21	2.22
11.2	10	4.42	4.07	4.3	1.40		2.34	125.4		34.75	34.25	277.86	230.15	213.15	40.97	40.82	39.75	9.42	10.37	9.72	18		10		45.18		2.5		172.35		4.38		0.79		103		2.152		1.1		3.96		2.18	2.21
9.5	8.3	6.56	6.21	6.44	1.40		2.54	129.4		35.25	34.75	210.5	220.19	203.19	39.75	39.6	40.97	8.12	9.07	8.42	17.75		19		43.5		3.2		215.75		4.68		0.92		103		1.194		1.23		4.12		2.36	2.36
9.8	8.6	4.42	4.07	4.3	1.42		2.34	118.2		33.7	33.2	235.74	227.53	210.53	42.73	42.58	42.53	6.75	7.7	7.05	18		14		39.5		3.3		172.35		2.88		0.89		102		1.116		1.1		3.91		2.46	2.46
8.2	7	6.46	6.11	6.34	2.12		2.44	119.6		34.42	33.92	220.19	143.75	126.75	39.53	39.38	42.36	8.46	9.41	8.76	22		23		45.18		2.9		172.35		4.38		0.81		103		1.34		1.23		4.16		2.28	2.18
10.7	9.5	6.32	5.97	6.2	1.75		3.04	114.9		34.75	34.25	227.53	253.36	236.36	42.36	42.21	42.53	9.75	10.7	10.05	17.75		16		45.25		3.2		215.75		4.92		0.79		103		1.116		1.14		3.91		2.22	2.22
11.2	10	6.46	6.11	6.34	1.40		2.74	127.6		33.7	33.2	210.5	277.86	260.86	40.97	40.82	41.73	6.75	7.7	7.05	19.4		10		47.83		2.7		195.25		4.86		0.79		105		1.168		1.29		3.96		2.18	2.21
12.1	10.9	6.32	5.97	6.2	1.36		1.74	132.6		37.49	36.99	277.86	235.74	218.74	42.77	42.62	40.96	11.55	12.5	11.85	17.5		13		39.5		3.6		185.55		4.52		0.81		105		1.194		1.1		3.91		2.36	2.33
9.8	8.6	7.09	6.74	6.97	1.55		2.74	125.4		34.42	33.92	215.64	215.75	198.75	40.96	40.81	42.53	9.25	10.2	9.55	17.75		10		45.18		2.7		225.45		4.38		0.87		103		1.34		1.23		4.12		2.46	2.22
11.2	10	5.09	4.74	4.97	1.40		2.54	118.2		33.7	33.2	183.22	183.22	166.22	42.53	42.38	40.97	9.42	10.37	9.72	17.75		21		39.5		3.2		227.53		4.65		0.85		106		2.152		1.16		3.91		2.33	2.18
8.2	7	6.32	5.97	6.2	1.36		2.44	125.4		34.75	34.25	210.5	215.64	198.64	40.97	40.82	42.73	8.12	9.07	8.42	11.6		21		45.18		2.8		215.75		4.65		0.81		103		1.116		1.23		4.31		2.18	2.37
11.2	10	6.32	5.97	6.2	1.69		2.14	116.3		34.59	34.09	220.19	277.86	260.86	41.73	41.58	39.75	11.56	12.51	11.86	16.5		13		46		2.7		172.35		4.38		0.83		107		1.116		1.14		3.91		2.34	2.22
9.8	8.6	6.32	5.97	6.2	1.40		2.54	119.6		33.7	33.2	277.86	253.36	236.36	42.53	42.38	42.53	7.35	8.3	7.65	22		21		43.5		2.5		235.5		2.88		0.81		103		1.116		1.1		3.96		2.46	2.08
9.5	8.3	6.32	5.97	6.2	2.56		2.94	114.9		34.42	33.92	227.53	220.19	203.19	39.75	39.6	40.97	9.25	10.2	9.55	13.7		13		45.18		3.2		185.55		4.93		0.79		105		1.34		1.39		4.24		2.37	2.22
8.9	7.7	8.12	7.77	8	1.40		2.24	116.3		34.75	34.25	215.64	227.53	210.53	42.36	42.21	42.73	8.46	9.41	8.76	21.72		14		41.75		3.4		225.45		4.52		0.81		103		1.186		1.23		3.91		2.22	2.28
9.5	8.3	6.32	5.97	6.2	1.42		2.54	125.4		33.7	33.2	235.74	284.53	267.53	42.53	42.38	42.36	9.42	10.37	9.72	18		19		42.36		2.9		185.55		4.63		0.92		103		1.194		1.16		4.16		2.18	2.46
7.7	6.5	7.09	6.74	6.97	1.75		2.14	125.6		35.56	35.06	277.86	281.23	264.23	42.73	42.58	42.53	9.48	10.43	9.78	17		21		41.75		2.8		224.65		4.38		0.79		103		1.116		1.1		4.12		2.18	2.22
12.5	11.3	6.32	5.97	6.2	1.55		2.54	127.6		34.42	33.92	220.19	277.86	260.86	40.96	40.81	42.77	9.75	10.7	10.05	11.6		21		45.18		3.2		215.75		4.65		0.87		102		2.136		1.14		3.91		2.22	2.36
9.8	8.6	7.45	7.1	7.33	1.75		2.04	125.4		33.7	33.2	227.53	249.27	232.27	42.53	42.38	42.36	9.42	10.37	9.72	17.75		19		74.5		3.3		172.35		4.92		0.79		103		1.194		1.23		3.96		2.36	2.18
9.5	8.3	6.32	5.97	6.2	2.12		2.74	116.3		34.75	34.25	215.64	215.75	198.75	40.97	40.82	40.97	7.35	8.3	7.65	13.7		14		43.5		2.9		185.55		4.38		0.79		105		1.34		1.1		3.91		2.46	2.22
8.9	7.7	6.32	5.97	6.2	2.47		2.44	119.6		35.2	34.7	277.86	183.22	166.22	39.67	39.52	42.36	6.75	7.7	7.05	22		19		39.5		3.2		185.55		4.61		0.81		103		2.152		1.08		3.91		2.33	2.34
9.5	8.3	6.32	5.97	6.2	1.69		2.64	132.6		34.15	33.65	181.93	220.19	203.19	42.53	42.38	39.67	11.55	12.5	11.85	11.84		16		45.18		3.4		172.35		2.88		0.92		106		1.194		1.14		4.24		2.22	2.23
11.2	10	6.32	5.97	6.2	1.40		2.74	125.4		33.7	33.2	220.19	277.86	260.86	39.53	39.38	42.73	7.35	8.3	7.65	16.5		19		47.83		2.7		225.45		4.86		0.92		103		1.116		1.23		4.24		2.18	2.22
9.8	8.6	6.32	5.97	6.2	1.36		2.04	118.2		34.75	34.25	227.53	227.53	210.53	42.36	42.21	39.75	9.42	10.37	9.72	18		14		39.5		2.7		215.75		4.38		0.79		105		2.143		1.1		4.26		2.08	2.18
9.5	8.3	5.09	4.74	4.97	1.42		2.64	116.3		34.15	33.65	277.86	235.74	218.74	42.53	42.38	40.96	7.35	8.3	7.65	17.75		19		45.18		3.2		227.53		4.52		0.81		103		1.194		1.29		4.12		2.46	2.36
8.2	7	8.12	7.77	8	2.57		2.84	137.8		36.59	36.09	215.64	215.64	198.64	40.96	40.81	42.53	8.12	9.07	8.42	15.4		21		45.25		2.9		185.55		4.65		0.92		103		1.186		1.16		3.91		2.22	2.37
7.1	5.9	6.32	5.97	6.2	2.12		2.44	125.6		33.7	33.2	183.22	230.15	213.15	42.73	42.58	41.73	11.56	12.51	11.86	17		19		46		2.5		235.5		2.88		0.87		103		1.116		1.1		4.12		2.18	2.46
8.9	7.7	5.09	4.74	4.97	2.39		2.44	119.6		34.42	33.92	227.53	253.36	236.36	42.53	42.38	42.53	8.75	9.7	9.05	17.5		19		14.75		3.4		172.35		4.63		0.83		103		1.34		1.23		3.91		2.36	2.22
11.2	10	6.32	5.97	6.2	1.75		2.54	132.6		34.15	33.65	220.19	277.86	260.86	39.75	39.6	40.97	7.35	8.3	7.65	22		14		43.5		3.2		215.75		4.52		0.79		102		1.168		1.14		4.24		2.28	2.08
9.5	8.3	6.32	5.97	6.2	2.47		2.34	124.8		34.75	34.25	143.75	284.53	267.53	40.97	40.82	42.53	11.55	12.5	11.85	17.75		19		74.5		3.6		185.55		4.38		0.81		103		1.116		1.1		3.96		2.22	2.33
9.8	8.6	7.09	6.74	6.97	1.89		2.34	116.3		33.7	33.2	210.5	281.23	264.23	42.53	42.38	39.75	8.46	9.41	8.76	19.4		15		41.75		3.3		225.45		4.92		0.85		105		1.116		1.36		4.16		2.23	2.22
9.5	8.3	6.32	5.97	6.2	1.42		2.04	118.2		35.2	34.7	215.64	220.19	203.19	41.69	41.54	42.53	7.35	8.3	7.65	17.5		19		42.36		2.7		172.35		4.65		0.81		105		1.194		1.23		3.91		2.46	2.18
9.5	8.3	6.32	5.97	6.2	1.69		2.34	132.6		34.15	33.65	227.53	143.75	126.75	40.96	40.81	42.73	9.75	10.7	10.05	18		14		45.18		2.8		195.25		4.93		0.79		103		1.34		1.08		4.12		2.18	2.23
12.1	10.9	6.32	5.97	6.2	2.12		2.44	125.4		34.75	34.25	183.22	277.86	260.86	42.36	42.21	42.53	12.34	13.29	12.64	16.5		21		41.75		3.2		185.55		2.88		0.92		103		1.194		1.1		4.24		2.22	2.34
9.8	8.6	5.09	4.74	4.97	2.56		2.54	116.3		33.7	33.2	220.19	249.27	232.27	42.77	42.62	42.77	9.42	10.37	9.72	13.7		19		48.55		2.9		172.35		4.38		0.79		102		1.116		1.23		3.91		2.46	2.22
8.9	7.7	6.32	5.97	6.2	1.89		3.04	113.6		34.59	34.09	210.5	183.22	166.22	40.97	40.82	42.53	7.35	8.3	7.65	11.6		19		46		3.4		215.75		4.71		0.87		106		1.168		1.16		4.31		2.22	2.36
7.1	5.9	6.56	6.21	6.44	2.12		2.84	125.6		34.15	33.65	277.86	215.64	198.64	42.73	42.58	39.75	6.75	7.7	7.05	17.75		19		43.5		3.2		195.25		4.63		0.83		103		1.116		1.1		4.16		2.36	2.46
9.5	8.3	5.09	4.74	4.97	2.43		2.44	127.6		34.42	33.92	235.74	253.36	236.36	42.36	42.21	42.53	8.12	9.07	8.42	22		7		45.25		3.6		185.55		4.38		0.81		102		1.34		1.14		4.12		2.22	2.28
9.5	8.3	6.32	5.97	6.2	2.47		2.33	132.6		33.7	33.2	215.64	215.75	198.75	39.75	39.6	40.96	11.56	12.51	11.86	17		19		42.36		2.9		225.45		4.65		0.96		105		1.116		1.29		3.91		2.37	2.22
11.2	10	6.32	5.97	6.2	1.42		2.84	118.2		34.75	34.25	181.93	277.86	260.86	40.96	40.81	42.53	7.35	8.3	7.65	21.72		19		46.25		3.2		215.75		4.52		0.87		103		1.194		1.36		3.91		2.33	2.18
9.8	8.6	6.32	5.97	6.2	1.69		2.74	121.9		35.2	34.7	210.5	235.74	218.74	41.69	41.54	42.53	9.38	10.33	9.68	17.75		21		45.18		2.8		230.75		4.92		0.92		103		2.152		1.23		3.96		2.22	2.37
10.7	9.5	8.12	7.77	8	2.43		2.34	125.4		34.75	34.25	227.53	220.19	203.19	42.53	42.38	41.69	11.68	12.63	11.98	18		21		41.75		2.5		217.42		4.38		0.79		106		1.194		1.1		4.35		2.18	2.22
7.7	6.5	7.45	7.1	7.33	1.97		2.54	124.6		33.7	33.2	220.19	249.27	232.27	39.53	39.38	39.75	9.25	10.2	9.55	13.7		16		74.5		2.7		185.55		4.93		0.79		103		1.116		1.29		3.96		2.34	2.21
9.8	8.6	6.32	5.97	6.2	2.56		2.24	125.6		35.56	35.06	281.23	230.15	213.15	42.36	42.21	40.97	7.35	8.3	7.65	16.5		9		43.5		3.2		172.35		2.88		0.85		102		1.186		1.23		3.91		2.22	2.18
9.5	8.3	5.09	4.74	4.97	2.43		2.50	131.2		34.42	33.92	215.64	253.36	236.36	42.73	42.58	42.73	8.46	9.41	8.76	13.4		21		48.55		2.9		215.75		4.65		0.92		105		2.152		1.14		4.24		2.23	2.22
8.9	7.7	6.32	5.97	6.2	1.42	125.6	1.26	137.8		34.75	34.25	210.5	277.86	260.86	40.97	40.82	39.2	9.42	10.37	9.72	21.72		19		46		3.4		225.45		4.71		0.89		103		1.116		1.1		3.91		2.46	2.08
9.5	8.3	6.32	5.97	6.2	1.55	113.6	3.04	118.2		33.7	33.2	183.22	230.15	213.15	40.96	40.81	39.2	11.55	12.5	11.85	22		19		45.18		3.2		185.55		4.52		0.83		105		1.34		1.16		4.16		2.22	2.37
11.6	10.4	7.09	6.74	6.97	2.47	125.4	2.74	119.6		37.24	36.74	215.75	227.53	210.53	39.67	39.52	40.96	7.35	8.3	7.65	17.5		15		41.75		3.3		227.53		4.63		0.81		103		1.116		1.23		3.91		2.21	2.22
8.2	7	6.56	6.21	6.44	1.55	119.6	1.74	121.9		34.15	33.65	227.53	143.75	126.75	41.73	41.58	42.36	9.25	10.2	9.55	13.7		19		42.36		2.9		172.35		4.65		0.92		103		1.194		1.08		3.96		2.22	2.36
8.9	7.7	6.32	5.97	6.2	1.69	121.6	2.74	125.4		35.2	34.7	215.64	215.64	198.64	42.36	42.21	39.75	8.12	9.07	8.42	17		21		74.5		3.2		215.75		4.38		0.87		105		1.132		1.14		4.12		2.46	2.46
12.9	11.7	6.32	5.97	6.2	1.89	113.6	2.54	124.6		36.39	35.89	210.5	277.86	260.86	42.77	42.62	41.73	11.56	12.51	11.86	18		21		46		2.7		185.95		4.92		0.79		102		1.194		1.23		3.96		2.18	2.34
10.6	9.4	6.32	5.97	6.2	1.75	125.4	2.44	125.6		34.42	33.92	277.86	253.36	236.36	42.73	42.58	42.73	9.38	10.33	9.68	17.75		19		43.5		3.4		185.55		2.88		0.92		102		2.143		1.1		3.91		2.36	2.18
9.8	8.6	6.32	5.97	6.2	2.43	116.30	2.14	132.6		34.15	33.65	235.74	220.19	203.19	40.96	40.81	39.67	7.35	8.3	7.65	17.75		19		45.25		2.7		225.45		4.38		0.81		103		1.186		1.39		4.23		2.28	2.22
9.5	8.3	6.32	5.97	6.2	2.39	118.2	2.54	118.2		34.59	34.09	220.19	183.22	166.22	40.97	40.82	42.77	7.23	8.18	7.53	16.5		24		41.75		3.2		215.75		4.53		0.79		105		1.34		1.23		3.96		2.22	2.33
11.6	10.4	6.32	5.97	6.2	2.12	125.4	2.94	119.6		35.2	34.7	230.15	215.75	198.75	41.73	41.58	42.36	12.36	13.31	12.66	19.4		19		42.36		2.5		172.35		4.65		0.94		103		1.116		1.14		4.12		2.33	2.28
9.5	8.3	6.32	5.97	6.2	1.55	129.4	2.24	127.6		37.21	36.71	249.27	281.23	264.23	42.53	42.38	40.96	7.35	8.3	7.65	22		21		45.18		3.4		185.55		4.52		0.79		103		1.194		1.1		3.96		2.34	2.21
8.9	7.7	6.56	6.21	6.44	2.43	118.20	2.54	116.3		34.15	33.65	277.86	277.86	260.86	42.36	42.21	40.97	11.55	12.5	11.85	11.6		19		46		2.5		195.25		2.88		0.87		103		2.152		1.23		3.91		2.22	2.22
11.2	10	8.12	7.77	8	1.75	119.6	2.14	125.4		35.56	35.06	215.64	227.53	210.53	41.73	41.58	39.53	8.46	9.41	8.76	23.2		21		43.25		3.2		230.75		4.38		0.85		105		1.168		1.16		4.16		2.46	2.21
9.5	8.3	6.32	5.97	6.2	1.69	114.9	2.54	124.6		34.42	33.92	183.22	253.36	236.36	39.2	39.05	39.75	12.25	13.2	12.55	15.4		16		45.18		2.5		185.55		4.52		0.79		103		2.152		1.1		4.35		2.46	2.36
12.9	11.7	6.32	5.97	6.2	1.42	127.6	2.04	121.9		35.2	34.7	215.75	235.74	218.74	42.73	42.58	42.73	7.35	8.3	7.65	18		19		46		2.5		215.75		4.86		0.81		106		2.143		1.23		3.91		2.18	2.22
9.8	8.6	6.32	5.97	6.2	2.57	132.6	2.74	124.8		34.15	33.65	143.75	143.75	126.75	40.96	40.81	42.36	11.56	12.51	11.86	17.5		9		43.5		2.5		195.25		4.38		0.92		103		1.116		1.14		4.24		2.22	2.18
8.3	7.1	5.09	4.74	4.97	2.12	125.4	2.44	118.2		36.59	36.09	277.86	220.19	203.19	42.77	42.62	42.53	7.35	8.3	7.65	17		21		43.62		3.2		225.45		4.65		0.79		105		1.194		1.36		3.91		2.08	2.46
7.7	6.5	6.32	5.97	6.2	1.75	118.2	2.64	119.6		33.7	33.2	215.64	277.86	260.86	42.36	42.21	42.36	8.12	9.07	8.42	21.72		19		45.18		2.5		185.55		4.63		0.83		103		1.34		1.1		4.31		2.37	2.37
9.5	8.3	6.32	5.97	6.2	2.43	125.40	2.74	125.6		34.59	34.09	235.74	215.75	198.75	40.97	40.82	41.73	12.25	13.2	12.55	16.5		21		14.75		2.9		172.35		4.38		0.79		102		1.186		1.23		3.91		2.36	2.22
9.8	8.6	6.32	5.97	6.2	1.55	116.3	2.04	116.3		34.15	33.65	210.5	215.64	198.64	42.36	42.21	42.53	9.25	10.2	9.55	22		19		41.75		2.8		227.53		4.65		0.92		107		1.194		1.08		3.96		2.33	2.34
8.2	7	6.32	5.97	6.2	1.69	119.6	2.64	125.4		35.2	34.7	281.23	253.36	236.36	39.67	39.52	42.77	7.35	8.3	7.65	12.83		19		42.36		3.2		215.75		4.52		0.81		103		1.132		1.14		4.24		2.22	2.08
11.6	10.4	7.45	7.1	7.33	2.54	114.9	2.84	124.6		33.7	33.2	220.19	281.23	264.23	42.73	42.58	40.96	9.25	10.2	9.55	17.75		21		41.75		3.6		172.35		2.88		0.89		103		1.194		1.23		4.12		2.21	2.21
12.5	11.3	6.32	5.97	6.2	2.43	116.30	2.44	132.6		34.42	33.92	277.86	227.53	210.53	39.75	39.6	42.53	11.55	12.5	11.85	17.75		21		74.5		2.9		235.5		4.92		0.85		105		1.116		1.1		4.09		2.22	2.18
9.5	8.3	6.32	5.97	6.2	2.57	125.4	2.44	121.9		37.24	36.74	183.22	277.86	260.86	40.96	40.81	40.97	12.34	13.29	12.64	18		21		43.5		3.3		185.55		4.98		0.83		102		2.136		1.29		3.96		2.28	2.28
9.8	8.6	6.32	5.97	6.2	2.39	125.6	2.54	118.2		34.15	33.65	210.5	183.22	166.22	42.53	42.38	42.36	7.35	8.3	7.65	17.5		21		46		3.2		215.75		4.38		0.92		103		1.34		1.1		3.91		2.18	2.22
10.7	9.5	6.56	6.21	6.44	1.75	127.6	2.34	119.6		33.7	33.2	227.53	230.15	213.15	41.73	41.58	42.53	8.46	9.41	8.76	11.6		19		45.18		3.4		225.45		4.71		0.79		105		1.168		1.14		4.16		2.46	2.46
8.2	7	6.32	5.97	6.2	1.55	125.4	2.34	116.3		36.39	35.89	215.75	220.19	203.19	42.53	42.38	41.69	9.42	10.37	9.72	19.4		15		45.25		2.9		240.2		4.38		0.79		103		2.143		1.16		3.96		2.34	2.36
9.5	8.3	6.46	6.11	6.34	1.89	116.3	2.04	121.6		35.2	34.7	215.64	215.75	198.75	40.97	40.82	42.73	7.35	8.3	7.65	17		19		47.83		3.2		185.55		2.88		0.81		102		1.194		1.23		4.17		2.37	2.33
9.8	8.6	6.32	5.97	6.2	1.69	119.6	2.34	125.4		35.56	35.06	277.86	277.86	260.86	42.53	42.38	39.75	11.56	12.51	11.86	22		19		46.25		2.7		172.35		4.93		0.92		103		1.116		1.1		3.96		2.36	2.37
9.5	8.3	8.12	7.77	8	1.55	132.6	2.44	124.6		33.7	33.2	210.5	281.23	264.23	39.75	39.6	39.67	8.12	9.07	8.42	16.5		16		41.75		2.5		215.75		4.38		0.96		103		1.194		1.23		4.24		2.22	2.18
8.9	7.7	7.09	6.74	6.97	2.43	125.40	2.54	137.8		34.42	33.92	230.15	235.74	218.74	42.53	42.38	42.77	9.42	10.37	9.72	11.84		21		47.83		2.5		185.95		4.52		0.92		103		2.152		1.39		3.91		2.33	2.22
12.1	10.9	6.46	6.11	6.34	2.47	118.2	3.04	125.6		34.59	34.09	284.53	227.53	210.53	42.73	42.58	42.36	7.35	8.3	7.65	13.4		21		43.5		3.2		172.35		4.92		0.79		103		1.116		1.14		4.31		2.18	2.36
12.9	11.7	7.45	7.1	7.33	2.43	116.30	2.84	118.2		34.75	34.25	220.19	215.64	198.64	42.53	42.38	42.53	11.55	12.5	11.85	18		21		41.75		2.7		185.55		4.38		0.79		103		1.34		1.1		3.96		2.21	2.34
9.8	8.6	6.46	6.11	6.34	2.12	137.8	2.44	119.6		33.7	33.2	235.74	277.86	260.86	42.77	42.62	41.73	9.42	10.37	9.72	17.75		19		45.18		2.9		225.45		4.65		0.83		103		1.132		1.23		4.16		2.23	2.23
12.5	11.3	6.32	5.97	6.2	1.42	125.6	2.33	116.3		34.15	33.65	210.5	253.36	236.36	42.53	42.38	40.96	11.68	12.63	11.98	15.4		15		46		2.7		215.75		4.63		0.81		102		1.116		1.08		4.24		2.22	2.46
9.5	8.3	5.09	4.74	4.97	2.21	119.6	2.84	121.9		35.2	34.7	277.86	143.75	126.75	39.75	39.6	40.97	11.46	12.41	11.76	21.72		19		74.5		3.2		224.65		4.65		0.92		105		1.186		1.16		4.12		2.37	2.23
8.2	7	6.32	5.97	6.2	1.75	132.6	2.74	125.4		37.49	36.99	215.75	220.19	203.19	42.53	42.38	42.73	7.35	8.3	7.65	18		21		48.55		2.8		227.53		2.88		0.85		103		1.194		1.1		3.91		2.28	2.18
12.1	10.9	6.46	6.11	6.34	1.69	124.8	2.34	124.6		33.7	33.2	227.53	183.22	166.22	40.96	40.81	42.36	9.25	10.2	9.55	17.75		21		47.83		3.4		225.45		4.38		0.89		105		1.34		1.14		3.96		2.22	2.22
9.8	8.6	6.46	6.11	6.34	2.56	116.3	1.16	131.2		36.59	36.09	215.64	215.75	198.75	42.53	42.38	42.36	8.12	9.07	8.42	22		13		43.5		2.9		185.55		4.92		0.92		103		1.116		1.23		4.23		2.46	2.21
9.5	8.3	6.46	6.11	6.34	2.43	118.20	1.18	132.6		34.15	33.65	210.5	277.86	260.86	42.53	42.38	42.53	11.55	12.5	11.85	17.5		21		41.75		3.4		215.75		4.61		0.83		103		1.168		1.23		4.09		2.18	2.28
12.9	11.7	6.46	6.11	6.34	1.89	132.6	1.33	118.2		35.2	34.7	183.22	253.36	236.36	41.69	41.54	39.75	7.35	8.3	7.65	17		21		43.62		3.6		195.25		2.88		0.81		102		1.194		1.23		4.24		2.22	2.18
9.5	8.3	6.46	6.11	6.34	1.55	125.4	1.25	119.6		33.7	33.2	277.86	253.36	236.36	39.75	39.6	39.2	8.46	9.41	8.76	18		21		45.18		3.2		235.5		4.38		0.79		106		1.116		1.1		3.96		2.36	2.36
8.9	7.7	6.32	5.97	6.2	1.78	116.3	1.16	116.3		37.24	36.74	230.15	183.22	166.22	40.97	40.82	42.36	9.42	10.37	9.72	16.5		13		45.25		3.3		172.35		4.52		0.92		103		1.34		1.29		3.91		2.34	2.37
8.2	7	6.46	6.11	6.34	2.39	113.6	1.14	121.9		34.59	34.09	281.23	235.74	218.74	42.73	42.58	42.73	8.12	9.07	8.42	18		13		46		3.4		172.35		4.86		0.87		103		2.143		1.18		4.16		2.22	2.22
8.3	7.1	8.12	7.77	8	2.63	125.6	1.26	124.6		34.15	33.65	210.5	215.64	198.64	39.2	39.05	40.97	7.35	8.3	7.65	11.6		13		41.75		3.2		185.55		4.65		0.79		102		1.186		1.1		4.12		2.21	2.46
10.7	9.5	6.46	6.11	6.34	1.69	127.6	1.28	125.4		33.7	33.2	220.19	220.19	203.19	39.2	39.05	42.36	11.56	12.51	11.86	13.7		16		43.5		2.5		215.75		4.92		0.85		103		1.116		1.23		4.24		2.36	2.34
8.9	7.7	6.46	6.11	6.34	2.12	132.6	1.33	125.6		35.2	34.7	215.75	227.53	210.53	40.96	40.81	39.75	11.46	12.41	11.76	17.75		21		74.5		2.5		230.75		4.38		0.81		103		2.152		1.14		3.96		2.46	2.36
9.5	8.3	6.46	6.11	6.34	1.89	118.2	1.18	121.6		37.49	36.99	277.86	277.86	260.86	42.36	42.21	39.2	11.55	12.5	11.85	19.4		21		42.36		2.9		225.45		4.68		0.83		105		1.194		1.16		3.96		2.18	2.33
9.8	8.6	8.12	7.77	8	2.21	121.9	1.22	118.2		35.56	35.06	215.64	253.36	236.36	39.75	39.6	39.67	7.35	8.3	7.65	22		19		45.18		3.2		217.42		4.38		0.92		103		1.168		1.23		3.91		2.22	2.22
11.6	10.4	6.32	5.97	6.2	1.55	125.4	1.25	119.6		33.7	33.2	210.5	220.19	203.19	41.73	41.58	42.53	8.12	9.07	#FIELD!	12.83		9		47.83		3.2		172.35		4.63		0.79		103		1.194		1.23		4.19		2.21	2.18
9.5	8.3	5.09	4.74	4.97	1.42	124.6	1.25	137.8		35.2	34.7	235.74	210.5	193.5	42.73	42.58	41.73	12.36	13.31	12.66	17.5		17		46.25		2.7		215.75		4.52		0.94		105		1.34		1.1		4.31		2.33	2.21
12.1	10.9	6.46	6.11	6.34	2.47	125.6	1.26	116.3		37.49	36.99	249.27	215.64	198.64	39.67	39.52	42.53	9.42	10.37	9.72	18		21		41.75		2.6		225.45		4.65		0.87		103		1.116		1.08		3.91		2.22	2.08
8.9	7.7	6.46	6.11	6.34	2.43	131.20	1.31	124.6		36.59	36.09	183.22	227.53	210.53	42.77	42.62	42.73	7.23	8.18	7.53	13.7		21		46		2.8		185.55		4.71		0.79		102		1.186		1.14		4.24		2.18	2.46
9.5	8.3	6.46	6.11	6.34	1.69	137.8	1.38	121.9		35.2	34.7	277.86	277.86	260.86	42.36	42.21	42.36	9.48	10.43	9.78	17		13		43.5		3.2		215.75		4.38		0.83		103		1.132		1.1		3.96		2.37	2.37
7.7	6.5	6.46	6.11	6.34	2.57	118.2	1.18	125.4		34.15	33.65	210.5	210.5	193.5	40.96	40.81	40.96	7.35	8.3	7.65	16.5		13		74.5		2.9		227.53		4.92		0.81		103		1.116		1.19		4.12		2.22	2.22
10.7	9.5	6.56	6.21	6.44	2.39	119.6	1.20	124.8		37.24	36.74	227.53	235.74	218.74	40.97	40.82	40.97	8.12	9.07	8.42	17.75		15		47.83		2.7		217.42		4.63		0.92		105		1.116		1.23		3.96		2.08	2.28
9.8	8.6	5.09	4.74	4.97	2.43	121.90	1.22	118.2		34.42	33.92	143.75	220.19	203.19	39.53	39.38	39.75	8.46	9.41	8.76	23.2		21		43.25		3.2		185.55		4.65		0.92		103		2.143		1.1		4.35		2.22	2.18
9.5	8.3	5.09	4.74	4.97	1.89	125.4	1.25	119.6		37.49	36.99	220.19	227.53	210.53	39.75	39.6	42.77	11.56	12.51	11.86	22		16		45.25		2.9		225.45		2.88		0.79		103		1.186		1.16		3.91		2.28	2.36
12.1	10.9	6.46	6.11	6.34	2.12	124.6	1.25	125.6		35.2	34.7	215.64	210.5	193.5	42.73	42.58	41.69	7.35	8.3	7.65	17.75		21		47.83		3.4		215.75		4.38		0.89		103		1.34		1.14		4.24		2.46	2.34
9.8	8.6	6.46	6.11	6.34	1.75	125.6	1.26	124.6		34.15	33.65	210.5	277.86	260.86	42.36	42.21	42.36	8.12	9.07	8.42	18		13		43.5		3.2		195.25		4.38		0.83		105		2.152		1.23		3.96		2.22	2.21
8.9	7.7	6.46	6.11	6.34	2.43	132.60	1.33	116.3		34.75	34.25	277.86	215.64	198.64	42.53	42.38	42.73	9.42	10.37	9.72	11.6		13		14.75		3.3		172.35		4.61		0.81		102		1.116		1.1		3.91		2.36	2.22
8.2	7	7.45	7.1	7.33	1.76	118.2	1.18	118.2		34.59	34.09	215.75	183.22	166.22	42.36	42.21	42.36	12.95	13.9	13.25	19.4		16		47.83		2.9		185.55		4.52		0.79		103		1.194		1.18		4.12		2.18	2.37
9.5	8.3	6.46	6.11	6.34	1.69	119.6	1.20	132.6		34.42	33.92	230.15	210.5	193.5	41.73	41.58	42.53	7.35	8.3	7.65	13.7		21		46		3.2		225.45		4.92		0.87		103		1.168		1.23		3.96		2.22	2.46
12.5	11.3	6.46	6.11	6.34	1.97	127.6	1.28	125.4		35.56	35.06	235.74	220.19	203.19	42.53	42.38	39.75	9.25	10.2	9.55	15.4		13		41.75		3.6		172.35		2.88		0.79		102		2.136		1.08		3.91		2.34	2.18
9.5	8.3	6.32	5.97	6.2	1.75	116.3	1.16	118.2		34.15	33.65	210.5	277.86	260.86	42.77	42.62	40.97	8.12	9.07	8.42	17		21		74.5		2.7		215.75		4.65		0.85		107		1.34		1.23		4.09		2.23	2.33
12.1	10.9	6.46	6.11	6.34	2.43	125.40	1.25	121.9		33.7	33.2	281.23	227.53	210.53	40.96	40.81	40.96	11.55	12.5	11.85	16.5		21		43.5		3.4		224.65		4.86		0.83		103		1.186		1.1		3.91		2.22	2.23
9.8	8.6	5.09	4.74	4.97	1.75	124.6	1.25	118.2		35.2	34.7	277.86	215.64	198.64	42.53	42.38	42.36	7.35	8.3	7.65	22		15		48.55		3.2		185.55		4.65		0.92		103		1.116		1.14		4.16		2.18	2.22
11.6	10.4	6.46	6.11	6.34	2.12	121.9	1.22	119.6		34.59	34.09	227.53	235.74	218.74	40.97	40.82	39.53	9.25	10.2	9.55	17.5		13		42.36		2.8		235.5		4.38		0.81		103		1.194		1.23		3.91		2.36	2.36
9.8	8.6	6.32	5.97	6.2	1.42	124.8	1.25	121.6		37.49	36.99	220.19	277.86	260.86	42.36	42.21	40.97	12.25	13.2	12.55	18		21		74.5		2.5		225.45		4.92		0.96		105		2.143		1.1		3.96		2.37	2.23
9.5	8.3	6.46	6.11	6.34	2.56	118.2	1.18	116.3		34.15	33.65	210.5	220.19	203.19	42.53	42.38	39.67	7.35	8.3	7.65	13.7		21		41.75		3.2		215.75		4.93		0.79		103		1.168		1.16		4.24		2.46	2.28
8.9	7.7	7.09	6.74	6.97	1.76	119.6	1.20	124.6		34.42	33.92	215.64	227.53	210.53	41.69	41.54	42.53	9.42	10.37	9.72	17.75		13		45.18		2.7		185.55		4.38		0.87		106		1.194		1.23		3.96		2.22	2.46
8.2	7	8.12	7.77	8	1.69	125.6	1.26	137.8		35.2	34.7	183.22	215.64	198.64	42.73	42.58	42.36	11.55	12.5	11.85	17.75		13		46		3.2		172.35		2.88		0.87		103		1.186		1.1		3.91		2.08	2.18
7.7	6.5	6.46	6.11	6.34	1.75	116.3	1.16	125.4		36.59	36.09	277.86	277.86	260.86	39.75	39.6	42.73	11.56	12.51	11.86	21.72		16		45.25		2.9		195.25		4.65		0.81		102		1.116		1.29		4.31		2.33	2.22
9.5	8.3	7.02	6.67	6.9	2.39	125.4	1.25	118.2		37.24	36.74	215.75	181.93	164.93	39.67	39.52	42.53	12.95	13.9	13.25	11.6		21		43.5		3.2		225.45		2.88		0.92		103		1.34		1.23		3.91		2.22	2.34
9.8	8.6	7.02	6.67	6.9	1.97	124.6	1.25	125.6		34.15	33.65	284.53	220.19	203.19	42.77	42.62	40.96	8.46	9.41	8.76	13.4		13		47.83		2.6		215.75		4.52		0.83		102		2.152		1.1		4.12		2.18	2.33
8.9	7.7	5.09	4.74	4.97	1.78	132.6	1.33	121.9		35.2	34.7	210.5	227.53	210.53	42.36	42.21	42.36	11.68	12.63	11.98	22		21		46.42		3.3		230.75		4.38		0.87		103		1.132		1.19		4.19		2.23	2.22
9.5	8.3	7.02	6.67	6.9	2.57	121.9	1.22	122.3		34.42	33.92	230.15	277.86	260.86	42.53	42.38	42.53	7.35	8.3	7.65	17		16		47.35		2.5		185.55		4.92		0.79		103		1.34		1.23		4.24		2.34	2.28
10.6	9.4	8.12	7.77	8	2.54	118.2	1.18	124.6		37.49	36.99	215.64	215.64	198.64	41.73	41.58	40.97	11.46	12.41	11.76	18		21		74.5		3.4		172.35		4.63		0.92		102		1.168		1.08		3.96		2.22	2.08
9.5	8.3	7.02	6.67	6.9	1.89	119.6	1.20	116.3		33.7	33.2	277.86	183.22	166.22	40.96	40.81	39.75	9.25	10.2	9.55	16.5		13		45.18		2.7		215.75		4.53		0.81		103		1.116		1.1		4.23		2.36	2.36
11.2	10	7.02	6.67	6.9	1.69	116.3	1.16	132.6		34.15	33.65	220.19	227.53	210.53	40.97	40.82	42.53	11.96	12.91	12.26	11.84		21		14.75		2.7		225.45		4.65		0.89		105		1.132		1.23		3.91		2.46	2.18
9.8	8.6	7.02	6.67	6.9	1.76	121.6	1.22	121.9		37.22	36.72	227.53	220.19	203.19	42.73	42.58	42.73	11.55	12.5	11.85	13.4		13		46		2.5		185.55		2.88		0.79		103		1.34		1.14		3.96		2.28	2.46
9.5	8.3	6.32	5.97	6.2	2.43	125.40	1.25	125.4		34.59	34.09	235.74	143.75	126.75	42.36	42.21	42.36	11.56	12.51	11.86	21.72		21		74.5		3.2		227.53		4.38		0.92		102		2.143		1.29		4.31		2.22	2.22
9.8	8.6	7.02	6.67	6.9	1.42	124.6	1.25	118.2		33.7	33.2	210.5	210.5	193.5	42.36	42.21	42.53	7.35	8.3	7.65	17.5		13		41.75		2.8		215.75		4.92		0.87		103		1.186		1.23		4.12		2.18	2.37
7.7	6.5	7.02	6.67	6.9	1.75	137.8	1.38	125.6		37.21	36.71	284.53	215.64	198.64	42.53	42.38	42.77	8.46	9.41	8.76	17.75		9		45.25		2.7		195.25		4.86		0.83		105		1.194		1.1		4.24		2.37	2.33
9.5	8.3	5.09	4.74	4.97	2.47	125.6	1.26	131.2		34.15	33.65	277.86	227.53	210.53	39.75	39.6	40.96	12.95	13.9	13.25	22		13		42.36		2.5		185.55		4.38		0.81		103		1.116		1.16		3.91		2.22	2.34
8.9	7.7	6.32	5.97	6.2	2.56	118.2	1.18	124.6		34.42	33.92	183.22	183.22	166.22	39.2	39.05	42.53	11.68	12.63	11.98	18		21		47.83		3.2		172.35		4.52		0.79		102		2.152		1.23		4.09		2.21	2.28
8.2	7	7.02	6.67	6.9	2.39	119.6	1.20	121.6		33.7	33.2	215.75	220.19	203.19	42.36	42.21	40.97	12.25	13.2	12.55	17.75		21		45.18		3.4		240.2		4.71		0.83		103		2.136		1.08		3.91		2.18	2.18
9.8	8.6	7.02	6.67	6.9	2.21	116.3	1.16	116.3		34.75	34.25	215.64	210.5	193.5	42.73	42.58	41.73	9.42	10.37	9.72	15.4		16		43.25		3.2		185.55		2.88		0.94		103		1.34		1.1		4.17		2.22	2.22
8.2	7	7.02	6.67	6.9	1.69	121.9	1.22	119.6		37.24	36.74	220.19	277.86	260.86	40.97	40.82	42.53	12.36	13.31	12.66	11.6		13		46		2.9		225.45		4.63		0.87		103		2.143		1.23		4.24		2.08	2.21
11.2	10	8.12	7.77	8	1.75	125.4	1.25	121.9		34.15	33.65	210.5	235.74	218.74	42.36	42.21	39.75	7.35	8.3	7.65	16.5		24		48.55		3.3		215.75		4.92		0.83		103		1.194		1.14		4.12		2.37	2.46
12.9	11.7	7.02	6.67	6.9	2.43	124.60	1.25	125.4		34.75	34.25	277.86	215.64	198.64	39.75	39.6	42.36	11.68	12.63	11.98	17		21		46.25		3.4		217.42		4.38		0.92		106		1.116		1.16		3.91		2.22	2.36
9.5	8.3	7.02	6.67	6.9	1.89	131.2	1.31	118.2		35.56	35.06	281.23	181.93	164.93	39.2	39.05	42.53	12.34	13.29	12.64	13.95		21		41.75		3.6		235.5		4.65		0.81		103		1.186		1.23		3.96		2.36	2.23
9.8	8.6	7.02	6.67	6.9	2.57	132.6	1.33	125.6		34.42	33.92	227.53	210.5	193.5	39.67	39.52	42.73	11.56	12.51	11.86	23.2		13		42.38		3.2		195.25		4.92		0.79		103		1.132		1.1		3.91		2.46	2.37
12.1	10.9	6.56	6.21	6.44	1.76	118.2	1.18	122.3		36.59	36.09	143.75	227.53	210.53	42.53	42.38	40.96	7.35	8.3	7.65	22		15		45.25		2.7		215.75		4.52		0.85		105		1.194		1.39		4.16		2.34	2.22
8.3	7.1	6.32	5.97	6.2	2.21	119.6	1.20	124.6		34.15	33.65	230.15	220.19	203.19	41.73	41.58	42.53	8.46	9.41	8.76	19.4		13		45.18		3.4		215.75		4.93		0.96		103		1.34		1.23		4.24		2.18	2.18
9.8	8.6	5.09	4.74	4.97	1.75	116.3	1.16	119.6		37.22	36.72	277.86	281.23	264.23	42.53	42.38	40.97	8.12	9.07	8.42	17.75		21		14.75		2.9		185.55		4.38		0.87		105		2.143		1.36		4.35		2.22	2.08
12.9	11.7	7.02	6.67	6.9	2.39	121.9	1.22	116.3		34.59	34.09	210.5	215.64	198.64	42.73	42.58	39.67	9.42	10.37	9.72	21.72		13		46		3.2		225.45		2.88		0.79		103		1.116		1.1		4.31		2.33	2.34
11.6	10.4	7.02	6.67	6.9	2.43	124.60	1.25	132.6		36.39	35.89	235.74	210.5	193.5	42.36	42.21	42.53	9.25	10.2	9.55	18		21		74.5		2.9		172.35		4.63		0.89		102		1.168		1.23		4.24		2.28	2.46
8.2	7	6.32	5.97	6.2	1.69	125.4	1.25	137.8		34.42	33.92	215.64	183.22	166.22	40.96	40.81	39.53	11.55	12.5	11.85	13.95		13		47.83		2.8		172.35		4.38		0.81		103		1.194		1.14		4.12		2.21	2.21
9.5	8.3	7.02	6.67	6.9	1.42	125.6	1.26	125.4		33.7	33.2	220.19	215.75	198.75	40.97	40.82	42.36	11.55	12.5	11.85	17.75		13		42.36		3.3		215.75		4.92		0.92		103		2.152		1.08		3.91		2.22	2.22
10.7	9.5	5.09	4.74	4.97	2.47	121.6	1.22	125.6		37.21	36.71	183.22	227.53	210.53	39.75	39.6	42.53	7.35	8.3	7.65	16.5		21		41.75		2.7		185.95		4.65		0.79		103		1.186		1.23		4.19		2.21	2.36
8.2	7	7.02	6.67	6.9	1.75	118.2	1.18	121.9		35.2	34.7	215.75	215.64	198.64	42.77	42.62	40.96	8.12	9.07	8.42	13.4		9		43.62		3.2		215.75		2.88		0.85		103		1.34		1.16		4.23		2.36	2.18
7.7	6.5	7.02	6.67	6.9	1.89	119.6	1.20	124.6		35.56	35.06	227.53	210.5	193.5	41.69	41.54	42.73	7.35	8.3	7.65	22		21		45.18		2.7		185.55		4.98		0.83		102		1.116		1.1		4.24		2.22	2.28
11.2	10	7.02	6.67	6.9	2.12	137.8	1.38	131.2		36.59	36.09	210.5	277.86	260.86	42.36	42.21	42.53	12.95	13.9	13.25	17		16		48.55		2.5		225.45		4.92		0.92		103		1.34		1.23		4.16		2.18	2.33
7.1	5.9	6.46	6.11	6.34	1.75	116.3	1.16	122.3		34.15	33.65	249.27	235.74	218.74	42.73	42.58	39.75	11.46	12.41	11.76	11.6		13		43.5		3.4		185.55		2.88		0.94		103		1.194		1.39		4.24		2.46	2.28
12.5	11.3	4.42	4.07	4.3	2.43	124.60	1.25	116.3		34.42	33.92	281.23	220.19	203.19	42.36	42.21	40.97	9.42	10.37	9.72	18		21		46		3.2		185.55		4.38		0.81		105		2.143		1.18		3.91		2.37	2.22
9.8	8.6	6.32	5.97	6.2	1.58	121.9	1.22	125.6		35.2	34.7	215.64	230.15	213.15	42.53	42.38	42.53	12.25	13.2	12.55	17.5		21		45.25		3.6		215.75		4.61		0.89		106		1.186		1.23		4.12		2.22	2.46
8.9	7.7	4.42	4.07	4.3	1.76	125.4	1.25	125.4		37.22	36.72	230.15	249.27	232.27	39.75	39.6	41.69	8.12	9.07	8.42	17.75		7		46.42		2.9		230.75		4.52		0.79		103		1.194		1.1		4.31		2.34	2.37
12.9	11.7	4.42	4.07	4.3	1.69	124.8	2.64	118.2		34.59	34.09	183.22	277.86	260.86	40.97	40.82	40.96	7.35	8.3	7.65	13.7		13		46.25		3.2		195.25		4.38		0.92		102		1.116		1.14		4.35		2.08	2.18
11.2	10	4.42	4.07	4.3	2.57	118.2	2.64	121.9		34.15	33.65	235.74	215.64	198.64	40.96	40.81	42.36	11.56	12.51	11.86	15.4		21		43.5		3.6		172.35		2.88		0.87		103		1.132		1.23		4.16		2.21	2.34
9.5	8.3	8.12	7.77	8	2.56	119.6	2.84	124.6		35.2	34.7	253.36	183.22	166.22	42.36	42.21	42.77	8.46	9.41	8.76	21.72		15		45.18		3.4		224.65		4.92		0.85		103		1.34		1.1		3.91		2.18	2.36
9.8	8.6	4.42	4.07	4.3	1.97	125.6	2.84	132.6		34.42	33.92	227.53	215.75	198.75	39.53	39.38	40.97	9.25	10.2	9.55	16.5		16		14.75		2.5		185.55		4.93		0.92		102		2.152		1.16		4.24		2.28	2.22
8.2	7	4.42	4.07	4.3	2.43	124.60	2.44	121.6		33.7	33.2	215.75	143.75	126.75	42.73	42.58	42.73	7.35	8.3	7.65	23.2		13		41.75		3.3		225.45		4.63		0.81		107		1.194		1.23		3.91		2.22	2.18
8.3	7.1	5.09	4.74	4.97	1.89	116.3	2.54	118.2		34.75	34.25	277.86	277.86	260.86	39.75	39.6	42.36	11.55	12.5	11.85	18		21		47.83		2.7		172.35		4.86		0.79		103		2.143		1.08		4.12		2.46	2.23
11.2	10	4.42	4.07	4.3	2.12	118.2	2.54	116.3		34.15	33.65	220.19	215.64	198.64	41.69	41.54	39.75	9.42	10.37	9.72	13.95		18		46		3.2		185.55		4.92		0.79		105		1.116		1.1		3.91		2.36	2.22
12.9	11.7	4.42	4.07	4.3	1.42	132.6	2.34	125.4		37.22	36.72	284.53	235.74	218.74	42.77	42.62	40.96	12.95	13.9	13.25	17		21		47.35		2.7		215.75		2.88		0.83		102		2.152		1.23		4.09		2.33	2.08
8.2	7	4.42	4.07	4.3	1.75	125.4	2.54	125.6		33.7	33.2	215.64	210.5	193.5	42.36	42.21	41.69	11.46	12.41	11.76	17.75		9		43.5		2.5		227.53		4.38		0.96		103		1.194		1.14		3.91		2.37	2.28
9.5	8.3	4.42	4.07	4.3	1.69	118.2	2.84	127.6		37.21	36.71	253.36	281.23	264.23	41.73	41.58	42.53	9.25	10.2	9.55	17.75		18		42.36		3.4		172.35		4.65		0.83		105		1.34		1.1		4.16		2.18	2.46
7.1	5.9	4.42	4.07	4.3	1.76	121.9	2.94	121.9		34.75	34.25	143.75	220.19	203.19	42.73	42.58	39.53	7.35	8.3	7.65	11.6		21		45.18		2.8		172.35		4.38		0.94		103		1.186		1.23		4.24		2.22	2.37
7.7	6.5	7.45	7.1	7.33	2.35	118.2	2.34	132.6		34.15	33.65	277.86	277.86	260.86	40.96	40.81	42.36	12.25	13.2	12.55	21.72		16		45.25		2.9		195.25		4.93		0.81		103		2.152		1.29		4.31		2.36	2.18
10.6	9.4	4.42	4.07	4.3	2.43	119.60	2.54	118.2		34.42	33.92	281.23	183.22	166.22	42.53	42.38	42.73	7.35	8.3	7.65	19.4		21		47.83		3.6		185.55		4.92		0.87		103		1.116		1.19		4.24		2.34	2.22
11.2	10	5.09	4.74	4.97	2.12	121.6	2.44	124.6		35.2	34.7	183.22	210.5	193.5	39.67	39.52	40.97	11.56	12.51	11.86	16.5		18		74.5		3.2		215.75		4.52		0.79		102		1.194		1.23		4.12		2.23	2.21
8.2	7	4.42	4.07	4.3	1.89	116.3	2.24	125.6		35.56	35.06	227.53	227.53	210.53	42.36	42.21	40.96	9.42	10.37	9.72	13.7		18		41.75		3.3		225.45		4.63		0.92		105		1.132		1.1		3.91		2.46	2.34
9.5	8.3	4.42	4.07	4.3	2.47	124.6	2.24	116.3		36.39	35.89	253.36	215.75	198.75	42.77	42.62	39.67	8.46	9.41	8.76	17.5		18		43.5		2.5		235.5		4.71		0.85		103		2.152		1.14		4.09		2.23	2.36
10.7	9.5	4.42	4.07	4.3	2.57	137.8	2.34	125.4		34.15	33.65	220.19	215.64	198.64	39.75	39.6	41.73	11.55	12.5	11.85	13.4		21		46		2.9		172.35		4.65		0.83		103		2.152		1.23		4.23		2.18	2.28
9.8	8.6	4.42	4.07	4.3	1.75	125.4	2.94	137.8		35.2	34.7	277.86	277.86	260.86	39.2	39.05	42.36	12.95	13.9	13.25	17		7		46.25		2.9		215.75		4.38		0.79		105		1.34		1.16		4.31		2.22	2.46
8.2	7	4.42	4.07	4.3	1.76	118.2	2.04	119.6		37.25	36.75	215.75	210.5	193.5	42.73	42.58	42.77	8.12	9.07	8.42	17.75		21		45.18		3.2		185.55		4.31		0.81		106		1.116		1.1		4.24		2.21	2.33
9.5	8.3	6.32	5.97	6.2	2.43	125.60	2.44	122.3		34.59	34.09	230.15	230.15	213.15	39.53	39.38	42.73	7.35	8.3	7.65	15.4		18		43.62		3.4		225.45		2.88		0.92		103		1.194		1.23		4.35		2.28	2.22
8.9	7.7	7.09	6.74	6.97	1.97	121.9	3.14	121.9		35.2	34.7	215.64	284.53	267.53	42.36	42.21	40.96	12.36	13.31	12.66	17.75		16		47.83		2.7		172.35		4.63		0.79		102		1.168		1.08		3.91		2.18	2.18
12.5	11.3	5.09	4.74	4.97	1.42	122.3	2.34	127.6		33.7	33.2	253.36	220.19	203.19	40.97	40.82	40.97	12.34	13.29	12.64	11.84		9		48.55		3.2		185.55		4.92		0.89		103		1.186		1.39		4.12		2.36	2.08
12.9	11.7	4.42	4.07	4.3	1.89	124.6	2.20	125.6		37.21	36.71	235.74	235.74	218.74	42.77	42.62	41.73	9.42	10.37	9.72	22		21		14.75		2.9		185.95		4.93		0.83		103		1.132		1.23		3.91		2.37	2.28
8.3	7.1	4.42	4.07	4.3	2.39	116.3	2.10	118.2		35.56	35.06	277.86	210.5	193.5	41.69	41.54	42.53	11.55	12.5	11.85	16.5		18		43.5		2.8		215.75		4.63		0.87		103		1.194		1.14		4.24		2.22	2.37
8.9	7.7	8.12	7.77	8	2.56	132.6	1.90	116.3		35.2	34.7	249.27	277.86	260.86	41.73	41.58	42.36	12.25	13.2	12.55	23.2		16		45.25		3.6		185.55		4.38		0.92		105		1.116		1.1		4.17		2.46	2.36
9.5	8.3	8.12	7.77	8	2.21	121.9	1.80	125.4		33.7	33.2	230.15	215.75	198.75	40.96	40.81	41.73	11.56	12.51	11.86	21.72		21		46		3.2		195.25		4.65		0.81		103		2.152		1.23		3.91		2.34	2.22
11.2	10	4.42	4.07	4.3	2.47	125.4	2.40	119.6		34.15	33.65	220.19	227.53	210.53	42.36	42.21	39.2	7.35	8.3	7.65	13.7		21		45.18		2.8		172.35		4.52		0.96		103		1.34		1.29		4.16		2.36	2.46
9.5	8.3	4.42	4.07	4.3	2.43	118.20	2.00	124.6		34.75	34.25	253.36	215.64	198.64	42.73	42.58	42.73	8.46	9.41	8.76	18		21		41.75		2.5		215.75		2.88		0.79		102		2.143		1.1		4.24		2.33	2.18
12.9	11.7	4.42	4.07	4.3	1.76	125.6	2.00	124.8		37.22	36.72	227.53	210.5	193.5	39.75	39.6	40.96	12.95	13.9	13.25	12.83		21		43.25		2.9		185.55		4.38		0.92		105		1.194		1.23		4.31		2.22	2.21
7.7	6.5	5.09	4.74	4.97	1.78	131.2	2.00	121.9		37.24	36.74	277.86	183.22	166.22	40.97	40.82	42.36	7.35	8.3	7.65	17.75		18		42.36		2.7		225.45		4.53		0.89		103		1.186		1.16		3.91		2.18	2.34
11.6	10.4	4.42	4.07	4.3	1.75	124.6	2.20	118.2		36.59	36.09	183.22	277.86	260.86	39.2	39.05	41.73	12.25	13.2	12.55	17		15		46.25		3.3		230.75		4.71		0.92		103		1.116		1.14		4.12		2.21	2.37
12.9	11.7	4.42	4.07	4.3	1.89	121.6	2.00	121.6		34.15	33.65	215.75	230.15	213.15	42.36	42.21	42.53	9.42	10.37	9.72	22		18		43.5		2.5		215.75		2.88		0.79		102		1.168		1.23		4.35		2.08	2.22
9.8	8.6	4.42	4.07	4.3	1.69	116.3	1.70	119.6		34.42	33.92	215.64	281.23	264.23	39.75	39.6	42.77	9.25	10.2	9.55	11.6		21		74.5		3.2		185.55		4.93		0.81		103		1.186		1.1		4.31		2.46	2.28
8.2	7	4.42	4.07	4.3	2.21	119.6	2.00	125.4		35.2	34.7	253.36	210.5	193.5	39.67	39.52	40.96	11.68	12.63	11.98	16.5		16		47.83		3.4		240.2		4.38		0.83		105		1.116		1.08		4.23		2.37	2.33
12.9	11.7	4.42	4.07	4.3	2.35	121.9	2.10	116.3		34.59	34.09	281.23	220.19	203.19	41.69	41.54	42.53	11.55	12.5	11.85	13.4		21		45.18		3.2		215.75		4.65		0.92		107		2.152		1.23		4.24		2.22	2.34
8.3	7.1	4.42	4.07	4.3	2.43	125.40	2.10	127.6		37.49	36.99	277.86	215.75	198.75	42.73	42.58	40.97	7.35	8.3	7.65	17.75		18		46		2.9		172.35		4.63		0.79		103		1.34		1.16		4.31		2.28	2.18
12.1	10.9	5.09	4.74	4.97	1.42	118.2	2.40	125.6		34.15	33.65	230.15	277.86	260.86	42.36	42.21	42.36	12.95	13.9	13.25	17.5		21		48.55		3.2		225.45		2.88		0.94		102		1.116		1.1		4.12		2.18	2.46
10.7	9.5	6.56	6.21	6.44	2.57	125.6	2.50	118.2		35.56	35.06	220.19	215.64	198.64	40.96	40.81	42.53	8.46	9.41	8.76	11.84		9		41.75		2.7		185.55		4.52		0.87		106		1.132		1.23		3.91		2.36	2.22
12.9	11.7	4.42	4.07	4.3	1.97	122.3	2.10	121.9		33.7	33.2	227.53	210.5	193.5	40.97	40.82	41.69	11.56	12.51	11.86	19.4		18		45.25		2.5		195.25		4.86		0.89		103		1.194		1.14		4.16		2.34	2.23
7.7	6.5	6.46	6.11	6.34	1.76	124.6	1.90	124.6		34.42	33.92	143.75	235.74	218.74	42.77	42.62	42.73	7.35	8.3	7.65	22		16		43.5		2.7		172.35		4.38		0.81		103		2.143		1.36		4.24		2.21	2.22
11.6	10.4	6.32	5.97	6.2	1.75	119.6	2.20	132.6		35.2	34.7	253.36	249.27	232.27	39.75	39.6	39.75	9.42	10.37	9.72	17.75		21		47.83		3.2		227.53		4.93		0.92		103		1.132		1.23		4.12		2.22	2.46
9.5	8.3	6.46	6.11	6.34	1.69	116.3	2.10	125.4		33.7	33.2	277.86	183.22	166.22	42.36	42.21	39.67	12.95	13.9	13.25	17.5		21		43.25		3.4		185.55		2.88		0.83		103		1.186		1.18		4.19		2.37	2.22
8.9	7.7	8.12	7.77	8	2.47	132.6	2.40	122.3		37.49	36.99	235.74	277.86	260.86	41.69	41.54	42.77	11.55	12.5	11.85	16.5		21		45.18		3.4		185.95		4.65		0.85		105		1.116		1.08		4.24		2.46	2.18
11.2	10	8.12	7.77	8	2.43	137.80	1.80	121.9		37.24	36.74	215.75	210.5	193.5	42.53	42.38	42.36	12.34	13.29	12.64	17		18		14.75		3.6		215.75		4.38		0.79		102		2.152		1.23		3.91		2.18	2.36
8.2	7	8.12	7.77	8	1.78	125.4	2.20	116.3		33.7	33.2	183.22	227.53	210.53	42.77	42.62	42.53	12.25	13.2	12.55	13.7		15		46		3.2		225.45		4.52		0.92		103		1.34		1.16		4.24		2.33	2.28
8.9	7.7	6.46	6.11	6.34	2.39	125.6	3.05	125.6		36.39	35.89	215.64	143.75	126.75	42.73	42.58	41.73	8.12	9.07	8.42	11.6		17		48.55		2.9		235.5		4.61		0.92		105		2.143		1.1		4.12		2.23	2.22
9.8	8.6	6.46	6.11	6.34	2.12	121.9	1.90	137.8		34.15	33.65	277.86	220.19	203.19	41.73	41.58	40.96	12.95	13.9	13.25	23.2		21		47.83		2.8		172.35		4.52		0.79		102		2.136		1.23		4.26		2.22	2.23
10.6	9.4	6.46	6.11	6.34	1.42	124.6	1.90	127.6		33.7	33.2	253.36	215.64	198.64	42.36	42.21	40.97	9.42	10.37	9.72	22		18		43.5		3.3		215.75		2.88		0.89		103		1.194		1.29		4.12		2.36	2.46
12.9	11.7	6.46	6.11	6.34	1.76	131.2	2.00	118.2		34.42	33.92	220.19	210.5	193.5	40.97	40.82	42.73	9.25	10.2	9.55	18		15		41.75		2.5		185.55		4.63		0.87		106		1.116		1.08		3.91		2.23	2.18
12.9	11.7	6.46	6.11	6.34	2.54	122.3	2.10	125.4		35.2	34.7	227.53	277.86	260.86	39.2	39.05	42.36	7.35	8.3	7.65	17.75		18		42.36		3.4		195.25		2.88		0.81		103		1.186		1.23		4.09		2.28	2.22
8.9	7.7	6.46	6.11	6.34	1.69	116.3	2.00	124.6		33.7	33.2	284.53	215.75	198.75	40.96	40.81	42.36	8.46	9.41	8.76	15.4		18		45.18		2.7		215.75		4.98		0.79		103		1.168		1.14		4.24		2.46	2.46
9.5	8.3	6.46	6.11	6.34	2.43	125.60	2.30	124.8		34.15	33.65	281.23	230.15	213.15	39.53	39.38	42.53	11.56	12.51	11.86	13.4		21		47.83		3.2		172.35		4.38		0.79		103		1.132		1.39		4.31		2.18	2.22
8.2	7	7.09	6.74	6.97	2.56	125.4	2.30	121.9		34.59	34.09	277.86	235.74	218.74	42.36	42.21	39.75	9.48	10.43	9.78	18		21		42.38		3.6		230.75		4.65		0.92		105		1.34		1.23		4.12		2.22	2.36
9.8	8.6	6.46	6.11	6.34	1.65	118.2	1.70	125.6		33.7	33.2	249.27	210.5	193.5	42.77	42.62	39.2	12.95	13.9	13.25	16.5		16		46		2.7		215.75		4.63		0.83		103		2.152		1.36		3.91		2.34	2.22
9.5	8.3	5.09	4.74	4.97	1.89	121.9	2.10	122.3		36.39	35.89	215.75	281.23	264.23	39.75	39.6	42.36	11.55	12.5	11.85	17.75		9		45.25		2.9		185.55		4.38		0.92		103		1.116		1.1		4.19		2.33	2.37
11.6	10.4	6.32	5.97	6.2	2.21	124.6	2.00	118.2		35.56	35.06	183.22	277.86	260.86	39.67	39.52	42.73	9.42	10.37	9.72	13.95		18		43.5		3.2		225.45		4.52		0.81		102		1.186		1.23		4.24		2.22	2.33
8.3	7.1	6.46	6.11	6.34	2.47	132.6	2.10	127.6		33.7	33.2	220.19	227.53	210.53	40.97	40.82	40.97	9.25	10.2	9.55	22		18		47.83		3.4		227.53		4.53		0.92		103		1.194		1.08		4.16		2.28	2.22
7.7	6.5	7.45	7.1	7.33	2.57	121.6	1.90	125.4		34.42	33.92	277.86	220.19	203.19	42.36	42.21	42.36	12.95	13.9	13.25	17		16		47.35		2.9		215.75		2.88		0.79		106		2.143		1.16		4.35		2.08	2.18
9.5	8.3	8.12	7.77	8	2.35	118.2	2.50	119.6		35.2	34.7	227.53	210.5	193.5	41.69	41.54	39.75	7.35	8.3	7.65	17.5		21		45.18		3.2		172.35		4.63		0.89		102		1.168		1.23		3.91		2.36	2.34
8.2	7	6.46	6.11	6.34	2.43	116.30	2.00	132.6		33.7	33.2	235.74	215.64	198.64	39.2	39.05	39.2	12.36	13.31	12.66	11.84		18		48.55		2.5		225.45		4.86		0.96		103		1.186		1.1		4.31		2.18	2.22
9.5	8.3	6.46	6.11	6.34	1.42	125.4	2.10	124.6		37.24	36.74	215.64	183.22	166.22	40.96	40.81	39.67	8.46	9.41	8.76	11.6		21		41.75		3.3		185.55		4.65		0.83		106		1.116		1.16		4.12		2.46	2.23
12.9	11.7	6.46	6.11	6.34	1.75	125.6	2.30	131.2		34.15	33.65	230.15	277.86	260.86	41.73	41.58	42.53	11.56	12.51	11.86	13.7		21		74.5		3.2		215.75		2.88		0.79		103		1.34		1.23		4.23		2.22	2.46
9.8	8.6	6.32	5.97	6.2	2.12	127.6	1.88	125.6		33.7	33.2	253.36	215.75	198.75	42.36	42.21	41.73	12.34	13.29	12.64	16.5		7		46		2.8		240.2		4.38		0.81		105		1.34		1.14		4.31		2.37	2.22
8.9	7.7	6.56	6.21	6.44	1.78	121.9	2.00	137.8		37.21	36.71	277.86	284.53	267.53	42.73	42.58	42.53	9.42	10.37	9.72	22		18		43.5		2.9		225.45		4.52		0.87		103		2.152		1.1		3.91		2.33	2.21
10.7	9.5	6.46	6.11	6.34	2.39	132.6	1.80	119.6		35.56	35.06	284.53	210.5	193.5	39.53	39.38	42.73	12.25	13.2	12.55	17.75		21		43.62		3.2		185.55		4.71		0.79		102		1.194		1.23		4.24		2.34	2.22
7.1	5.9	6.46	6.11	6.34	2.73	118.2	1.80	125.4		33.7	33.2	281.23	230.15	213.15	40.97	40.82	42.36	12.95	13.9	13.25	17.75		9		48.55		3.4		172.35		4.38		0.92		103		1.132		1.29		4.17		2.28	2.46
12.5	11.3	6.46	6.11	6.34	2.56	124.6	2.00	122.3		34.15	33.65	220.19	215.64	198.64	42.77	42.62	40.96	9.25	10.2	9.55	23.2		16		45.18		2.7		195.25		4.68		0.85		105		1.116		1.08		4.26		2.18	2.18
9.5	8.3	6.46	6.11	6.34	1.40	125.60	2.20	127.6		34.42	33.92	143.75	277.86	260.86	42.36	42.21	40.97	8.12	9.07	8.42	17.5		18		45.25		2.7		215.75		2.88		0.92		103		1.186		1.23		4.12		2.22	2.36
11.2	10	6.46	6.11	6.34	1.75	116.3	2.10	118.2		33.7	33.2	277.86	220.19	203.19	39.75	39.6	39.75	7.35	8.3	7.65	17		21		42.36		2.5		227.53		4.93		0.79		105		1.194		1.19		3.91		2.21	2.28
8.2	7	6.46	6.11	6.34	2.47	125.4	1.90	131.2		34.75	34.25	249.27	227.53	210.53	42.36	42.21	42.77	12.95	13.9	13.25	15.4		15		47.83		3.2		235.5		4.52		0.81		106		1.34		1.1		4.24		2.46	2.22
9.5	8.3	6.46	6.11	6.34	1.55	137.8	1.80	124.6		35.2	34.7	183.22	235.74	218.74	39.67	39.52	41.69	7.35	8.3	7.65	18		21		14.75		2.9		185.55		4.65		0.89		103		1.132		1.23		4.12		2.36	2.33
9.8	8.6	6.56	6.21	6.44	1.97	119.6	1.20	121.6		33.7	33.2	215.64	210.5	193.5	42.53	42.38	42.36	7.35	8.3	7.65	21.72		18		43.5		3.4		215.75		4.52		0.79		102		2.143		1.14		4.16		2.23	2.34
11.2	10	6.46	6.11	6.34	1.42	122.3	1.22	125.6		34.59	34.09	253.36	284.53	267.53	42.73	42.58	42.73	9.25	10.2	9.55	22		18		46		2.8		225.45		4.38		0.85		103		1.116		1.39		4.31		2.37	2.22
12.9	11.7	5.09	4.74	4.97	2.12	121.9	1.22	125.4		36.59	36.09	215.75	277.86	260.86	40.96	40.81	42.36	9.42	10.37	9.72	16.5		21		41.75		3.2		217.42		4.71		0.79		103		1.194		1.23		3.91		2.22	2.46
9.5	8.3	6.46	6.11	6.34	1.55	127.6	2.30	118.2		34.42	33.92	277.86	183.22	166.22	40.97	40.82	42.53	11.56	12.51	11.86	12.83		21		45.18		2.6		172.35		4.63		0.92		103		1.168		1.36		4.24		2.18	2.46
9.8	8.6	6.46	6.11	6.34	1.75	125.6	2.20	132.6		35.56	35.06	235.74	215.75	198.75	42.53	42.38	39.75	8.46	9.41	8.76	11.6		18		46.42		3.4		195.25		2.88		0.83		105		2.152		1.14		4.09		2.08	2.18
7.1	5.9	6.46	6.11	6.34	2.57	118.2	2.10	137.8		34.15	33.65	220.19	215.64	198.64	41.69	41.54	40.97	8.12	9.07	8.42	17.75		16		47.83		3.3		172.35		4.86		0.81		106		1.186		1.23		4.12		2.34	2.22
9.5	8.3	4.42	4.07	4.3	1.69	116.3	1.80	127.6		37.25	36.75	249.27	220.19	203.19	39.53	39.38	40.96	11.55	12.5	11.85	18		16		48.55		3.2		215.75		4.71		0.87		103		1.34		1.16		4.35		2.46	2.08
10.7	9.5	4.42	4.07	4.3	1.78	125.4	1.80	131.2		37.21	36.71	230.15	210.5	193.5	41.73	41.58	42.36	12.95	13.9	13.25	19.4		18		46.25		2.5		185.55		2.88		0.79		102		1.116		1.1		3.91		2.21	2.37
8.2	7	4.42	4.07	4.3	1.89	119.6	1.70	124.6		36.39	35.89	253.36	277.86	260.86	42.53	42.38	39.53	11.68	12.63	11.98	13.7		18		43.5		2.7		215.75		4.38		0.94		102		1.194		1.23		4.23		2.22	2.36
9.8	8.6	5.09	4.74	4.97	1.55	124.6	2.40	116.3		34.42	33.92	277.86	281.23	264.23	39.75	39.6	39.75	9.42	10.37	9.72	17.75		21		45.25		2.8		225.45		4.65		0.85		103		1.132		1.08		4.31		2.36	2.33
7.7	6.5	4.42	4.07	4.3	1.75	124.8	2.10	121.6		34.15	33.65	230.15	227.53	210.53	42.73	42.58	40.97	7.35	8.3	7.65	22		9		46		3.4		215.75		4.63		0.79		103		1.168		1.14		4.12		2.18	2.22
12.9	11.7	4.42	4.07	4.3	1.58	121.9	1.90	125.6		34.59	34.09	227.53	143.75	126.75	42.77	42.62	42.73	12.95	13.9	13.25	17		21		45.18		3.2		185.95		4.61		0.92		103		2.143		1.23		4.19		2.28	2.21
9.8	8.6	4.42	4.07	4.3	1.75	118.2	2.10	122.3		37.21	36.71	143.75	230.15	213.15	42.53	42.38	39.2	8.12	9.07	8.42	16.5		21		46.42		2.5		227.53		4.52		0.81		103		1.132		1.29		3.91		2.33	2.22
9.5	8.3	8.09	7.74	7.97	2.54	121.6	2.00	119.6		37.22	36.72	215.64	277.86	260.86	40.97	40.82	39.2	11.55	12.5	11.85	12.83		18		74.5		3.6		215.75		4.38		0.89		105		1.116		1.1		4.09		2.28	2.28
11.2	10	8.09	7.74	7.97	2.12	119.6	2.00	121.9		37.21	36.71	277.86	210.5	193.5	39.53	39.38	40.96	7.35	8.3	7.65	23.2		24		41.75		2.7		230.75		2.88		0.92		105		1.34		1.23		4.12		2.22	2.18
12.5	11.3	5.09	4.74	4.97	1.42	125.4	2.20	127.6		33.7	33.2	253.36	235.74	218.74	40.96	40.81	42.36	11.56	12.51	11.86	13.4		9		42.36		3.2		172.35		4.38		0.79		103		1.194		1.16		4.24		2.46	2.46
7.1	5.9	8.09	7.74	7.97	1.89	116.3	2.30	119.6		36.59	36.09	220.19	215.64	198.64	42.53	42.38	39.75	9.25	10.2	9.55	17.75		18		14.75		3.4		185.55		4.86		0.79		103		2.152		1.1		4.31		2.37	2.34
8.9	7.7	8.09	7.74	7.97	2.39	127.6	1.70	131.2		35.56	35.06	183.22	220.19	203.19	39.2	39.05	41.73	8.46	9.41	8.76	23.2		18		43.5		2.5		195.25		4.98		0.87		105		1.168		1.23		3.91		2.18	2.37
10.6	9.4	6.32	5.97	6.2	1.55	125.6	1.80	118.2		34.59	34.09	215.75	183.22	166.22	42.77	42.62	42.73	9.42	10.37	9.72	22		21		48.55		2.9		215.75		2.88		0.83		103		1.186		1.29		4.23		2.34	2.36
11.2	10	8.09	7.74	7.97	2.47	118.2	2.00	124.6		37.21	36.71	281.23	215.75	198.75	42.73	42.58	39.67	8.12	9.07	8.42	11.6		16		45.18		3.3		215.75		4.65		0.81		102		1.116		1.14		4.12		2.36	2.22
9.5	8.3	8.09	7.74	7.97	2.56	121.9	2.20	125.6		34.15	33.65	277.86	227.53	210.53	42.53	42.38	42.77	7.35	8.3	7.65	17.5		21		46		2.7		225.45		4.52		0.85		103		1.194		1.23		4.16		2.22	2.33
9.8	8.6	8.09	7.74	7.97	2.35	124.6	1.80	132.6		37.49	36.99	227.53	210.5	193.5	39.67	39.52	42.36	12.25	13.2	12.55	16.5		10		45.25		3.2		215.75		4.63		0.89		103		1.34		1.1		4.12		2.18	2.18
12.5	11.3	8.09	7.74	7.97	1.75	132.6	1.90	121.9		34.75	34.25	253.36	249.27	232.27	40.97	40.82	40.96	11.46	12.41	11.76	15.4		15		43.25		2.7		172.35		2.88		0.85		106		1.132		1.16		3.91		2.23	2.21
9.5	8.3	8.12	7.77	8	2.57	125.4	2.00	118.2		35.2	34.7	235.74	281.23	264.23	42.36	42.21	40.97	7.35	8.3	7.65	17		18		43.62		2.9		215.75		4.38		0.79		103		2.143		1.23		4.31		2.22	2.23
10.7	9.5	8.09	7.74	7.97	1.69	122.3	2.20	137.8		36.39	35.89	143.75	215.64	198.64	42.53	42.38	39.53	12.95	13.9	13.25	13.7		21		47.35		3.4		185.55		4.93		0.92		103		1.34		1.18		4.35		2.08	2.22
7.7	6.5	8.09	7.74	7.97	1.89	121.9	2.30	137.8		34.15	33.65	220.19	230.15	213.15	41.73	41.58	39.75	12.25	13.2	12.55	17.75		18		41.75		3.2		215.75		4.53		0.85		102		1.116		1.29		3.91		2.28	2.37
11.2	10	8.09	7.74	7.97	2.12	116.3	2.30	125.6		37.21	36.71	277.86	183.22	166.22	39.75	39.6	42.73	8.12	9.07	8.42	22		18		43.5		3.6		215.75		4.92		0.81		105		2.152		1.23		4.31		2.46	2.28
9.5	8.3	8.09	7.74	7.97	1.78	125.6	2.90	124.6		33.7	33.2	215.75	235.74	218.74	41.69	41.54	42.36	7.35	8.3	7.65	17.75		16		45.18		3.3		172.35		4.38		0.83		103		1.168		1.1		4.12		2.37	2.18
9.5	8.3	8.09	7.74	7.97	1.55	137.8	1.38	127.6		34.42	33.92	215.64	253.36	236.36	42.53	42.38	42.53	9.42	10.37	9.72	21.72		21		48.55		3.4		224.65		4.86		0.87		105		1.194		1.08		4.24		2.18	2.21
8.2	7	8.09	7.74	7.97	1.42	127.6	1.28	121.9		37.49	36.99	253.36	227.53	210.53	40.96	40.81	42.36	11.56	12.51	11.86	11.84		10		46		2.7		215.75		4.71		0.96		102		1.34		1.23		4.17		2.22	2.36
11.2	10	6.32	5.97	6.2	2.63	118.2	1.18	122.3		34.59	34.09	281.23	215.75	198.75	42.53	42.38	41.73	7.35	8.3	7.65	16.5		18		42.36		2.7		185.55		4.65		0.89		102		2.143		1.39		3.91		2.21	2.22
7.1	5.9	8.09	7.74	7.97	2.21	125.4	1.25	124.6		37.24	36.74	227.53	277.86	260.86	42.73	42.58	42.53	12.95	13.9	13.25	18		16		47.83		2.8		230.75		4.38		0.79		103		1.116		1.1		4.12		2.34	2.23
9.8	8.6	7.09	6.74	6.97	1.89	124.6	1.25	116.3		37.25	36.75	277.86	220.19	203.19	42.36	42.21	42.77	11.55	12.5	11.85	11.84		14		45.25		3.2		225.45		2.88		0.79		106		1.186		1.23		4.24		2.36	2.46
9.5	8.3	8.09	7.74	7.97	1.97	124.8	1.25	121.6		34.42	33.92	183.22	284.53	267.53	39.53	39.38	40.96	7.35	8.3	7.65	17.75		10		43.62		3.4		235.5		4.61		0.81		103		2.136		1.16		4.16		2.28	2.22
12.1	10.9	5.09	4.74	4.97	2.47	121.9	1.22	118.2		36.59	36.09	230.15	215.64	198.64	40.97	40.82	41.69	7.35	8.3	7.65	11.6		18		47.83		2.5		215.75		4.53		0.92		103		1.34		1.14		4.31		2.46	2.18
8.3	7.1	8.09	7.74	7.97	2.54	125.6	1.26	119.6		35.56	35.06	220.19	253.36	236.36	42.53	42.38	42.36	8.46	9.41	8.76	22		18		45.18		3.6		185.55		4.52		0.83		105		2.152		1.23		3.91		2.33	2.22
9.5	8.3	8.09	7.74	7.97	1.55	122.3	2.47	125.6		37.24	36.74	215.75	143.75	126.75	42.77	42.62	42.73	11.55	12.5	11.85	17		14		43.5		2.9		215.75		4.38		0.92		102		1.194		1.1		4.12		2.22	2.21
8.3	7.1	8.09	7.74	7.97	2.12	118.2	2.56	124.6		34.59	34.09	277.86	277.86	260.86	39.67	39.52	42.36	9.42	10.37	9.72	17.5		9		46.25		3.3		195.25		4.63		0.85		102		1.116		1.18		4.09		2.18	2.18
12.5	11.3	8.09	7.74	7.97	2.56	127.6	2.35	118.2		34.42	33.92	281.23	281.23	264.23	39.53	39.38	42.53	7.35	8.3	7.65	17.75		10		46		3.4		172.35		2.88		0.87		103		1.34		1.29		4.19		2.08	2.36
12.9	11.7	8.09	7.74	7.97	1.42	125.4	1.25	132.6		34.15	33.65	235.74	183.22	166.22	42.36	42.21	39.75	11.55	12.5	11.85	19.4		17		41.75		2.9		215.75		4.86		0.79		107		1.168		1.1		4.12		2.28	2.46
11.2	10	8.09	7.74	7.97	2.35	119.6	2.57	121.9		36.39	35.89	227.53	227.53	210.53	42.77	42.62	40.97	9.25	10.2	9.55	16.5		7		74.5		3.2		215.75		4.38		0.81		102		2.143		1.23		3.91		2.37	2.28
8.2	7	8.09	7.74	7.97	2.57	132.6	2.56	127.6		36.59	36.09	215.64	253.36	236.36	42.73	42.58	40.96	11.56	12.51	11.86	13.4		18		47.83		3.6		185.55		4.52		0.83		103		1.186		1.23		4.16		2.36	2.22
9.8	8.6	5.09	4.74	4.97	2.21	124.6	1.97	122.3		33.7	33.2	277.86	220.19	203.19	39.75	39.6	42.36	7.35	8.3	7.65	13.7		16		48.55		2.9		230.75		4.65		0.89		103		1.194		1.14		4.12		2.22	2.18
8.9	7.7	8.09	7.74	7.97	1.40	131.20	2.43	125.6		37.24	36.74	253.36	277.86	260.86	40.97	40.82	39.53	8.12	9.07	8.42	22		15		43.25		3.4		215.75		4.93		0.92		105		1.116		1.16		4.24		2.46	2.36
8.3	7.1	8.09	7.74	7.97	1.55	125.6	1.89	127.6		35.56	35.06	143.75	215.75	198.75	42.53	42.38	42.73	11.68	12.63	11.98	17.5		14		14.75		2.7		185.55		4.92		0.83		106		1.168		1.1		4.23		2.18	2.46
10.7	9.5	8.09	7.74	7.97	1.78	137.8	2.12	121.9		35.2	34.7	220.19	230.15	213.15	42.36	42.21	39.75	9.42	10.37	9.72	15.4		18		43.5		3.2		225.45		2.88		0.79		103		1.34		1.23		3.91		2.21	2.33
8.2	7	8.09	7.74	7.97	2.39	119.6	1.42	122.3		34.75	34.25	183.22	215.64	198.64	42.53	42.38	41.69	7.35	8.3	7.65	19.4		18		46		2.8		172.35		4.71		0.85		102		1.132		1.24		4.17		2.34	2.18
7.7	6.5	8.12	7.77	8	2.47	125.4	1.75	132.6		34.42	33.92	215.75	253.36	236.36	41.73	41.58	42.77	12.25	13.2	12.55	17		20		42.38		3.3		225.45		4.31		0.79		103		1.194		1.23		4.12		2.37	2.34
12.1	10.9	8.09	7.74	7.97	2.73	122.3	1.69	125.6		37.49	36.99	277.86	235.74	218.74	40.96	40.81	42.36	12.95	13.9	13.25	17.75		10		45.25		2.9		172.35		4.52		0.81		106		2.152		1.39		4.35		2.22	2.46
9.8	8.6	5.09	4.74	4.97	1.69	127.6	1.76	121.9		37.24	36.74	253.36	277.86	260.86	42.53	42.38	41.73	8.46	9.41	8.76	13.7		24		47.83		2.5		215.75		4.63		0.92		105		1.116		1.1		4.23		2.28	2.37
9.5	8.3	8.09	7.74	7.97	1.75	118.2	2.35	119.6		35.56	35.06	253.36	249.27	232.27	40.97	40.82	42.73	12.34	13.29	12.64	15.4		9		42.36		3.4		185.95		4.38		0.85		105		1.186		1.23		3.91		2.33	2.22
8.9	7.7	4.42	4.07	4.3	2.54	131.2	2.43	118.2		35.2	34.7	183.22	230.15	213.15	42.73	42.58	40.96	7.35	8.3	7.65	11.6		19		46.25		3.2		185.55		2.88		0.79		103		1.34		1.16		4.16		2.34	2.18
11.2	10	7.45	7.1	7.33	2.56	124.6	2.12	125.6		33.7	33.2	235.74	220.19	203.19	39.75	39.6	42.53	12.95	13.9	13.25	22		16		48.55		2.7		172.35		4.92		0.92		103		1.194		1.19		4.35		2.18	2.18
12.9	11.7	6.32	5.97	6.2	2.21	121.6	1.22	124.6		34.75	34.25	215.64	227.53	210.53	42.53	42.38	39.67	12.95	13.9	13.25	17.75		21		45.18		3.4		215.75		4.38		0.87		102		1.168		1.23		4.24		2.46	2.22
7.1	5.9	4.42	4.07	4.3	2.35	125.6	1.26	116.3		34.59	34.09	220.19	281.23	264.23	40.97	40.82	42.36	11.56	12.51	11.86	17		13		46		3.2		215.75		4.93		0.92		105		2.152		1.1		3.91		2.22	2.36
8.3	7.1	4.42	4.07	4.3	2.39		2.12	119.6		33.7	33.2	227.53	210.5	193.5	42.73	42.58	42.77	7.35	8.3	7.65	17.75		13		46		2.9		215.75		4.61		0.79		103		1.116		1.23		4.12		2.23	2.46
9.8	8.6	5.09	4.74	4.97	1.58		2.56	127.6		34.42	33.92	277.86	227.53	210.53	42.36	42.21	39.75	9.42	10.37	9.72	16.5		20		48.55		3.4		215.75		2.88		0.83		103		1.194		1.34		4.31		2.22	2.33
8.9	7.7	4.42	4.07	4.3	1.97		2.57	125.6		34.15	33.65	253.36	277.86	260.86	42.53	42.38	39.2	7.35	8.3	7.65	18		15		45.18		2.9		185.55		4.92		0.81		103		1.34		1.23		3.91		2.18	2.22
	8.700344828		5.982034483	6.212034483			2.01				34.57151724			214.349138	41.54	41.39	41.57117241	9.703137931	10.65313793	10.00313793	17.202						3.026896552												1.183517241					
												231.349138				41.39																												

**Table 12:** Data file. Lipid index and metabolic index. TC: total cholesterol; TG: triglycerides; HDL-C: high-density lipoprotein cholesterol; LDL-C: low-density lipoprotein cholesterol; WHtR: waist-to-height ratio

SN	TC (mmol/L)		TG (mmol/L)	1.56			HDL-C (mmol/L)		LDL-C (mmol/L)		WHtr		Cardiometabolic index	Control group		TC (mmol/L)		TG (mmol/L)			HDL-C (mmol/L)		LDL-C (mmol/L)		WHtr		Cardiometabolic index
1	4.96	5.16	1.92	2.06		1.42	2.01	2.31		0.54		0.13			4.96	4.99	1.92	1.8		1.42	1.56	2.31	2.597	0.54	0.503	0.13	0.108
2	4.12	4.32	1.74	1.88		1.61	2.2	1.96		0.51		0.13			4.12	4.15	1.74	1.62		1.61	1.75	1.96	2.247	0.51	0.473	0.13	0.108
3	3.91	4.11	0.79	0.93		1.71	2.3	2.69		0.49		0.9			3.91	3.94	0.79	0.67		1.71	1.85	2.69	2.977	0.49	0.453	0.9	0.878
4	4.51	4.71	1.92	2.06		1.43	2.02	1.72		0.51		0.9			4.51	4.54	1.92	1.8		1.43	1.57	1.72	2.007	0.51	0.473	0.9	0.878
5	4.82	5.02	1.92	2.06		1.61	2.2	1.96		0.45		0.13			4.82	4.85	1.92	1.8		1.61	1.75	1.96	2.247	0.45	0.413	0.13	0.108
6	4.51	4.71	0.79	0.93		1.71	2.3	2.31		0.49		0.13			4.51	4.54	0.79	0.67		1.71	1.85	2.31	2.597	0.49	0.453	0.13	0.108
7	4.82	5.02	1.92	2.06		1.76	2.35	2.61		0.51		0.13			4.82	4.85	1.92	1.8		1.76	1.9	2.61	2.897	0.51	0.473	0.13	0.108
8	4.51	4.71	0.91	1.05		1.42	2.01	1.96		0.54		0.9			4.51	4.54	0.91	0.79		1.42	1.56	1.96	2.247	0.54	0.503	0.9	0.878
9	4.96	5.16	0.79	0.93		1.61	2.2	1.72		0.45		0.13			4.96	4.99	0.79	0.67		1.61	1.75	1.72	2.007	0.45	0.413	0.13	0.108
10	4.51	4.71	1.62	1.76		1.42	2.01	2.64		0.49		0.13			4.51	4.54	1.62	1.5		1.42	1.56	2.64	2.927	0.49	0.453	0.13	0.108
11	3.91	4.11	0.91	1.05		1.71	2.3	1.96		0.51		0.13			3.91	3.94	0.91	0.79		1.71	1.85	1.96	2.247	0.51	0.473	0.13	0.108
12	4.51	4.71	1.92	2.06		1.42	2.01	2.31		0.53		0.9			4.51	4.54	1.92	1.8		1.42	1.56	2.31	2.597	0.53	0.493	0.9	0.878
13	4.68	4.88	1.74	1.88		1.43	2.02	1.72		0.53		0.13			4.68	4.71	1.74	1.62		1.43	1.57	1.72	2.007	0.53	0.493	0.13	0.108
14	4.51	4.71	0.89	1.03		1.78	2.37	1.96		0.51		0.13			4.51	4.54	0.89	0.77		1.78	1.92	1.96	2.247	0.51	0.473	0.13	0.108
15	4.82	5.02	1.74	1.88		1.71	2.3	2.69		0.49		0.13			4.82	4.85	1.74	1.62		1.71	1.85	2.69	2.977	0.49	0.453	0.13	0.108
16	4.51	4.71	1.36	1.5		1.76	2.35	2.64		0.54		0.13			4.51	4.54	1.36	1.24		1.76	1.9	2.64	2.927	0.54	0.503	0.13	0.108
17	3.91	4.11	1.74	1.88		1.71	2.3	1.96		0.53		0.9			3.91	3.94	1.74	1.62		1.71	1.85	1.96	2.247	0.53	0.493	0.9	0.878
18	4.51	4.71	1.62	1.76		1.61	2.2	1.72		0.51		0.13			4.51	4.54	1.62	1.5		1.61	1.75	1.72	2.007	0.51	0.473	0.13	0.108
19	4.96	5.16	1.74	1.88		1.43	2.02	2.61		0.49		0.13			4.96	4.99	1.74	1.62		1.43	1.57	2.61	2.897	0.49	0.453	0.13	0.108
20	4.51	4.71	1.92	2.06		1.76	2.35	1.96		0.45		0.9			4.51	4.54	1.92	1.8		1.76	1.9	1.96	2.247	0.45	0.413	0.9	0.878
21	4.68	4.88	1.51	1.65		1.43	2.02	2.31		0.51		0.13			4.68	4.71	1.51	1.39		1.43	1.57	2.31	2.597	0.51	0.473	0.13	0.108
22	3.91	4.11	0.79	0.93		1.71	2.3	1.72		0.54		0.13			3.91	3.94	0.79	0.67		1.71	1.85	1.72	2.007	0.54	0.503	0.13	0.108
23	4.82	5.02	1.51	1.65		1.61	2.2	1.96		0.49		0.9			4.82	4.85	1.51	1.39		1.61	1.75	1.96	2.247	0.49	0.453	0.9	0.878
24	4.51	4.71	0.91	1.05		1.42	2.01	2.69		0.51		0.13			4.51	4.54	0.91	0.79		1.42	1.56	2.69	2.977	0.51	0.473	0.13	0.108
25	4.96	5.16	0.89	1.03		1.92	2.51	2.43		0.44		0.13			4.96	4.99	0.89	0.77		1.92	2.06	2.43	2.717	0.44	0.403	0.13	0.108
26	3.91	4.11	1.51	1.65		1.42	2.01	1.72		0.52		0.9			3.91	3.94	1.51	1.39		1.42	1.56	1.72	2.007	0.52	0.483	0.9	0.878
27	4.68	4.88	1.92	2.06		1.78	2.37	2.31		0.49		0.13			4.68	4.71	1.92	1.8		1.78	1.92	2.31	2.597	0.49	0.453	0.13	0.108
28	4.51	4.71	1.13	1.27		1.78	2.37	2.64		0.51		0.13			4.51	4.54	1.13	1.01		1.78	1.92	2.64	2.927	0.51	0.473	0.13	0.108
29	4.82	5.02	0.89	1.03		1.71	2.3	1.72		0.54		0.9			4.82	4.85	0.89	0.77		1.71	1.85	1.72	2.007	0.54	0.503	0.9	0.878
30	3.91	4.11	1.51	1.65		1.61	2.2	2.31		0.45		0.13			3.91	3.94	1.51	1.39		1.61	1.75	2.31	2.597	0.45	0.413	0.13	0.108
31	3.88	4.08	1.92	2.06		1.47	2.06	2.69		0.49		0.13			3.88	3.91	1.92	1.8		1.47	1.61	2.69	2.977	0.49	0.453	0.13	0.108
32	4.51	4.71	0.91	1.05		1.78	2.37	1.96		0.53		0.9			4.51	4.54	0.91	0.79		1.78	1.92	1.96	2.247	0.53	0.493	0.9	0.878
33	4.36	4.56	1.92	2.06		1.76	2.35	1.72		0.54		0.13			4.36	4.39	1.92	1.8		1.76	1.9	1.72	2.007	0.54	0.503	0.13	0.108
34	4.82	5.02	1.74	1.88		1.61	2.2	2.61		0.51		0.13			4.82	4.85	1.74	1.62		1.61	1.75	2.61	2.897	0.51	0.473	0.13	0.108
35	3.91	4.11	0.79	0.93		1.71	2.3	1.96		0.44		0.9			3.91	3.94	0.79	0.67		1.71	1.85	1.96	2.247	0.44	0.403	0.9	0.878
36	4.51	4.71	1.74	1.88		1.92	2.51	1.72		0.49		0.13			4.51	4.54	1.74	1.62		1.92	2.06	1.72	2.007	0.49	0.453	0.13	0.108
37	4.36	4.56	1.62	1.76		1.42	2.01	2.64		0.51		0.13			4.36	4.39	1.62	1.5		1.42	1.56	2.64	2.927	0.51	0.473	0.13	0.108
38	4.68	4.88	1.92	2.06		1.42	2.01	1.96		0.45		0.9			4.68	4.71	1.92	1.8		1.42	1.56	1.96	2.247	0.45	0.413	0.9	0.878
39	4.82	5.02	1.36	1.5		1.78	2.37	1.72		0.47		0.13			4.82	4.85	1.36	1.24		1.78	1.92	1.72	2.007	0.47	0.433	0.13	0.108
40	4.51	4.71	0.79	0.93		1.61	2.2	2.31		0.51		0.13			4.51	4.54	0.79	0.67		1.61	1.75	2.31	2.597	0.51	0.473	0.13	0.108
41	3.91	4.11	0.91	1.05		1.76	2.35	1.96		0.49		0.9			3.91	3.94	0.91	0.79		1.76	1.9	1.96	2.247	0.49	0.453	0.9	0.878
42	4.96	5.16	0.99	1.13		1.71	2.3	2.73		0.54		0.13			4.96	4.99	0.99	0.87		1.71	1.85	2.73	3.017	0.54	0.503	0.13	0.108
43	4.96	5.16	0.89	1.03		1.78	2.37	1.72		0.53		0.9			4.96	4.99	0.89	0.77		1.78	1.92	1.72	2.007	0.53	0.493	0.9	0.878
44	4.51	4.71	1.92	2.06		1.43	2.02	1.96		0.51		0.13			4.51	4.54	1.92	1.8		1.43	1.57	1.96	2.247	0.51	0.473	0.13	0.108
45	3.79	3.99	1.62	1.76		1.61	2.2	2.69		0.49		0.9			3.79	3.82	1.62	1.5		1.61	1.75	2.69	2.977	0.49	0.453	0.9	0.878
46	4.96	5.16	0.91	1.05		1.78	2.37	1.72		0.45		0.13			4.96	4.99	0.91	0.79		1.78	1.92	1.72	2.007	0.45	0.413	0.13	0.108
47	3.91	4.11	1.36	1.5		1.71	2.3	1.96		0.51		0.9			3.91	3.94	1.36	1.24		1.71	1.85	1.96	2.247	0.51	0.473	0.9	0.878
48	4.82	5.02	0.91	1.05		1.59	2.18	2.31		0.54		0.9			4.82	4.85	0.91	0.79		1.59	1.73	2.31	2.597	0.54	0.503	0.9	0.878
49	4.51	4.71	1.62	1.76		1.78	2.37	1.72		0.53		0.13			4.51	4.54	1.62	1.5		1.78	1.92	1.72	2.007	0.53	0.493	0.13	0.108
50	4.36	4.56	0.91	1.05		1.42	2.01	1.96		0.49		0.9			4.36	4.39	0.91	0.79		1.42	1.56	1.96	2.247	0.49	0.453	0.9	0.878
51	4.96	5.16	1.74	1.88		1.42	2.01	2.61		0.51		0.9			4.96	4.99	1.74	1.62		1.42	1.56	2.61	2.897	0.51	0.473	0.9	0.878
52	4.96	5.16	1.51	1.65		1.61	2.2	1.72		0.44		0.13			4.96	4.99	1.51	1.39		1.61	1.75	1.72	2.007	0.44	0.403	0.13	0.108
53	4.68	4.88	1.74	1.88		1.43	2.02	2.69		0.45		0.9			4.68	4.71	1.74	1.62		1.43	1.57	2.69	2.977	0.45	0.413	0.9	0.878
54	4.51	4.71	0.79	0.93		1.42	2.01	1.72		0.51		0.9			4.51	4.54	0.79	0.67		1.42	1.56	1.72	2.007	0.51	0.473	0.9	0.878
55	4.82	5.02	1.74	1.88		1.71	2.3	2.31		0.54		0.13			4.82	4.85	1.74	1.62		1.71	1.85	2.31	2.597	0.54	0.503	0.13	0.108
56	4.17	4.37	0.89	1.03		1.78	2.37	1.72		0.47		0.9			4.17	4.2	0.89	0.77		1.78	1.92	1.72	2.007	0.47	0.433	0.9	0.878
57	4.96	5.16	1.74	1.88		1.42	2.01	2.43		0.53		0.9			4.96	4.99	1.74	1.62		1.42	1.56	2.43	2.717	0.53	0.493	0.9	0.878
58	3.91	4.11	1.13	1.27		1.66	2.25	2.69		0.51		0.13			3.91	3.94	1.13	1.01		1.66	1.8	2.69	2.977	0.51	0.473	0.13	0.108
59	4.51	4.71	1.74	1.88		1.61	2.2	1.96		0.49		0.9			4.51	4.54	1.74	1.62		1.61	1.75	1.96	2.247	0.49	0.453	0.9	0.878
60	3.88	4.08	0.89	1.03		1.42	2.01	2.64		0.54		0.9			3.88	3.91	0.89	0.77		1.42	1.56	2.64	2.927	0.54	0.503	0.9	0.878
61	4.36	4.56	1.74	1.88		1.43	2.02	2.31		0.51		0.13			4.36	4.39	1.74	1.62		1.43	1.57	2.31	2.597	0.51	0.473	0.13	0.108
62	4.96	5.16	0.91	1.05		1.42	2.01	1.96		0.45		0.9			4.96	4.99	0.91	0.79		1.42	1.56	1.96	2.247	0.45	0.413	0.9	0.878
63	4.96	5.16	1.74	1.88		1.71	2.3	2.76		0.49		0.9			4.96	4.99	1.74	1.62		1.71	1.85	2.76	3.047	0.49	0.453	0.9	0.878
64	3.91	4.11	1.92	2.06		1.78	2.37	2.31		0.51		0.13			3.91	3.94	1.92	1.8		1.78	1.92	2.31	2.597	0.51	0.473	0.13	0.108
65	4.68	4.88	1.74	1.88		1.76	2.35	1.96		0.52		0.9			4.68	4.71	1.74	1.62		1.76	1.9	1.96	2.247	0.52	0.483	0.9	0.878
66	4.51	4.71	1.51	1.65		1.43	2.02	2.69		0.54		0.9			4.51	4.54	1.51	1.39		1.43	1.57	2.69	2.977	0.54	0.503	0.9	0.878
67	3.96	4.16	1.74	1.88		1.61	2.2	2.31		0.49		0.9			3.96	3.99	1.74	1.62		1.61	1.75	2.31	2.597	0.49	0.453	0.9	0.878
68	4.36	4.56	1.62	1.76		1.71	2.3	1.96		0.51		0.9			4.36	4.39	1.62	1.5		1.71	1.85	1.96	2.247	0.51	0.473	0.9	0.878
69	4.51	4.71	1.74	1.88		1.78	2.37	1.72		0.44		0.13			4.51	4.54	1.74	1.62		1.78	1.92	1.72	2.007	0.44	0.403	0.13	0.108
70	3.79	3.99	1.36	1.5		1.92	2.51	2.69		0.45		0.13			3.79	3.82	1.36	1.24		1.92	2.06	2.69	2.977	0.45	0.413	0.13	0.108
71	3.91	4.11	1.92	2.06		1.43	2.02	1.96		0.51		0.13			3.91	3.94	1.92	1.8		1.43	1.57	1.96	2.247	0.51	0.473	0.13	0.108
72	4.96	5.16	1.64	1.78		1.59	2.18	1.72		0.49		0.13			4.96	4.99	1.64	1.52		1.59	1.73	1.72	2.007	0.49	0.453	0.13	0.108
73	4.51	4.71	0.91	1.05		1.42	2.01	2.61		0.54		0.13			4.51	4.54	0.91	0.79		1.42	1.56	2.61	2.897	0.54	0.503	0.13	0.108
74	4.96	5.16	0.79	0.93		1.61	2.2	1.96		0.51		0.13			4.96	4.99	0.79	0.67		1.61	1.75	1.96	2.247	0.51	0.473	0.13	0.108
75	4.68	4.88	1.51	1.65		1.42	2.01	1.72		0.56		0.13			4.68	4.71	1.51	1.39		1.42	1.56	1.72	2.007	0.56	0.523	0.13	0.108
76	3.88	4.08	1.92	2.06		1.71	2.3	2.76		0.49		0.13			3.88	3.91	1.92	1.8		1.71	1.85	2.76	3.047	0.49	0.453	0.13	0.108
77	4.82	5.02	1.62	1.76		1.42	2.01	1.96		0.51		0.13			4.82	4.85	1.62	1.5		1.42	1.56	1.96	2.247	0.51	0.473	0.13	0.108
78	3.91	4.11	0.79	0.93		1.43	2.02	1.72		0.53		0.13			3.91	3.94	0.79	0.67		1.43	1.57	1.72	2.007	0.53	0.493	0.13	0.108
79	4.36	4.56	0.89	1.03		1.42	2.01	2.31		0.54		0.9			4.36	4.39	0.89	0.77		1.42	1.56	2.31	2.597	0.54	0.503	0.9	0.878
80	3.79	3.99	0.99	1.13		1.47	2.06	1.96		0.45		0.13			3.79	3.82	0.99	0.87		1.47	1.61	1.96	2.247	0.45	0.413	0.13	0.108
81	4.96	5.16	1.74	1.88		1.42	2.01	1.72		0.51		0.13			4.96	4.99	1.74	1.62		1.42	1.56	1.72	2.007	0.51	0.473	0.13	0.108
82	4.96	5.16	1.92	2.06		1.92	2.51	2.69		0.49		0.13			4.96	4.99	1.92	1.8		1.92	2.06	2.69	2.977	0.49	0.453	0.13	0.108
83	3.91	4.11	1.74	1.88		1.42	2.01	1.96		0.54		0.13			3.91	3.94	1.74	1.62		1.42	1.56	1.96	2.247	0.54	0.503	0.13	0.108
84	4.51	4.71	0.79	0.93		1.47	2.06	1.72		0.44		0.13			4.51	4.54	0.79	0.67		1.47	1.61	1.72	2.007	0.44	0.403	0.13	0.108
85	3.96	4.16	1.74	1.88		1.42	2.01	2.31		0.51		0.13			3.96	3.99	1.74	1.62		1.42	1.56	2.31	2.597	0.51	0.473	0.13	0.108
86	4.68	4.88	0.91	1.05		1.71	2.3	1.96		0.49		0.13			4.68	4.71	0.91	0.79		1.71	1.85	1.96	2.247	0.49	0.453	0.13	0.108
87	4.51	4.71	1.74	1.88		1.61	2.2	1.72		0.47		0.13			4.51	4.54	1.74	1.62		1.61	1.75	1.72	2.007	0.47	0.433	0.13	0.108
88	3.88	4.08	1.51	1.65		1.76	2.35	2.64		0.51		0.9			3.88	3.91	1.51	1.39		1.76	1.9	2.64	2.927	0.51	0.473	0.9	0.878
89	3.79	3.99	1.74	1.88		1.71	2.3	1.96		0.54		0.9			3.79	3.82	1.74	1.62		1.71	1.85	1.96	2.247	0.54	0.503	0.9	0.878
90	4.51	4.71	1.62	1.76		1.71	2.3	1.72		0.49		0.13			4.51	4.54	1.62	1.5		1.71	1.85	1.72	2.007	0.49	0.453	0.13	0.108
91	3.91	4.11	1.74	1.88		1.78	2.37	2.69		0.51		0.13			3.91	3.94	1.74	1.62		1.78	1.92	2.69	2.977	0.51	0.473	0.13	0.108
92	4.36	4.56	1.92	2.06		1.71	2.3	1.96		0.45		0.13			4.36	4.39	1.92	1.8		1.71	1.85	1.96	2.247	0.45	0.413	0.13	0.108
93	4.51	4.71	1.74	1.88		1.59	2.18	2.31		0.53		0.13			4.51	4.54	1.74	1.62		1.59	1.73	2.31	2.597	0.53	0.493	0.13	0.108
94	4.96	5.16	0.79	0.93		1.71	2.3	2.61		0.51		0.9			4.96	4.99	0.79	0.67		1.71	1.85	2.61	2.897	0.51	0.473	0.9	0.878
95	4.96	5.16	1.74	1.88		1.92	2.51	1.96		0.54		0.13			4.96	4.99	1.74	1.62		1.92	2.06	1.96	2.247	0.54	0.503	0.13	0.108
96	4.51	4.71	1.36	1.5		1.61	2.2	1.72		0.49		0.9			4.51	4.54	1.36	1.24		1.61	1.75	1.72	2.007	0.49	0.453	0.9	0.878
97	3.79	3.99	1.64	1.78		1.42	2.01	2.43		0.52		0.13			3.79	3.82	1.64	1.52		1.42	1.56	2.43	2.717	0.52	0.483	0.13	0.108
98	4.68	4.88	1.74	1.88		1.76	2.35	1.96		0.51		0.13			4.68	4.71	1.74	1.62		1.76	1.9	1.96	2.247	0.51	0.473	0.13	0.108
99	4.51	4.71	0.91	1.05		1.42	2.01	2.69		0.45		0.9			4.51	4.54	0.91	0.79		1.42	1.56	2.69	2.977	0.45	0.413	0.9	0.878
100	4.49	4.69	1.92	2.06		1.71	2.3	2.31		0.49		0.13			4.49	4.52	1.92	1.8		1.71	1.85	2.31	2.597	0.49	0.453	0.13	0.108
101	3.88	4.08	1.74	1.88		1.42	2.01	1.96		0.51		0.9			3.88	3.91	1.74	1.62		1.42	1.56	1.96	2.247	0.51	0.473	0.9	0.878
102	4.51	4.71	0.79	0.93		1.78	2.37	2.73		0.54		0.13			4.51	4.54	0.79	0.67		1.78	1.92	2.73	3.017	0.54	0.503	0.13	0.108
103	3.79	3.99	1.51	1.65		1.42	2.01	1.72		0.44		0.13			3.79	3.82	1.51	1.39		1.42	1.56	1.72	2.007	0.44	0.403	0.13	0.108
104	4.82	5.02	1.74	1.88		1.66	2.25	1.96		0.51		0.9			4.82	4.85	1.74	1.62		1.66	1.8	1.96	2.247	0.51	0.473	0.9	0.878
105	4.96	5.16	1.62	1.76		1.42	2.01	2.31		0.53		0.13			4.96	4.99	1.62	1.5		1.42	1.56	2.31	2.597	0.53	0.493	0.13	0.108
106	4.96	5.16	0.89	1.03		1.78	2.37	2.69		0.49		0.13			4.96	4.99	0.89	0.77		1.78	1.92	2.69	2.977	0.49	0.453	0.13	0.108
107	4.36	4.56	1.74	1.88		1.42	2.01	1.96		0.54		0.9			4.36	4.39	1.74	1.62		1.42	1.56	1.96	2.247	0.54	0.503	0.9	0.878
108	4.68	4.88	1.92	2.06		1.71	2.3	2.61		0.51		0.13			4.68	4.71	1.92	1.8		1.71	1.85	2.61	2.897	0.51	0.473	0.13	0.108
109	3.91	4.11	0.79	0.93		1.61	2.2	2.31		0.56		0.13			3.91	3.94	0.79	0.67		1.61	1.75	2.31	2.597	0.56	0.523	0.13	0.108
110	4.82	5.02	1.74	1.88		1.43	2.02	1.96		0.49		0.13			4.82	4.85	1.74	1.62		1.43	1.57	1.96	2.247	0.49	0.453	0.13	0.108
111	3.79	3.99	0.99	1.13		1.78	2.37	1.72		0.47		0.9			3.79	3.82	0.99	0.87		1.78	1.92	1.72	2.007	0.47	0.433	0.9	0.878
112	3.96	4.16	0.91	1.05		1.61	2.2	2.69		0.51		0.13			3.96	3.99	0.91	0.79		1.61	1.75	2.69	2.977	0.51	0.473	0.13	0.108
113	3.88	4.08	1.74	1.88		1.66	2.25	1.96		0.54		0.13			3.88	3.91	1.74	1.62		1.66	1.8	1.96	2.247	0.54	0.503	0.13	0.108
114	3.91	4.11	1.62	1.76		1.78	2.37	2.64		0.49		0.9			3.91	3.94	1.62	1.5		1.78	1.92	2.64	2.927	0.49	0.453	0.9	0.878
115	4.96	5.16	1.92	2.06		1.78	2.37	2.31		0.53		0.13			4.96	4.99	1.92	1.8		1.78	1.92	2.31	2.597	0.53	0.493	0.13	0.108
116	4.96	5.16	1.74	1.88		1.42	2.01	1.96		0.51		0.13			4.96	4.99	1.74	1.62		1.42	1.56	1.96	2.247	0.51	0.473	0.13	0.108
117	4.51	4.71	0.79	0.93		1.43	2.02	2.69		0.45		0.13			4.51	4.54	0.79	0.67		1.43	1.57	2.69	2.977	0.45	0.413	0.13	0.108
118	4.82	5.02	1.36	1.5		1.42	2.01	2.76		0.54		0.13			4.82	4.85	1.36	1.24		1.42	1.56	2.76	3.047	0.54	0.503	0.13	0.108
119	4.17	4.37	1.74	1.88		1.71	2.3	1.96		0.51		0.13			4.17	4.2	1.74	1.62		1.71	1.85	1.96	2.247	0.51	0.473	0.13	0.108
120	3.91	4.11	1.13	1.27		1.42	2.01	1.72		0.49		0.9			3.91	3.94	1.13	1.01		1.42	1.56	1.72	2.007	0.49	0.453	0.9	0.878
121	4.68	4.88	0.91	1.05		1.61	2.2	2.31		0.44		0.9			4.68	4.71	0.91	0.79		1.61	1.75	2.31	2.597	0.44	0.403	0.9	0.878
122	4.36	4.56	1.74	1.88		1.42	2.01	1.96		0.56		0.13			4.36	4.39	1.74	1.62		1.42	1.56	1.96	2.247	0.56	0.523	0.13	0.108
123	3.88	4.08	1.92	2.06		1.78	2.37	2.43		0.51		0.13			3.88	3.91	1.92	1.8		1.78	1.92	2.43	2.717	0.51	0.473	0.13	0.108
124	4.82	5.02	1.62	1.76		1.42	2.01	2.69		0.49		0.9			4.82	4.85	1.62	1.5		1.42	1.56	2.69	2.977	0.49	0.453	0.9	0.878
125	3.91	4.11	1.74	1.88		1.76	2.35	1.96		0.54		0.13			3.91	3.94	1.74	1.62		1.76	1.9	1.96	2.247	0.54	0.503	0.13	0.108
126	3.96	4.16	0.79	0.93		1.42	2.01	2.31		0.45		0.13			3.96	3.99	0.79	0.67		1.42	1.56	2.31	2.597	0.45	0.413	0.13	0.108
127	4.96	5.16	0.89	1.03		1.71	2.3	2.61		0.51		0.9			4.96	4.99	0.89	0.77		1.71	1.85	2.61	2.897	0.51	0.473	0.9	0.878
128	3.79	3.99	1.74	1.88		1.42	2.01	1.96		0.49		0.13			3.79	3.82	1.74	1.62		1.42	1.56	1.96	2.247	0.49	0.453	0.13	0.108
129	4.68	4.88	1.13	1.27		1.78	2.37	1.72		0.53		0.13			4.68	4.71	1.13	1.01		1.78	1.92	1.72	2.007	0.53	0.493	0.13	0.108
130	3.91	4.11	1.92	2.06		1.42	2.01	2.31		0.52		0.9			3.91	3.94	1.92	1.8		1.42	1.56	2.31	2.597	0.52	0.483	0.9	0.878
131	4.82	5.02	1.74	1.88		1.43	2.02	1.96		0.51		0.13			4.82	4.85	1.74	1.62		1.43	1.57	1.96	2.247	0.51	0.473	0.13	0.108
132	4.96	5.16	0.91	1.05		1.42	2.01	2.73		0.54		0.13			4.96	4.99	0.91	0.79		1.42	1.56	2.73	3.017	0.54	0.503	0.13	0.108
133	4.51	4.71	1.51	1.65		1.61	2.2	2.69		0.49		0.13			4.51	4.54	1.51	1.39		1.61	1.75	2.69	2.977	0.49	0.453	0.13	0.108
134	3.88	4.08	1.74	1.88		1.42	2.01	1.96		0.51		0.13			3.88	3.91	1.74	1.62		1.42	1.56	1.96	2.247	0.51	0.473	0.13	0.108
135	4.36	4.56	1.62	1.76		1.71	2.3	2.31		0.45		0.9			4.36	4.39	1.62	1.5		1.71	1.85	2.31	2.597	0.45	0.413	0.9	0.878
136	4.96	5.16	1.92	2.06		1.42	2.01	2.64		0.44		0.13			4.96	4.99	1.92	1.8		1.42	1.56	2.64	2.927	0.44	0.403	0.13	0.108
137	4.51	4.71	1.74	1.88		1.78	2.37	1.96		0.49		0.13			4.51	4.54	1.74	1.62		1.78	1.92	1.96	2.247	0.49	0.453	0.13	0.108
138	3.88	4.08	1.36	1.5		1.42	2.01	1.72		0.51		0.9			3.88	3.91	1.36	1.24		1.42	1.56	1.72	2.007	0.51	0.473	0.9	0.878
139	4.96	5.16	0.99	1.13		1.78	2.37	2.73		0.53		0.13			4.96	4.99	0.99	0.87		1.78	1.92	2.73	3.017	0.53	0.493	0.13	0.108
140	4.96	5.16	1.74	1.88		1.42	2.01	1.96		0.54		0.13			4.96	4.99	1.74	1.62		1.42	1.56	1.96	2.247	0.54	0.503	0.13	0.108
141	4.51	4.71	0.91	1.05		1.92	2.51	1.72		0.49		0.9			4.51	4.54	0.91	0.79		1.92	2.06	1.72	2.007	0.49	0.453	0.9	0.878
142	3.91	4.11	1.92	2.06		1.71	2.3	2.31		0.51		0.13			3.91	3.94	1.92	1.8		1.71	1.85	2.31	2.597	0.51	0.473	0.13	0.108
143	4.96	5.16	1.74	1.88		1.43	2.02	1.96		0.45		0.13			4.96	4.99	1.74	1.62		1.43	1.57	1.96	2.247	0.45	0.413	0.13	0.108
144	4.82	5.02	0.79	0.93		1.42	2.01	1.72		0.47		0.13			4.82	4.85	0.79	0.67		1.42	1.56	1.72	2.007	0.47	0.433	0.13	0.108
145	4.51	4.71	1.62	1.76		1.61	2.2	2.69		0.49		0.13			4.51	4.54	1.62	1.5		1.61	1.75	2.69	2.977	0.49	0.453	0.13	0.108
146	3.96	4.16	1.74	1.88		1.78	2.37	1.96		0.54		0.13			3.96	3.99	1.74	1.62		1.78	1.92	1.96	2.247	0.54	0.503	0.13	0.108
147	4.68	4.88	1.51	1.65		1.71	2.3	2.61		0.53		0.13			4.68	4.71	1.51	1.39		1.71	1.85	2.61	2.897	0.53	0.493	0.13	0.108
148	3.91	4.11	0.89	1.03		1.42	2.01	1.72		0.51		0.9			3.91	3.94	0.89	0.77		1.42	1.56	1.72	2.007	0.51	0.473	0.9	0.878
149	3.88	4.08	1.74	1.88		1.76	2.35	1.96		0.49		0.13			3.88	3.91	1.74	1.62		1.76	1.9	1.96	2.247	0.49	0.453	0.13	0.108
150	3.79	3.99	1.92	2.06		1.43	2.02	1.72		0.45		0.13			3.79	3.82	1.92	1.8		1.43	1.57	1.72	2.007	0.45	0.413	0.13	0.108
151	4.82	5.02	1.13	1.27		1.78	2.37	2.31		0.54		0.13			4.82	4.85	1.13	1.01		1.78	1.92	2.31	2.597	0.54	0.503	0.13	0.108
152	4.36	4.56	1.74	1.88		1.42	2.01	1.96		0.51		0.13			4.36	4.39	1.74	1.62		1.42	1.56	1.96	2.247	0.51	0.473	0.13	0.108
153	3.91	4.11	0.79	0.93		1.61	2.2	1.72		0.49		0.9			3.91	3.94	0.79	0.67		1.61	1.75	1.72	2.007	0.49	0.453	0.9	0.878
154	4.96	5.16	0.91	1.05		1.59	2.18	2.43		0.56		0.13			4.96	4.99	0.91	0.79		1.59	1.73	2.43	2.717	0.56	0.523	0.13	0.108
155	4.17	4.37	1.74	1.88		1.43	2.02	1.96		0.44		0.13			4.17	4.2	1.74	1.62		1.43	1.57	1.96	2.247	0.44	0.403	0.13	0.108
156	4.96	5.16	1.36	1.5		1.42	2.01	1.72		0.51		0.9			4.96	4.99	1.36	1.24		1.42	1.56	1.72	2.007	0.51	0.473	0.9	0.878
157	4.68	4.88	1.92	2.06		1.47	2.06	2.69		0.54		0.13			4.68	4.71	1.92	1.8		1.47	1.61	2.69	2.977	0.54	0.503	0.13	0.108
158	4.51	4.71	1.74	1.88		1.71	2.3	1.96		0.49		0.13			4.51	4.54	1.74	1.62		1.71	1.85	1.96	2.247	0.49	0.453	0.13	0.108
159	3.91	4.11	0.99	1.13		1.92	2.51	1.72		0.45		0.9			3.91	3.94	0.99	0.87		1.92	2.06	1.72	2.007	0.45	0.413	0.9	0.878
160	4.82	5.02	1.51	1.65		1.42	2.01	2.31		0.49		0.13			4.82	4.85	1.51	1.39		1.42	1.56	2.31	2.597	0.49	0.453	0.13	0.108
161	3.79	3.99	0.79	0.93		1.61	2.2	1.96		0.53		0.13			3.79	3.82	0.79	0.67		1.61	1.75	1.96	2.247	0.53	0.493	0.13	0.108
162	3.88	4.08	1.13	1.27		1.71	2.3	1.72		0.51		0.13			3.88	3.91	1.13	1.01		1.71	1.85	1.72	2.007	0.51	0.473	0.13	0.108
163	4.96	5.16	1.92	2.06		1.47	2.06	2.64		0.54		0.13			4.96	4.99	1.92	1.8		1.47	1.61	2.64	2.927	0.54	0.503	0.13	0.108
164	4.17	4.37	1.74	1.88		1.71	2.3	1.96		0.49		0.13			4.17	4.2	1.74	1.62		1.71	1.85	1.96	2.247	0.49	0.453	0.13	0.108
165	4.96	5.16	0.91	1.05		1.78	2.37	1.72		0.51		0.13			4.96	4.99	0.91	0.79		1.78	1.92	1.72	2.007	0.51	0.473	0.13	0.108
166	4.36	4.56	1.64	1.78		1.59	2.18	2.64		0.53		0.9			4.36	4.39	1.64	1.52		1.59	1.73	2.64	2.927	0.53	0.493	0.9	0.878
167	4.68	4.88	1.36	1.5		1.76	2.35	1.96		0.45		0.13			4.68	4.71	1.36	1.24		1.76	1.9	1.96	2.247	0.45	0.413	0.13	0.108
168	4.96	5.16	0.79	0.93		1.61	2.2	1.72		0.49		0.13			4.96	4.99	0.79	0.67		1.61	1.75	1.72	2.007	0.49	0.453	0.13	0.108
169	3.88	4.08	1.92	2.06		1.71	2.3	2.31		0.51		0.9			3.88	3.91	1.92	1.8		1.71	1.85	2.31	2.597	0.51	0.473	0.9	0.878
170	3.79	3.99	1.74	1.88		1.78	2.37	1.96		0.52		0.13			3.79	3.82	1.74	1.62		1.78	1.92	1.96	2.247	0.52	0.483	0.13	0.108
171	3.88	4.08	1.51	1.65		1.92	2.51	1.72		0.51		0.13			3.88	3.91	1.51	1.39		1.92	2.06	1.72	2.007	0.51	0.473	0.13	0.108
172	4.96	5.16	0.89	1.03		1.42	2.01	2.69		0.54		0.13			4.96	4.99	0.89	0.77		1.42	1.56	2.69	2.977	0.54	0.503	0.13	0.108
173	4.51	4.71	0.91	1.05		1.71	2.3	1.96		0.49		0.9			4.51	4.54	0.91	0.79		1.71	1.85	1.96	2.247	0.49	0.453	0.9	0.878
174	4.96	5.16	1.92	2.06		1.66	2.25	1.72		0.51		0.13			4.96	4.99	1.92	1.8		1.66	1.8	1.72	2.007	0.51	0.473	0.13	0.108
175	4.49	4.69	0.79	0.93		1.47	2.06	2.31		0.47		0.13			4.49	4.52	0.79	0.67		1.47	1.61	2.31	2.597	0.47	0.433	0.13	0.108
176	4.82	5.02	1.74	1.88		1.42	2.01	1.96		0.54		0.9			4.82	4.85	1.74	1.62		1.42	1.56	1.96	2.247	0.54	0.503	0.9	0.878
177	4.51	4.71	1.62	1.76		1.61	2.2	1.72		0.51		0.13			4.51	4.54	1.62	1.5		1.61	1.75	1.72	2.007	0.51	0.473	0.13	0.108
178	4.36	4.56	1.13	1.27		1.43	2.02	2.61		0.45		0.13			4.36	4.39	1.13	1.01		1.43	1.57	2.61	2.897	0.45	0.413	0.13	0.108
179	3.88	4.08	1.13	1.27		1.71	2.3	1.96		0.49		0.9			3.88	3.91	1.13	1.01		1.71	1.85	1.96	2.247	0.49	0.453	0.9	0.878
180	4.96	5.16	1.92	2.06		1.42	2.01	2.76		0.51		0.13			4.96	4.99	1.92	1.8		1.42	1.56	2.76	3.047	0.51	0.473	0.13	0.108
181	4.51	4.71	1.51	1.65		1.92	2.51	1.72		0.54		0.13			4.51	4.54	1.51	1.39		1.92	2.06	1.72	2.007	0.54	0.503	0.13	0.108
182	3.88	4.08	1.74	1.88		1.78	2.37	1.96		0.53		0.9			3.88	3.91	1.74	1.62		1.78	1.92	1.96	2.247	0.53	0.493	0.9	0.878
183	3.79	3.99	0.79	0.93		1.59	2.18	2.69		0.51		0.13			3.79	3.82	0.79	0.67		1.59	1.73	2.69	2.977	0.51	0.473	0.13	0.108
184	4.82	5.02	0.91	1.05		1.71	2.3	2.31		0.49		0.13			4.82	4.85	0.91	0.79		1.71	1.85	2.31	2.597	0.49	0.453	0.13	0.108
185	3.96	4.16	1.62	1.76		1.76	2.35	1.96		0.44		0.13			3.96	3.99	1.62	1.5		1.76	1.9	1.96	2.247	0.44	0.403	0.13	0.108
186	4.96	5.16	1.92	2.06		1.78	2.37	1.72		0.51		0.9			4.96	4.99	1.92	1.8		1.78	1.92	1.72	2.007	0.51	0.473	0.9	0.878
187	4.36	4.56	1.36	1.5		1.43	2.02	2.76		0.54		0.13			4.36	4.39	1.36	1.24		1.43	1.57	2.76	3.047	0.54	0.503	0.13	0.108
188	3.88	4.08	1.74	1.88		1.43	2.02	1.96		0.49		0.13			3.88	3.91	1.74	1.62		1.43	1.57	1.96	2.247	0.49	0.453	0.13	0.108
189	3.91	4.11	0.91	1.05		1.61	2.2	1.72		0.51		0.9			3.91	3.94	0.91	0.79		1.61	1.75	1.72	2.007	0.51	0.473	0.9	0.878
190	4.17	4.37	0.79	0.93		1.71	2.3	2.61		0.45		0.13			4.17	4.2	0.79	0.67		1.71	1.85	2.61	2.897	0.45	0.413	0.13	0.108
191	4.82	5.02	0.99	1.13		1.43	2.02	1.96		0.56		0.13			4.82	4.85	0.99	0.87		1.43	1.57	1.96	2.247	0.56	0.523	0.13	0.108
192	4.96	5.16	1.92	2.06		1.43	2.02	2.31		0.51		0.13			4.96	4.99	1.92	1.8		1.43	1.57	2.31	2.597	0.51	0.473	0.13	0.108
193	3.88	4.08	1.51	1.65		1.66	2.25	1.72		0.49		0.9			3.88	3.91	1.51	1.39		1.66	1.8	1.72	2.007	0.49	0.453	0.9	0.878
194	3.88	4.08	1.74	1.88		1.78	2.37	1.96		0.47		0.13			3.88	3.91	1.74	1.62		1.78	1.92	1.96	2.247	0.47	0.433	0.13	0.108
195	4.96	5.16	1.62	1.76		1.42	2.01	2.69		0.51		0.13			4.96	4.99	1.62	1.5		1.42	1.56	2.69	2.977	0.51	0.473	0.13	0.108
196	4.36	4.56	1.36	1.5		1.61	2.2	2.64		0.45		0.13			4.36	4.39	1.36	1.24		1.61	1.75	2.64	2.927	0.45	0.413	0.13	0.108
197	4.51	4.71	0.79	0.93		1.71	2.3	1.96		0.54		0.9			4.51	4.54	0.79	0.67		1.71	1.85	1.96	2.247	0.54	0.503	0.9	0.878
198	4.96	5.16	1.74	1.88		1.92	2.51	1.72		0.51		0.9			4.96	4.99	1.74	1.62		1.92	2.06	1.72	2.007	0.51	0.473	0.9	0.878
199	4.82	5.02	1.92	2.06		1.42	2.01	2.31		0.49		0.13			4.82	4.85	1.92	1.8		1.42	1.56	2.31	2.597	0.49	0.453	0.13	0.108
200	3.79	3.99	1.74	1.88		1.66	2.25	1.96		0.44		0.13			3.79	3.82	1.74	1.62		1.66	1.8	1.96	2.247	0.44	0.403	0.13	0.108
201	4.51	4.71	0.91	1.05		1.71	2.3	1.72		0.51		0.13			4.51	4.54	0.91	0.79		1.71	1.85	1.72	2.007	0.51	0.473	0.13	0.108
202	3.96	4.16	1.62	1.76		1.59	2.18	2.43		0.54		0.13			3.96	3.99	1.62	1.5		1.59	1.73	2.43	2.717	0.54	0.503	0.13	0.108
203	3.88	4.08	1.74	1.88		1.42	2.01	1.96		0.49		0.13			3.88	3.91	1.74	1.62		1.42	1.56	1.96	2.247	0.49	0.453	0.13	0.108
204	4.96	5.16	1.51	1.65		1.92	2.51	2.69		0.51		0.13			4.96	4.99	1.51	1.39		1.92	2.06	2.69	2.977	0.51	0.473	0.13	0.108
205	4.51	4.71	0.79	0.93		1.61	2.2	1.72		0.53		0.13			4.51	4.54	0.79	0.67		1.61	1.75	1.72	2.007	0.53	0.493	0.13	0.108
206	3.91	4.11	1.92	2.06		1.47	2.06	1.96		0.45		0.9			3.91	3.94	1.92	1.8		1.47	1.61	1.96	2.247	0.45	0.413	0.9	0.878
207	4.68	4.88	1.62	1.76		1.43	2.02	2.61		0.51		0.13			4.68	4.71	1.62	1.5		1.43	1.57	2.61	2.897	0.51	0.473	0.13	0.108
208	4.82	5.02	1.74	1.88		1.42	2.01	2.76		0.49		0.13			4.82	4.85	1.74	1.62		1.42	1.56	2.76	3.047	0.49	0.453	0.13	0.108
209	4.36	4.56	1.13	1.27		1.71	2.3	1.96		0.54		0.9			4.36	4.39	1.13	1.01		1.71	1.85	1.96	2.247	0.54	0.503	0.9	0.878
210	4.36	4.56	0.91	1.05		1.78	2.37	1.72		0.51		0.13			4.36	4.39	0.91	0.79		1.78	1.92	1.72	2.007	0.51	0.473	0.13	0.108
211	4.96	5.16	0.79	0.93		1.43	2.02	1.72		0.52		0.13			4.96	4.99	0.79	0.67		1.43	1.57	1.72	2.007	0.52	0.483	0.13	0.108
212	4.36	4.56	1.92	2.06		1.43	2.02	1.96		0.49		0.9			4.36	4.39	1.92	1.8		1.43	1.57	1.96	2.247	0.49	0.453	0.9	0.878
213	3.79	3.99	1.74	1.88		1.42	2.01	2.69		0.51		0.13			3.79	3.82	1.74	1.62		1.42	1.56	2.69	2.977	0.51	0.473	0.13	0.108
214	4.82	5.02	1.36	1.5		1.61	2.2	2.31		0.47		0.13			4.82	4.85	1.36	1.24		1.61	1.75	2.31	2.597	0.47	0.433	0.13	0.108
215	3.88	4.08	1.51	1.65		1.76	2.35	1.96		0.54		0.13			3.88	3.91	1.51	1.39		1.76	1.9	1.96	2.247	0.54	0.503	0.13	0.108
216	4.96	5.16	1.62	1.76		1.71	2.3	2.64		0.51		0.9			4.96	4.99	1.62	1.5		1.71	1.85	2.64	2.927	0.51	0.473	0.9	0.878
217	4.68	4.88	0.79	0.93		1.42	2.01	1.72		0.49		0.13			4.68	4.71	0.79	0.67		1.42	1.56	1.72	2.007	0.49	0.453	0.13	0.108
218	3.88	4.08	1.74	1.88		1.47	2.06	1.96		0.45		0.13			3.88	3.91	1.74	1.62		1.47	1.61	1.96	2.247	0.45	0.413	0.13	0.108
219	4.96	5.16	1.92	2.06		1.92	2.51	2.31		0.51		0.9			4.96	4.99	1.92	1.8		1.92	2.06	2.31	2.597	0.51	0.473	0.9	0.878
220	3.91	4.11	0.91	1.05		1.78	2.37	2.61		0.53		0.13			3.91	3.94	0.91	0.79		1.78	1.92	2.61	2.897	0.53	0.493	0.13	0.108
221	4.51	4.71	0.91	1.05		1.42	2.01	1.96		0.49		0.13			4.51	4.54	0.91	0.79		1.42	1.56	1.96	2.247	0.49	0.453	0.13	0.108
222	4.82	5.02	0.89	1.03		1.61	2.2	1.72		0.51		0.9			4.82	4.85	0.89	0.77		1.61	1.75	1.72	2.007	0.51	0.473	0.9	0.878
223	4.96	5.16	1.74	1.88		1.59	2.18	2.31		0.54		0.13			4.96	4.99	1.74	1.62		1.59	1.73	2.31	2.597	0.54	0.503	0.13	0.108
224	3.79	3.99	0.79	0.93		1.71	2.3	1.72		0.44		0.13			3.79	3.82	0.79	0.67		1.71	1.85	1.72	2.007	0.44	0.403	0.13	0.108
225	4.51	4.71	1.92	2.06		1.42	2.01	2.31		0.51		0.13			4.51	4.54	1.92	1.8		1.42	1.56	2.31	2.597	0.51	0.473	0.13	0.108
226	4.49	4.69	1.51	1.65		1.66	2.25	1.72		0.49		0.13			4.49	4.52	1.51	1.39		1.66	1.8	1.72	2.007	0.49	0.453	0.13	0.108
227	3.91	4.11	1.36	1.5		1.78	2.37	1.96		0.45		0.13			3.91	3.94	1.36	1.24		1.78	1.92	1.96	2.247	0.45	0.413	0.13	0.108
228	4.36	4.56	1.74	1.88		1.92	2.51	1.72		0.51		0.9			4.36	4.39	1.74	1.62		1.92	2.06	1.72	2.007	0.51	0.473	0.9	0.878
229	4.51	4.71	1.62	1.76		1.61	2.2	2.31		0.54		0.13			4.51	4.54	1.62	1.5		1.61	1.75	2.31	2.597	0.54	0.503	0.13	0.108
230	3.79	3.99	0.79	0.93		1.76	2.35	1.96		0.49		0.13			3.79	3.82	0.79	0.67		1.76	1.9	1.96	2.247	0.49	0.453	0.13	0.108
231	4.82	5.02	1.92	2.06		1.71	2.3	1.96		0.51		0.9			4.82	4.85	1.92	1.8		1.71	1.85	1.96	2.247	0.51	0.473	0.9	0.878
232	3.88	4.08	0.91	1.05		1.59	2.18	2.69		0.53		0.13			3.88	3.91	0.91	0.79		1.59	1.73	2.69	2.977	0.53	0.493	0.13	0.108
233	4.51	4.71	1.74	1.88		1.76	2.35	1.72		0.52		0.13			4.51	4.54	1.74	1.62		1.76	1.9	1.72	2.007	0.52	0.483	0.13	0.108
234	3.91	4.11	1.36	1.5		1.42	2.01	1.96		0.51		0.13			3.91	3.94	1.36	1.24		1.42	1.56	1.96	2.247	0.51	0.473	0.13	0.108
235	4.96	5.16	1.51	1.65		1.59	2.18	2.73		0.49		0.13			4.96	4.99	1.51	1.39		1.59	1.73	2.73	3.017	0.49	0.453	0.13	0.108
236	3.96	4.16	0.79	0.93		1.61	2.2	1.72		0.54		0.13			3.96	3.99	0.79	0.67		1.61	1.75	1.72	2.007	0.54	0.503	0.13	0.108
237	4.51	4.71	1.92	2.06		1.76	2.35	1.96		0.51		0.13			4.51	4.54	1.92	1.8		1.76	1.9	1.96	2.247	0.51	0.473	0.13	0.108
238	4.17	4.37	1.74	1.88		1.42	2.01	2.69		0.45		0.13			4.17	4.2	1.74	1.62		1.42	1.56	2.69	2.977	0.45	0.413	0.13	0.108
239	4.82	5.02	1.13	1.27		1.71	2.3	2.31		0.44		0.13			4.82	4.85	1.13	1.01		1.71	1.85	2.31	2.597	0.44	0.403	0.13	0.108
240	3.91	4.11	1.62	1.76		1.78	2.37	1.96		0.51		0.11			3.91	3.94	1.62	1.5		1.78	1.92	1.96	2.247	0.51	0.473	0.11	0.088
241	4.96	5.16	1.36	1.5		1.43	2.02	1.72		0.49		0.13			4.96	4.99	1.36	1.24		1.43	1.57	1.72	2.007	0.49	0.453	0.13	0.108
242	4.51	4.71	0.79	0.93		1.92	2.51	2.43		0.54		0.9			4.51	4.54	0.79	0.67		1.92	2.06	2.43	2.717	0.54	0.503	0.9	0.878
243	3.88	4.08	1.74	1.88		1.61	2.2	1.96		0.51		0.13			3.88	3.91	1.74	1.62		1.61	1.75	1.96	2.247	0.51	0.473	0.13	0.108
244	4.36	4.56	1.92	2.06		1.76	2.35	2.61		0.53		0.13			4.36	4.39	1.92	1.8		1.76	1.9	2.61	2.897	0.53	0.493	0.13	0.108
245	4.96	5.16	0.91	1.05		1.71	2.3	1.72		0.49		0.9			4.96	4.99	0.91	0.79		1.71	1.85	1.72	2.007	0.49	0.453	0.9	0.878
246	3.79	3.99	1.51	1.65		1.47	2.06	1.96		0.45		0.11			3.79	3.82	1.51	1.39		1.47	1.61	1.96	2.247	0.45	0.413	0.11	0.088
247	4.82	5.02	1.13	1.27		1.59	2.18	2.31		0.54		0.13			4.82	4.85	1.13	1.01		1.59	1.73	2.31	2.597	0.54	0.503	0.13	0.108
248	4.68	4.88	1.74	1.88		1.76	2.35	2.69		0.49		0.13			4.68	4.71	1.74	1.62		1.76	1.9	2.69	2.977	0.49	0.453	0.13	0.108
249	4.96	5.16	0.79	0.93		1.61	2.2	1.96		0.51		0.9			4.96	4.99	0.79	0.67		1.61	1.75	1.96	2.247	0.51	0.473	0.9	0.878
250	3.96	4.16	1.92	2.06		1.42	2.01	1.72		0.44		0.13			3.96	3.99	1.92	1.8		1.42	1.56	1.72	2.007	0.44	0.403	0.13	0.108
251	4.82	5.02	1.62	1.76		1.78	2.37	2.36		0.47		0.13			4.82	4.85	1.62	1.5		1.78	1.92	2.36	2.647	0.47	0.433	0.13	0.108
252	3.88	4.08	1.64	1.78		1.71	2.3	1.96		0.49		0.9			3.88	3.91	1.64	1.52		1.71	1.85	1.96	2.247	0.49	0.453	0.9	0.878
253	3.91	4.11	1.74	1.88		1.76	2.35	2.31		0.54		0.13			3.91	3.94	1.74	1.62		1.76	1.9	2.31	2.597	0.54	0.503	0.13	0.108
254	4.51	4.71	1.92	2.06		1.42	2.01	1.72		0.45		0.13			4.51	4.54	1.92	1.8		1.42	1.56	1.72	2.007	0.45	0.413	0.13	0.108
255	4.82	5.02	0.79	0.93		1.43	2.02	1.96		0.49		0.13			4.82	4.85	0.79	0.67		1.43	1.57	1.96	2.247	0.49	0.453	0.13	0.108
256	3.79	3.99	0.89	1.03		1.92	2.51	2.61		0.51		0.13			3.79	3.82	0.89	0.77		1.92	2.06	2.61	2.897	0.51	0.473	0.13	0.108
257	4.82	5.02	0.91	1.05		1.61	2.2	2.31		0.53		0.9			4.82	4.85	0.91	0.79		1.61	1.75	2.31	2.597	0.53	0.493	0.9	0.878
258	4.96	5.16	1.74	1.88		1.42	2.01	1.96		0.54		0.13			4.96	4.99	1.74	1.62		1.42	1.56	1.96	2.247	0.54	0.503	0.13	0.108
259	3.91	4.11	1.92	2.06		1.47	2.06	1.72		0.51		0.13			3.91	3.94	1.92	1.8		1.47	1.61	1.72	2.007	0.51	0.473	0.13	0.108
260	4.51	4.71	1.51	1.65		1.71	2.3	2.69		0.49		0.9			4.51	4.54	1.51	1.39		1.71	1.85	2.69	2.977	0.49	0.453	0.9	0.878
261	4.96	5.16	0.79	0.93		1.92	2.51	1.72		0.44		0.13			4.96	4.99	0.79	0.67		1.92	2.06	1.72	2.007	0.44	0.403	0.13	0.108
262	3.96	4.16	1.62	1.76		1.42	2.01	2.61		0.51		0.13			3.96	3.99	1.62	1.5		1.42	1.56	2.61	2.897	0.51	0.473	0.13	0.108
263	4.68	4.88	1.74	1.88		1.43	2.02	2.31		0.54		0.13			4.68	4.71	1.74	1.62		1.43	1.57	2.31	2.597	0.54	0.503	0.13	0.108
264	4.82	5.02	1.92	2.06		1.78	2.37	1.96		0.49		0.9			4.82	4.85	1.92	1.8		1.78	1.92	1.96	2.247	0.49	0.453	0.9	0.878
265	3.91	4.11	1.62	1.76		1.61	2.2	1.72		0.45		0.13			3.91	3.94	1.62	1.5		1.61	1.75	1.72	2.007	0.45	0.413	0.13	0.108
266	4.36	4.56	0.89	1.03		1.59	2.18	2.64		0.51		0.13			4.36	4.39	0.89	0.77		1.59	1.73	2.64	2.927	0.51	0.473	0.13	0.108
267	3.88	4.08	0.79	0.93		1.42	2.01	2.69		0.45		0.9			3.88	3.91	0.79	0.67		1.42	1.56	2.69	2.977	0.45	0.413	0.9	0.878
268	4.51	4.71	1.74	1.88		1.71	2.3	1.72		0.49		0.13			4.51	4.54	1.74	1.62		1.71	1.85	1.72	2.007	0.49	0.453	0.13	0.108
269	4.36	4.56	1.92	2.06		1.43	2.02	2.31		0.54		0.13			4.36	4.39	1.92	1.8		1.43	1.57	2.31	2.597	0.54	0.503	0.13	0.108
270	4.96	5.16	1.62	1.76		1.76	2.35	1.96		0.49		0.9			4.96	4.99	1.62	1.5		1.76	1.9	1.96	2.247	0.49	0.453	0.9	0.878
271	3.91	4.11	0.91	1.05		1.92	2.51	2.61		0.53		0.13			3.91	3.94	0.91	0.79		1.92	2.06	2.61	2.897	0.53	0.493	0.13	0.108
272	3.79	3.99	1.92	2.06		1.61	2.2	1.72		0.51		0.13			3.79	3.82	1.92	1.8		1.61	1.75	1.72	2.007	0.51	0.473	0.13	0.108
273	4.82	5.02	1.74	1.88		1.71	2.3	2.43		0.49		0.13			4.82	4.85	1.74	1.62		1.71	1.85	2.43	2.717	0.49	0.453	0.13	0.108
274	4.68	4.88	0.79	0.93		1.42	2.01	1.72		0.54		0.13			4.68	4.71	0.79	0.67		1.42	1.56	1.72	2.007	0.54	0.503	0.13	0.108
275	4.96	5.16	1.51	1.65		1.43	2.02	2.31		0.51		0.9			4.96	4.99	1.51	1.39		1.43	1.57	2.31	2.597	0.51	0.473	0.9	0.878
276	3.91	4.11	1.64	1.78		1.61	2.2	1.96		0.51		0.13			3.91	3.94	1.64	1.52		1.61	1.75	1.96	2.247	0.51	0.473	0.13	0.108
277	4.96	5.16	1.62	1.76		1.42	2.01	2.69		0.45		0.13			4.96	4.99	1.62	1.5		1.42	1.56	2.69	2.977	0.45	0.413	0.13	0.108
278	4.68	4.88	1.74	1.88		1.42	2.01	1.72		0.49		0.9			4.68	4.71	1.74	1.62		1.42	1.56	1.72	2.007	0.49	0.453	0.9	0.878
279	4.36	4.56	0.91	1.05		1.43	2.02	2.69		0.56		0.13			4.36	4.39	0.91	0.79		1.43	1.57	2.69	2.977	0.56	0.523	0.13	0.108
280	4.82	5.02	0.79	0.93		1.78	2.37	1.72		0.54		0.13			4.82	4.85	0.79	0.67		1.78	1.92	1.72	2.007	0.54	0.503	0.13	0.108
281	4.51	4.71	0.89	1.03		1.71	2.3	2.31		0.51		0.13			4.51	4.54	0.89	0.77		1.71	1.85	2.31	2.597	0.51	0.473	0.13	0.108
282	4.96	5.16	1.51	1.65		1.42	2.01	1.96		0.49		0.13			4.96	4.99	1.51	1.39		1.42	1.56	1.96	2.247	0.49	0.453	0.13	0.108
283	4.96	5.16	1.74	1.88		1.76	2.35	2.61		0.54		0.13			4.96	4.99	1.74	1.62		1.76	1.9	2.61	2.897	0.54	0.503	0.13	0.108
284	3.91	4.11	1.74	1.88		1.71	2.3	2.31		0.49		0.13			3.91	3.94	1.74	1.62		1.71	1.85	2.31	2.597	0.49	0.453	0.13	0.108
285	4.68	4.88	0.91	1.05		1.42	2.01	1.96		0.45		0.13			4.68	4.71	0.91	0.79		1.42	1.56	1.96	2.247	0.45	0.413	0.13	0.108
286	3.91	4.11	1.51	1.65		1.61	2.2	1.72		0.51		0.12			3.91	3.94	1.51	1.39		1.61	1.75	1.72	2.007	0.51	0.473	0.12	0.098
287	4.82	5.02	1.92	2.06		1.71	2.3	1.96		0.49		0.9			4.82	4.85	1.92	1.8		1.71	1.85	1.96	2.247	0.49	0.453	0.9	0.878
288	4.51	4.71	1.74	1.88		1.42	2.01	1.72		0.51		0.13			4.51	4.54	1.74	1.62		1.42	1.56	1.72	2.007	0.51	0.473	0.13	0.108
		4.629166667				1.608159722	2.198159722											1.3059375		1.60815972	1.74815972	2.148229167	2.435229167	0.501631944	0.464631944	0.357083333	0.335083333
